# Multilocus basis of incipient reproductive isolation in hybridizing populations is revealed by pangenomic and epigenetic divergence

**DOI:** 10.1126/sciadv.adz6665

**Published:** 2026-08-21

**Authors:** Angelo A. Ruggieri, Francesco Cicconardi, Nicolò Bellin, Stephen H. Montgomery, James Mallet, Steven M. Van Belleghem, Owen W. McMillan, Brian A. Counterman, Riccardo Papa

**Affiliations:** ^1^ Department of Biology, University of Puerto Rico, Rio Piedras, Puerto Rico.; ^2^ School of Biological Sciences, Bristol University, Bristol, United Kingdom.; ^3^ Organismal and Evolutionary Biology, Harvard University, Cambridge, MA 02138, USA.; ^4^ Ecology, Evolution, and Conservation Biology, Biology Department, KU Leuven, Leuven, Belgium.; ^5^ Smithsonian Tropical Research Institute, Panama, Panama.; ^6^ Department of Biological Sciences, Auburn University, Auburn, AL 36849, USA.; ^7^ Molecular Sciences and Research Center, University of Puerto Rico, San Juan, Puerto Rico.; ^8^ Comprehensive Cancer Center, University of Puerto Rico, San Juan, Puerto Rico.; ^9^ Dipartimento di Scienze chimiche della vita e della Sostenibilità Ambientale, Università di Parma, Parma 43124, Italy.

## Abstract

Incipient reproductive isolation in the presence of gene flow has traditionally been attributed to a small number of major-effect loci under strong selection. Here, using the *Heliconius erato* adaptive radiation, we apply a pangenome framework to examine how mutational divergence, regulatory variation, and structural variants contribute to genome-wide divergence. In contrast to earlier studies, our high-resolution analyses reveal widespread divergence across the genome, consistent with a multilocus barrier to gene flow. Our findings support a model in which selection acts on regulatory phenotypes under migration-selection balance, with genetic differentiation becoming more pronounced as gene flow declines. By integrating population-level sampling, we show that apparent population-specific structural and regulatory variation inferred from single-reference genomes is overestimated, reflecting pervasive reference bias. While structural variants contribute to genomic variation, in our system, most are shared or polymorphic rather than fixed differences between populations. Together, our results show that the genomic landscape of *H. erato* divergence reflects the combined contributions of regulatory variation and mutational change, while highlighting the importance of accounting for reference bias when interpreting structural and regulatory divergence. This multilocus framework provides a more accurate view of how reproductive barriers emerge and strengthen under ongoing gene flow.

## INTRODUCTION

Early genetic models of speciation often emphasized a limited number of loci of large effect, particularly those involved in reproductive incompatibilities, as the primary drivers of species boundaries. Subsequent theoretical and empirical work has expanded this view, showing that reproductive isolation can emerge from the combined effects of many loci acting across the genome, especially under conditions of ongoing gene flow. In this framework, population-level genetic divergence is a central component of speciation, with notable exceptions such as hybrid speciation, in which new species arise through hybridization between divergent parental lineages (*1*, *2*). During the early stages of divergence, incipient reproductive isolation may be maintained by selection acting on specific barrier loci associated with morphological, behavioral, reproductive, or ecological traits, often referred to as “speciation genes” (*3*, *4*). Over time, the accumulation of additional genetic and regulatory differences can reinforce these barriers and promote more stable reproductive isolation.

Population genomics has been central to identifying barrier loci and understanding how they persist during early speciation. A promising approach involves studying populations that exhibit local adaptation and phenotypic divergence despite ongoing gene flow, especially in sympatric and parapatric contexts (*5*–*8*). In such cases, divergence tends to be concentrated at loci under strong selection, forming “genomic islands of speciation,” regions of elevated differentiation that emerge when divergent selection counteracts gene flow (*4*). Over time, hitchhiking and linkage may allow divergence to spread, reinforcing isolation across broader genomic regions.

While Genome-Wide Association Study (GWAS) and fine-scale mapping have improved our understanding of these barriers, major gaps remain, particularly in identifying the number and functional roles of contributing loci. Structural variants (SVs), particularly small-scale variants, may be underappreciated drivers of regulatory divergence and speciation, especially given the difficulty of detecting noncoding regulatory elements in nonmodel organisms (*9*). However, recent advances, such as chromatin accessibility assays [e.g., Assay for Transposase-Accessible Chromatin using sequencing (ATAC-seq); (*10*)], population-specific genome assemblies, and improved computational tools, are beginning to close these gaps.

Hybrid zones, where diverging populations interbreed and produce intermediate phenotypes, provide powerful natural laboratories for studying early genomic divergence (*11*, *12*). The *Heliconius* genus, with over 40 species, hundreds of divergent mimetic subspecies across diverse hybrid zones, and varying levels of reproductive isolation, is a leading model for studying the genetics of incipient speciation. Prior work has shown that introgression, selection, and assortative mating contribute to barriers between populations (*13*, *14*). In *Heliconius erato*, population genetic studies have suggested that divergence is largely limited to a few color pattern loci (*15*–*17*). While these genes are critical for mimicry, predator avoidance, and mating, it is unlikely that the rest of the genome remains completely homogeneous, especially given known behavioral, metabolic, and ecological adaptations (*18*).

In this study, we extend the analysis of genome-wide divergence by integrating de novo genome assemblies, chromatin accessibility data, and improved gene annotations across *H. erato* populations with varying levels of geographic isolation. Our findings reveal a multilocus architecture spanning both genetic and epigenetic variation that highlights the complexity of incipient reproductive isolation. This polygenic framework suggests that genome-wide divergence emerges earlier and more broadly than previously assumed, challenging the classical view of speciation driven by a few isolated genomic islands.

## RESULTS

### Genome assemblies, pangenome alignment, and lineage-specific sequence composition

**Fig. 1.**
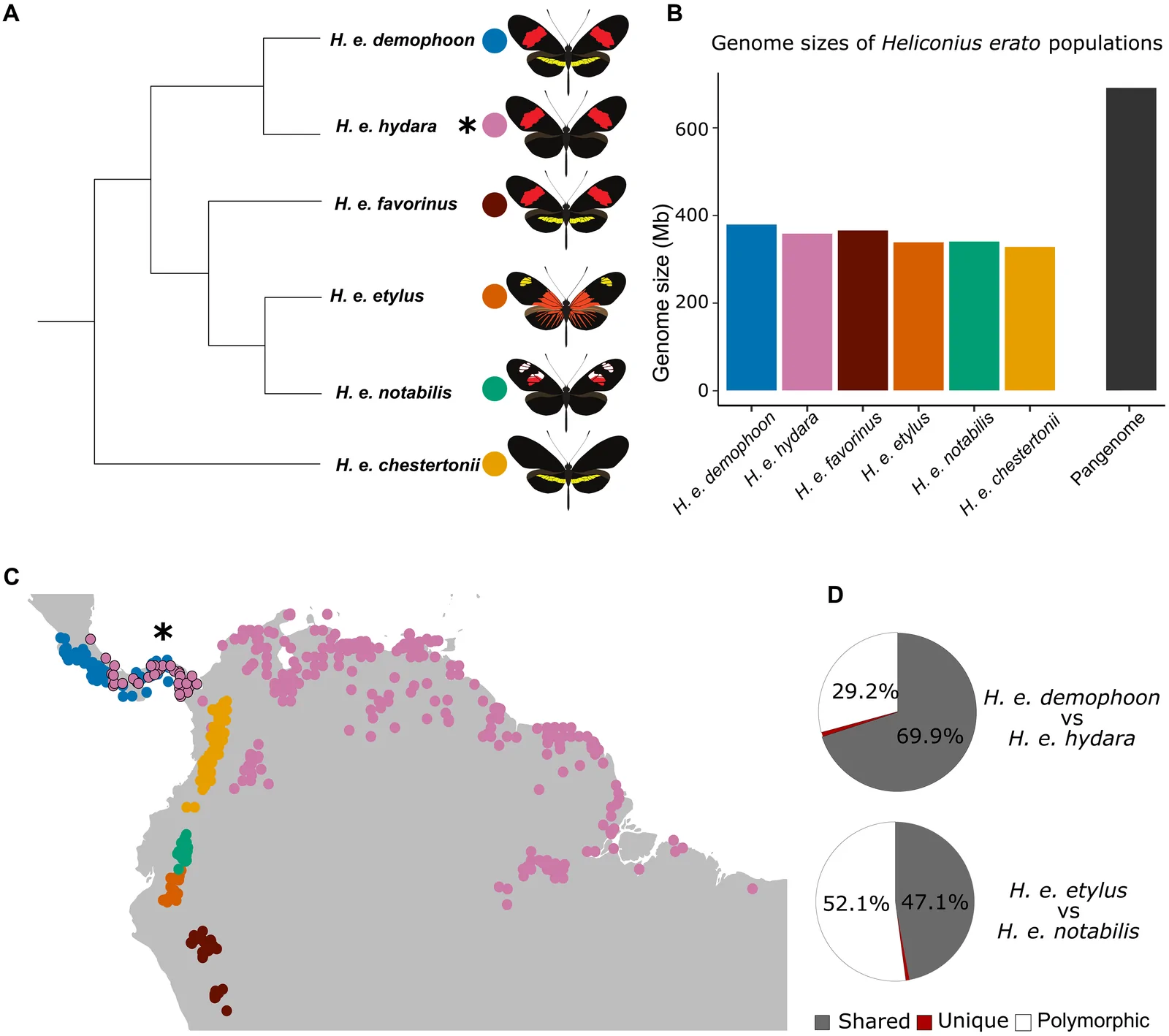
Geographic distribution, phylogenetic relationship of geographical populations, and pangenome overview. (**A**) Dendrogram showing the phylogenetic relationships among the geographical mimetic populations of *H. erato* used in this study. (**B**) Pangenome overview generated from the six geographical populations. The length of each original genome is represented by a distinct color, whereas black indicates the total length of the pangenome. (**C**) Geographic distribution of the populations based on sampling data obtained from Heliconius Maps [https://heliconius-maps.github.io/; Rosser and Mallet (*14*)]. The asterisk shows where the specific samples of *H. e. hydara* used in this study were sampled from. (**D**) Distribution of ATAC-seq peaks in forewing between adjacent populations. “Shared” indicates peaks consistently present in both populations, “polymorphic” indicates peaks not consistently present in all samples of a population, and “unique” indicates peaks identified only in one population.

We generated new genome assemblies for five *H. erato* populations: *H. e. notabilis*, *H. e. etylus*, *H. e. hydara*, *H. e. chestertonii*, and *H. e. favorinus*, and integrated them with the previously published *H. e. demophoon* genome to construct a comprehensive linear pangenome (Fig. 1, A and C). The *H. e. demophoon* genome spans 377.2 Mb, and the remaining genomes show limited size variation, all within 13% of this reference. Among them, *H. e. chestertonii* has the smallest genome at 326.2 Mb, while *H. e. hydara*, *H. e. favorinus*, *H. e. etylus*, and *H. e. notabilis* span 356.5, 363.7, 336.7, and 338.4 Mb, respectively (Fig. 1B and table S1).

The assembled pangenome spans 688 Mb and represents an extended reference based on the *H. e. demophoon* genome, incorporating population-specific sequences absent from the single reference. This pangenome was used to align both resequencing and ATAC-seq data (Fig. 1B). To evaluate potential mapping biases, we compared alignments to the single reference and the pangenome across representative samples from each population. As expected, because the pangenome is approximately twice the length of the original *H. erato* reference genome, the call rate, variant density, and mean sequencing depth were consistently lower in the pangenome-based analysis compared to the single-reference approach, with a corresponding increase in missing data (table S2). Across samples, call rate decreased from ∼0.62 to 0.74 in the single reference to ∼0.37 to 0.45 in the pangenome, while variant density was reduced from ∼0.14 to ∼0.11–0.12. Similarly, mean sequencing depth declined from ∼18 to 33× in the single reference to ∼12 to 24× under the pangenome framework. In contrast, mapping rates remained high and slightly improved in the pangenome (generally >98% versus ∼92 to 97% in the single reference), with comparable error rates between approaches. Despite the larger genomic space and increased missing data, the total number of detected variants remained similar between approaches, with a slight tendency toward higher counts in the pangenome in several samples (table S2). Notably, regions unique in the pangenome, present in the pangenome but not in the single-genome reference, still contribute meaningful variation, as they show comparable levels of genetic differentiation (*F_st_*) to shared regions, including in the *H. e. demophoon* versus *H. e. hydara* comparison (fig. S1).

This indicates that variants are distributed across a larger genomic space rather than being lost due to mapping ambiguity, suggesting that the pangenome does not introduce substantial uncertainty in read placement but instead expands the set of regions where reads can be confidently mapped. A likely explanation for the relatively modest increase in detected variants is the reduction in sequencing depth under the pangenome framework, which may render filtering thresholds slightly too stringent and lead to the exclusion of additional variation. This reduction in depth should therefore be considered in future analyses, either by increasing sequencing effort or by adjusting downstream filtering criteria.

### Genes under selective forces between adjacent subspecies

This project focused on identifying fine-scale signals of sequence divergence potentially associated with regulatory elements; therefore, *F_st_* was calculated in 1-kb windows, rather than the more traditional 50-kb windows. This strategy comes with both strengths and limitations. Interpreting *F_st_* at very small genomic scales (≤1 kb) is inherently challenging due to both biological and technical sources of variance, including ongoing gene flow, heterogeneous recombination, limited sampling, and mapping uncertainty.

To address these limitations while still detecting large-scale genome-wide signals, we applied a density-based framework to distinguish true signals of divergence from background noise by identifying regions with a consistently elevated density of *F_st_* values above the genome-wide average, rather than relying solely on isolated high-*F_st_* windows. Compared to classic single-window *F_st_* scans based on genome-wide quantile thresholds, this approach yields a more conservative set of candidate regions by explicitly requiring spatial clustering of elevated differentiation. Consequently, isolated high-*F_st_* windows lacking broader genomic support are excluded. This framework is designed to detect extended signals of differentiation that are more likely to reflect regions under strong linkage disequilibrium rather than stochastic variation at individual windows. Representative high-resolution *F_st_* profiles are shown in fig. S2, with full visualization code and additional examples available on GitHub.

**Fig. 2.**
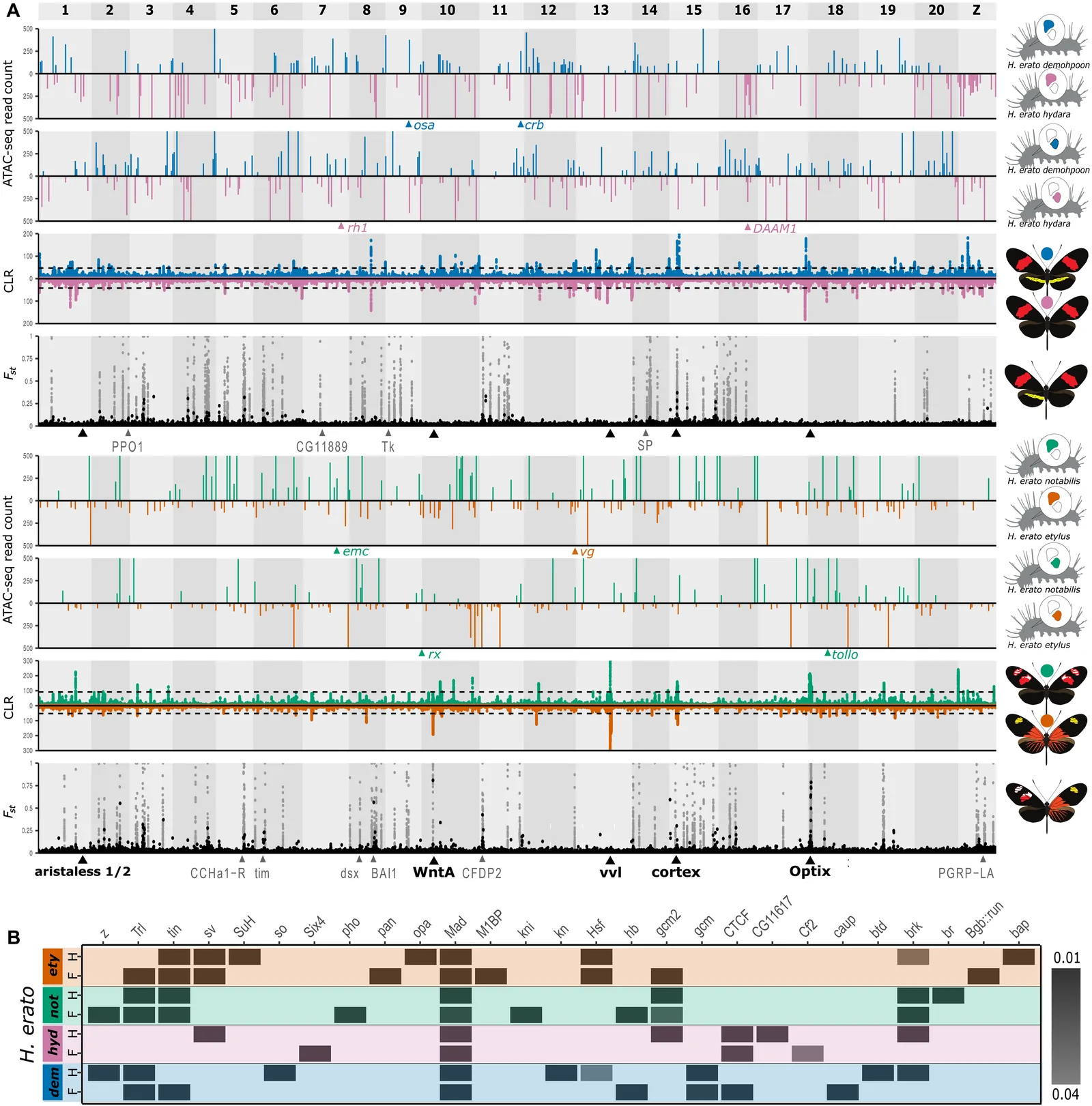
Genome-wide analysis of genetic and epigenetic data from two adjacent populations. (**A**) The first four tracks show comparisons between *H. e. demophoon* and *H. e. hydara*, while the last four tracks compare *H. e. notabilis* with *H. e. etylus*. The two ATAC-seq tracks (*y* axis = ATAC-seq read count) indicate differences in chromatin dynamics between the forewing and hindwing, focusing on ATAC-seq peaks that are unique to one population or the other. The genome was divided into 1-kb nonoverlapping windows, and for each window, the average peak read count in *H. e. hydara* was subtracted from that in *H. e. demophoon*. Results are displayed in pink (bottom half) if chromatin activity was higher in *H. e. hydara* and in blue (top half) if activity was higher in *H. e. demophoon*. The third track [*y* axis = Composite Likelihood Ratio (CLR)] represents selective sweep signals between the two populations. The fourth track (*y* axis = *F_st_*) shows *F_st_* values from population comparisons, with black dots indicating *F_st_* values calculated in 50-kb windows and gray dots indicating significant *F_st_* signals in 1-kb windows. The 1-kb *F_st_* background signal is omitted for clarity. These analyses are repeated for the comparison between *H. e. notabilis* and *H. e. etylus*, with results displayed in green and orange. The black triangle shows the classic color pattern gene’s position, the gray ones represent examples of additional candidate genes involved from the *F_st_*, and the different colored triangles on the ATAC-seq track show putative candidate genes from the ATAC-seq signal. (**B**) TF binding site enrichment analysis for each wing and each adjacent population. F, forewing; H, hindwing.

Using this approach, we identified 91 regions with elevated *F_st_* in the *demophoon-hydara* comparison (covering 2.943 Mb, or 0.427% of the pangenome) and 76 regions in *etylus-notabilis* (ranging from 53 to 280 kb and covering 2.916 Mb, or 0.423% of the pangenome; Fig. 2A). These regions also exhibit elevated *D_xy_* relative to the genomic background, a pattern that remains significant when compared to both randomly sampled loci and the full set of high-*F_st_* points (fig. S3). The co-occurrence of high *F_st_* and high *D_xy_* is consistent with reduced gene flow (*19*), suggesting that these regions are likely resistant to introgression and maintained by selection.

We quantified the relative contribution of sequence categories across the 91 high-*F_st_* regions identified in the *H. e. demophoon* versus *H. e. hydara* comparison (fig. S4). These regions exhibit substantial heterogeneity in composition, with both unique and shared sequences representing the dominant components. The proportion of unique sequences varies widely, ranging from ∼30% to nearly 80% across regions, whereas shared sequences contribute more moderately (typically ∼20 to 50%). In contrast, mixed sequences consistently represent a minor fraction, remaining within a relatively narrow range (∼5 to 15%). These results indicate that pangenome-specific (unique) sequences contribute substantially to the detected *F_st_* signals. This supports the conclusion that the inclusion of pangenome-derived sequence captures biologically meaningful variation rather than merely expanding the reference space.

Within these regions, we identified 54 genes in *demophoon-hydara* and 88 genes in *etylus-notabilis*, including well-known wing color pattern genes such as *WntA* and *optix* in *etylus-notabilis*, and *cortex* in *demophoon-hydara* (table S3). We also detected strong divergence at *doublesex*, a gene implicated in sexual dimorphism and color pattern variation across Lepidoptera (*20*). In addition, four genes linked to phenoloxidases, key components of the insect immune system, were identified (table S3) (*21*). The recovery of known wing patterning genes together with coherent *D_xy_* signals supports the ability of this framework to identify biologically meaningful regions of divergence.

Beyond classical speciation genes, we identified loci involved in chromatin modification and transcriptional regulation, including *Chromodomain-helicase-DNA-binding protein 7* and *Integrator complex subunit 7*, suggesting a potential role for chromatin dynamics in population divergence. Additional divergent genes include *timeless*, a core component of circadian rhythm regulation; *chs-2*, involved in exoskeleton formation and desiccation resistance; and genes associated with chemosensation and reproduction, such as *gustatory receptor 64f-like* and several seminal fluid proteins (HACP027, HACP057, and HACP011). These traits are known to diverge rapidly and contribute to mate recognition, reproductive isolation, and adaptation in insects (*22*, *23*). Together, these results support a multilocus architecture underlying barriers to gene flow, involving both canonical speciation genes and additional loci linked to immunity, regulation, and chromatin structure.

**Fig. 3.**
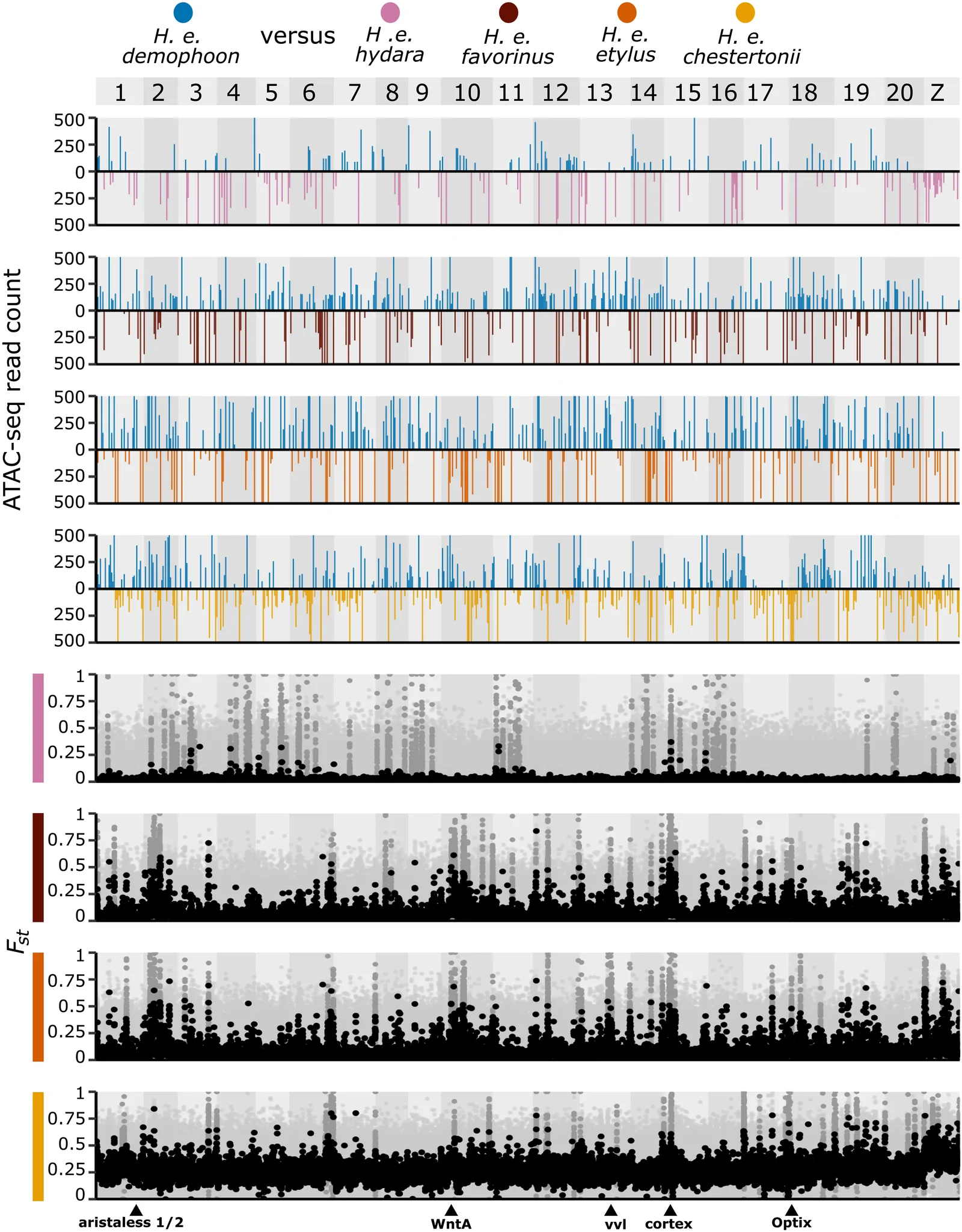
Pairwise comparisons for *H. e. demophoon*. Plots representing forewing chromatin dynamic differences and *F_st_* values, as described in Fig. 2. From top to bottom, comparisons are shown for *H. e. demophoon* versus *H. e. hydara*, *H. e. favorinus*, *H. e. etylus*, and *H. e. chestertonii*. Each *F_st_* plot displays *F_st_* values calculated in 50-kb windows (black), signals identified from 1-kb windows using custom analyses to distinguish noise from true signals (dark gray), and background noise from 1-kb windows (light gray).

Last, comparisons across increasingly divergent population pairs reveal a clear genome-wide trend (Fig. 3 and fig. S5). Both 50- and 1-kb *F_st_* estimates show a progressive increase in overall differentiation, accompanied by increasing divergence in chromatin accessibility, particularly in comparisons involving populations outside the hybrid zone. Rather than discrete islands of divergence, these patterns indicate a broad elevation of background genetic and regulatory differentiation with increasing population divergence.

### Chromatin dynamics

In addition to fine-scale *F_st_* analyses, we investigated regulatory diversity and potential barrier loci at the epigenetic level using ATAC-seq. After quality control, all ATAC-seq samples were retained (table S4). The number of ATAC-seq peaks identified per population ranged from 13,165 in *H. e. notabilis* to 22,804 in *H. e. chestertonii*. Across all populations, we detected a total of 29,633 peaks, of which 29.7% (8799) were shared across all populations, defining a conserved core of chromatin accessibility (table S1). Principal components analysis on the overall ATAC-seq peaks (fig. S6) reveals clear clustering by population. Wing tissue (forewing versus hindwing) shows only minor separation within populations, indicating that population identity is the dominant source of variation. Peaks unique to a single population were rare, accounting for only 1.48% (439) of all peaks and ranging from 0.38% (87) in *H. e. chestertonii* to 0.44% (58) in *H. e. notabilis* (table S1). These low numbers reflect our conservative definition of population specificity, which required peaks to be present in all individuals of one population and absent from all others. The majority of peaks (68.82%) were polymorphic, being present in more than one population but not fixed across all populations, indicating substantial regulatory variation despite a shared core of accessible regions.

We next examined chromatin accessibility divergence between adjacent subspecies pairs. Across the *H. e. demophoon*–*H. e. hydara* hybrid zone, 51.4 to 74.9% (7696 to 11,003) of ATAC-seq peaks were shared between populations (Fig. 1D and table S5), while fewer than 1% (117 to 161 peaks) were strictly population-specific. The remaining 29.2 to 47.5% (4594 to 7121) of peaks were polymorphic between populations, being present in at least one but not all individuals of both populations. A similar pattern was observed between the adjacent *H. e. notabilis* and *H. e. etylus* populations, where 38 to 52% (5997 to 7871) of peaks were shared, fewer than 1% (50 to 138) were unique, and 46.6 to 60.9% (6937 to 9535) were polymorphic (table S5).

Our subsequent analyses focused on the set of unique ATAC-seq peaks identified between adjacent populations, which represent fixed binary differences between populations. These peaks may correspond to particularly relevant regulatory elements as they are the most divergent between adjacent populations. To explore the potential mechanisms underlying this regulatory divergence, we searched for transcription factors (TFs) that might bind to these regions. We performed TF binding site enrichment analysis on these ATAC-seq peaks and identified several potentially important TFs (Fig. 2B). The gene *Mothers against dpp* (*Mad*) is related to wing development in *Heliconius melpomene* (*24*). *Mad* was also previously identified as a possible gene involved in chromatin divergence between *H. erato, Heliconius charithonia*, and *H. melpomene* (*25*). Several more TFs may be relevant for our study, including *sine oculis* (*so*), crucial for the formation of compound eyes in *Drosophila* (*26*). Furthermore, *Suppressor of Hairless* [*Su(H)*] operates within the Notch signaling pathway, which is fundamental for regulating cell differentiation, proliferation, and apoptosis during wing development in *Drosophila* (*27*). A notable gene includes *pangolin* (*pan*), involved in segment polarity through the *Wnt* signaling pathway (*28*). It has been previously found in *H. erato* and *H. melpomene* as a possible gene involved in wing development (*29*). Because these genes are active at different stages of development, they may contribute to generating a cascade effect that would explain the genome-wide chromatin activity divergence between populations.

We identified the nearest gene to each population-specific ATAC-seq peak as a proxy for potential regulatory targets, revealing candidate loci that may be involved in divergent evolution (table S6 and Table 1). Across the *H. e. demophoon*–*H. e. hydara* hybrid zone, 344 genes were identified, while 237 genes were associated with population-specific peaks in the *H. e. etylus*–*H. e. notabilis* comparison. In total, 581 genes were annotated across both datasets, of which 40 exhibit functions plausibly linked to divergent evolution in *Heliconius*.

**Table 1.** Relevant genes under ATAC-seq or *F_st_* signal. Table reporting identified relevant genes, general traits they participate in, gene name, a short description of their functions, and hybrid zone they have been identified with (Panama: *H. e. demophoon* × *H. e. hydara*; Ecuador: *H. e. notabilis* × *H. e. etylus*). Footnotes indicate the type of evidence by which each gene was identified.

	Panama—*H. e. demophoon* × *H. e. hydara*	Ecuador—*H. e. notabilis* × *H. e. etylus*
Trait	Gene name and function	Gene name and function
Circadian rhythm and behavioral timing	^*^ Protein timeless homolog—Regulates circadian rhythms, which can affect reproductive timing	^*^ Protein doublesex—Sex-specific gene influencing behavioral differences
Cuticle formation and pigmentation	^*^ Phenoloxidase subunit 1—Involved in melanin biosynthesis, pigmentation, and cuticle hardening	^*^ Phenoloxidase subunit 2-like—Important for melanin biosynthesis and cuticle sclerotization
	^*^ l-Ascorbate oxidase-like—Potential role in oxidative stress response related to pigmentation	^*^ Fatty acyl-CoA reductase 1-like—Involved in cuticle and pheromone biosynthesis
		^*^ Fatty acid synthase-like—Cuticle waterproofing and pheromone production
	^†^ Phenoloxidase-activating factor 2-like—Melanin biosynthesis, pigmentation, immunity	^†^ l-Dopachrome tautomerase yellow-f-like—Possible involvement in pigmentation pathways
	^†^ Chitin synthase chs-2-like—Involved in cuticle formation	^†^ Laccase-4-like—Involved in cuticle sclerotization and pigmentation
	^†^ Cuticular protein glycine-rich 20—Likely contributes to cuticle structure	^†^ Endocuticle structural glycoprotein SgAbd-3-like—Structural component of the insect cuticle
	^†^ Chitin deacetylase 5—Involved in chitin modification	^†^ Protein goliath—May regulate pigmentation or cuticle formation
	^†^ Protein white—Eye pigment transport	^†^ Cuticular protein RR-1 motif 47 precursor—Structural component of the exoskeleton
Developmental patterning and morphogenesis	^*^ Craniofacial development protein 2-like—Likely involved in morphological development	^*^ WntA signaling ligand—Involved in developmental patterning and morphological divergence
	^*^ Rootletin—Structural protein, potentially important for cilia formation and cell organization	^*^ Craniofacial development protein 2-like—Influences morphological traits
	^*^ Cortex locus—Wing patterning	^*^ Optix—Eye development, wing patterning
	^†^ Bone morphogenetic protein receptor type-1B—Essential for developmental signaling	^†^ Protein Spire—Regulates actin cytoskeleton organization, important for intracellular transport and cell division
	^†^ Disheveled-associated activator of morphogenesis 1—Key in Wnt signaling pathway for morphogenesis	^†^ Protein extra-macrochaetae—A TF involved in developmental patterning
	^†^ Trithorax group protein osa—Chromatin remodeling, developmental activation	^†^ Retinal Homeobox Protein Rx1-like—Eye development, retinal progenitor cells, and photoreceptor differentiation
	^†^ Protein crumbs—Important for cell polarity and morphogenesis	^†^ Protein Vein—Functions as an EGFR ligand, involved in cell proliferation, differentiation, and developmental signaling
	^†^ PAX-interacting protein 1-like—Plays a role in developmental patterning	^†^ Protein Vestigial—Regulates wing development and contributes to tissue growth and organogenesis
	^†^ Neurogenic locus protein delta—Notch signaling and cell differentiation	^†^ Helix-loop-helix protein delilah-like—Plays a role in muscle and neural development
		^†^ Fat-like cadherin-related tumor suppressor homolog—Cell adhesion and morphogenesis
		^†^ Zinc Finger Protein Rotund-like—TF regulating limb and sensory organ development, morphogenesis
		^†^ Neural Cell Adhesion Molecule 1-like (NCAM1-like)—Neural development, synaptic plasticity, and neuron-neuron interactions
		^†^ Neurogenic locus protein delta—Involved in Notch signaling, important for tissue differentiation
Hormone metabolism and detoxification	^*^ Farnesol dehydrogenase-like—Juvenile hormone metabolism, development and reproduction	^*^ Fatty acid synthase-like—Involved in lipid metabolism, which can influence pheromone production
	^*^ Ecdysteroid 22-kinase—Key enzyme in molting hormone regulation	^*^ Epoxide hydrolase 4-like—Involved in detoxification and chemical adaptation
	^*^ Fatty acid synthase-like—Affects pheromone production and metabolism	^*^ Isopentenyl-diphosphate Delta-isomerase 1—Essential for hormone biosynthesis, including juvenile hormone
	^*^ Multidrug resistance protein homolog 49-like—Potential role in detoxification and chemical resistance	
	^*^ Carboxylesterase 1C—Detoxification enzyme, possibly affecting ecological adaptation	
	^†^ Cytochrome P450 6B6-like—Involved in hormone metabolism and detoxification	^†^ Fatty acid synthase-like—Related to pheromone biosynthesis, influencing mate recognition
	^†^ Estradiol 17-beta-dehydrogenase 11-like—Involved in steroid hormone metabolism	^†^ Probable nuclear hormone receptor HR3—Regulates molting and development
	^†^ Very-long-chain 3-oxoacyl-CoA reductase—Lipid metabolism, which can affect hormone synthesis	^†^ Retinol dehydrogenase 11-like—Involved in hormone metabolism, possibly linked to reproductive signaling
		^†^ Basic juvenile hormone-suppressible protein 1-like—Could influence development and reproductive maturation
		^†^ Ethanolamine-phosphate cytidylyltransferase—Phospholipid metabolism, which may influence hormone signaling
Neurotransmission and sensory perception	^*^ Neuropeptide CCHamide-1 receptor—Involved in feeding and behavioral responses	^*^ Neuropeptide CCHamide-1 receptor—Regulates feeding, mating, and behavioral responses
	^*^ Adenosine receptor A2b—Neurotransmission and stress response	^*^ Piezo-type mechanosensitive ion channel component—Potential role in mechanosensory perception
	^*^ Tachykinins-like—Neuropeptides affecting sensory processing and mating behavior	^*^ Adhesion G protein-coupled receptor A3—Involved in sensory signaling and behavior
	Gustatory receptor for sugar taste 64f-like—Chemosensory perception, host plant preference	^*^ Nose resistant to fluoxetine protein 6-like—Possible role in neural responses and behavior
	Dscam2—Neuronal wiring, potentially affecting mate recognition	
	^*^ Dendritic arbor reduction protein 1-like—Neural development and plasticity	
	^*^ Protein unc-13 homolog C-like—Synaptic transmission and neurotransmitter release	
	^†^ Sodium channel protein Nach-like—Involved in neural signal transmission	^†^ Neurexin-4—Involved in synapse formation and function, affecting mating behavior
	^†^ Neuronal acetylcholine receptor subunit alpha-3—Critical for neurotransmission	^†^ Muscarinic acetylcholine receptor DM1—Involved in neural signaling and sensory processing
	^†^ Synaptic vesicle glycoprotein 2B-like—Functions in neurotransmitter release	^†^ Acetylcholine receptor subunit beta-like 1—Neuromuscular signaling and synaptic transmission
	^†^ Potassium channel AKT3-like—Regulates neuronal excitability	^†^ General odorant-binding protein 1-like—Important for chemical communication in mate recognition
	^†^ Ras-related protein Rap1—Plays a role in synaptic plasticity	^†^ Nose resistant to fluoxetine protein 6-like—Involved in neurotransmission and behavior
	^†^ Neurexin-4—Important in synaptic adhesion and neural connectivity	^†^ Synaptotagmin-like protein 4—Regulates neurotransmitter release, influencing behavior
	^†^ Opsin—Photoreceptor protein, crucial for sensory perception	^†^ Caskin-2—Regulates synaptic organization
		^†^ Innexin inx7—Affects electrical synapses, potentially altering mating signals
		^†^ Neural cell adhesion molecule 1-like—Plays a role in neural connectivity and behavior
		^†^ Toll-like receptor Tollo—Possibly involved in immune signaling that affects sensory perception
Reproductive and postmating response	^*^ Sex peptide receptor-like—Regulates postmating responses in females	^*^ Protein doublesex—Controls sex-specific reproductive traits
	^*^ Seminal fluid protein HACP057—Affects sperm viability and female postmating behavior	^*^ Peptidoglycan recognition protein 1-like—Immune regulation, potentially affecting reproductive compatibility

* Genes identified through genomic differentiation analyses based on *F_st_*.

† Genes identified through ATAC-seq analyses.

Because sequence expansion in the pangenome can alter physical distances between genomic features, we evaluated its effect on peak-gene assignments by comparing nearest-gene annotations derived from the pangenome with those obtained using the original *H. e. demophoon* reference. We observed 94% concordance between references, with a strong correlation among the concordant matches (Pearson = 0.98; Spearman = 0.99; fig. S7); for the small fraction of discordant cases, assignments based on the original reference genome were retained.

### Correlation between chromatin differences and *F_st_* across levels of isolation

We hypothesized that population-specific differences in chromatin accessibility may reflect, or potentially precede, genetic divergence. Under this hypothesis, we expected elevated *F_st_* or selective sweep signals in open chromatin regions identified by ATAC-seq. To test for an association between chromatin accessibility and genetic differentiation, we applied two complementary approaches: a Kolmogorov-Smirnov (KS) test comparing the distribution of *F_st_* values within unique ATAC-seq peaks to genomic background, and a custom binomial test assessing whether unique ATAC-seq peaks are overrepresented in specific *F_st_* intervals.

**Fig. 4.**
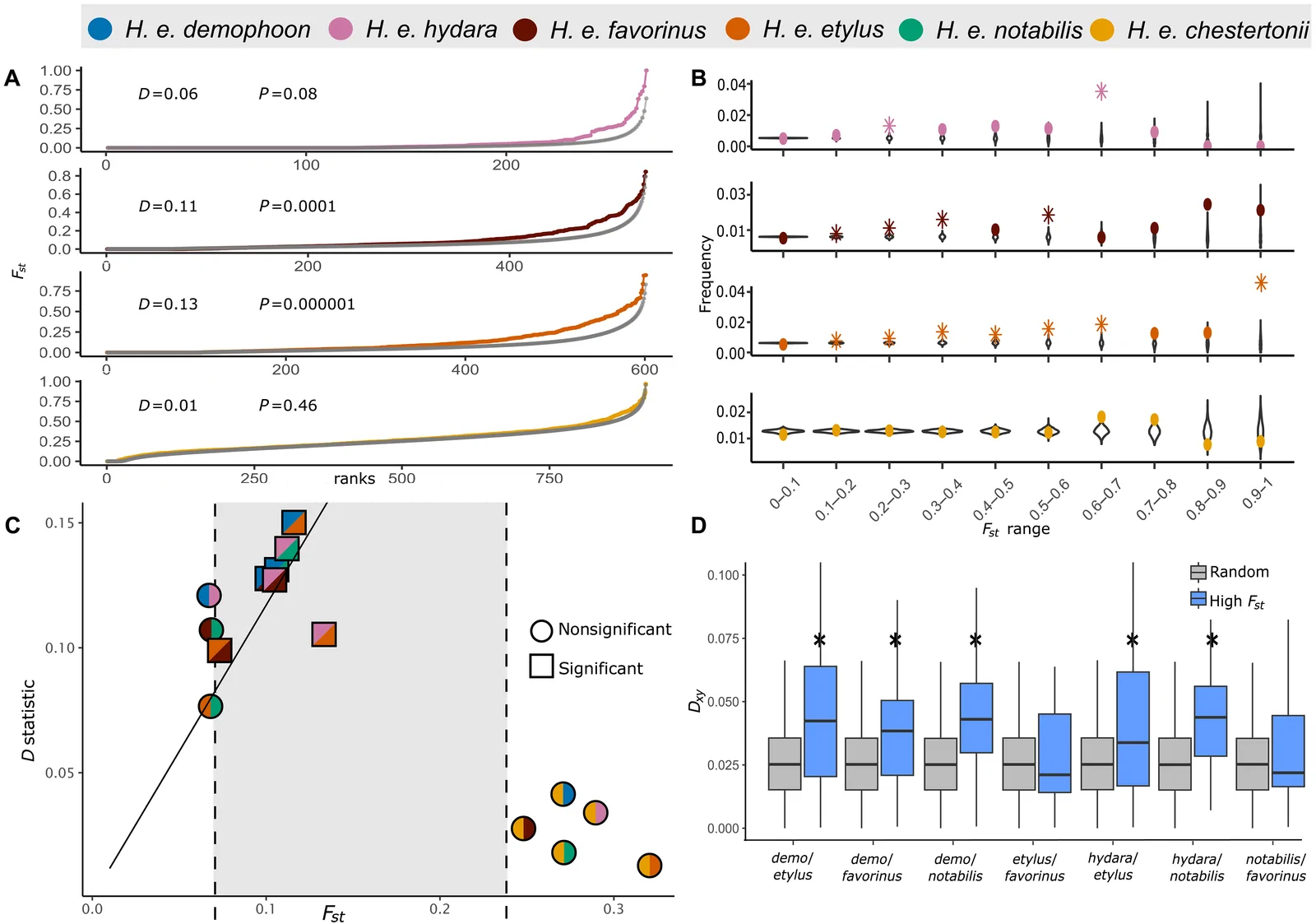
Distance-related patterns and nonhomologous sequence dynamics. (**A**) Results from the KS test for the same comparisons. The *D* statistic from the KS test is reported along with its adjusted *P* value. (**B**) Results from the custom binomial test for the same pairwise comparisons. Statistically significant results are indicated by asterisks. (**C**) Scatterplot summarizing the results of KS tests for all 15 possible pairwise comparisons. The *y* axis represents the *D* statistic from the KS test, while the *x* axis indicates the average *F_st_* value for each comparison. Statistically significant tests (adjusted *P* < 0.05) are represented by squares, with different colors indicating the populations being compared. (**D**) Boxplot showing *D_xy_* comparisons between unique peaks with high *F_st_* values and random points for each of the significant pairs identified in (A). Asterisks indicate the significance of the KS test. Random samples were generated as the ranked average of 1000 points for each comparison.

The KS test detected no significant shift in *F_st_* distributions in either hybrid zone (*H. e. demophoon*–*H. e. hydara*: *D* = 0.06, adjusted *P* = 0.2; *H. e. notabilis*–*H. e. etylus*: *D* = 0.07, adjusted *P* = 1; Fig. 4A and table S7). In contrast, the binomial test identified a small number of significant *F_st_* intervals. In the *demophoon-hydara* comparison, unique ATAC-seq peaks were enriched in the 0.2 to 0.3 and 0.6 to 0.7 *F_st_* bins (Fig. 4B), while in the *notabilis-etylus* comparison, only a single interval (0.4 to 0.5) showed significance (table S7).

Together, these results indicate a weak and inconsistent association between chromatin accessibility divergence and elevated *F_st_* across hybrid zones, with limited support depending on the statistical framework used. Applying the same analyses to selective sweep signals across all adjacent population pairs yielded no significant results (fig. S8); therefore, selective sweep analyses were not pursued further.

To test whether associations between chromatin accessibility and genetic differentiation become more apparent under reduced gene flow, we extended the *F_st_*–ATAC-seq analyses to non–hybrid zone population pairs using *H. e. demophoon* as a reference (*H. e. demophoon* versus *H. e. favorinus*, *H. e. etylus*, and *H. e. chestertonii*). The KS test revealed significant shifts in *F_st_* distributions for the *demophoon-favorinus* (*D* = 0.11, *P* < 0.01) and *demophoon-etylus* (*D* = 0.13, *P* < 0.01) comparisons, but not for *demophoon-chestertonii* (*D* = 0.01, *P* = 0.46; Fig. 4A). KS effect sizes increased with population divergence up to the *demophoon-etylus* comparison (Fig. 4A and table S7).

Consistent with this pattern, the binomial test identified multiple significant *F_st_* intervals for *demophoon-favorinus* (four intervals) and *demophoon-etylus* (seven intervals), whereas no significant intervals were detected for *demophoon-chestertonii* (Fig. 4B). Repeating the binomial analysis using *F_st_* calculated in 50-kb windows yielded comparable results, with the exception of the *demophoon-chestertonii* comparison, where three *F_st_* intervals (0.4 to 0.5, 0.5 to 0.6, and 0.7 to 0.8) reached significance (fig. S9).

Together, these results indicate that associations between chromatin accessibility and elevated *F_st_* are weak in closely connected populations but become more detectable as gene flow is reduced, increasing with geographic and genetic distance.

To assess whether the observed association between chromatin accessibility and genetic differentiation was consistent across the dataset, we extended the KS analysis to all pairwise population comparisons (15 total pairs). For each comparison, we extracted the KS *D* statistic, quantifying the shift in *F_st_* values within unique ATAC-seq peaks relative to genomic background, and the corresponding adjusted *P* value, and plotted these against the mean genome-wide *F_st_* (Fig. 4C). The KS test was significant within an intermediate range of divergence (mean *F_st_* ≈ 0.07 to 0.24), within which *D* values increased proportionally with average *F_st_* (*F* = 158.1, *P* = 5.6 × 10^−5^; Fig. 4C). Comparisons involving *H. e. chestertonii* were consistently nonsignificant, likely reflecting elevated background divergence.

To evaluate the robustness of this pattern, we repeated the analysis using alternative datasets, including *F_st_* calculated in 50-kb windows instead of 1-kb windows and a relaxed definition of unique ATAC-seq peaks requiring presence in two out of three samples. These additional analyses were performed to exclude potential effects of window size and sample number. Across all cases, results were qualitatively consistent and reproduced the same overall trends although with less strong *P* values (fig. S10).

In population pairs that do not currently hybridize, genetic differentiation at ATAC-seq peaks is positively associated with increasing genome-wide *F_st_*, a pattern observed across all pairwise comparisons except those involving *H. e. chestertonii*. To distinguish whether this signal reflects strong local selection or reduced gene flow, we examined the relationship between *F_st_* and absolute divergence (*D_xy_*) within these regions. For each significant pairwise comparison identified in Fig. 4A, we extracted high-*F_st_* values (95th percentile) from unique ATAC-seq peaks and compared their *D_xy_* values to background expectations derived from 1000 ranked random samples. In five of the seven comparisons, *D_xy_* values within high-*F_st_* ATAC-seq peaks were significantly elevated relative to background (KS test; Fig. 4D), consistent with these regions acting as barriers to gene flow rather than reflecting isolated selective sweeps.

Overlap between *F_st_*-enriched ATAC-seq peaks and genome-wide candidate regions identified by the *F_st_* screening framework was minimal. Using a ≥1-bp overlap criterion, only 5 out of 385 differentiated ATAC-seq peaks (∼1%) overlapped candidate *F_st_* regions in the *demophoon-hydara* comparison, while no overlap was detected in the other hybrid zone (0 out of 380 peaks).

The limited overlap indicates that most chromatin accessibility differences occur outside broad regions of elevated sequence differentiation. This pattern is consistent with the distinct genomic scales targeted by the two approaches: *F_st_* captures extended regional clustering of sequence divergence, whereas ATAC-seq identifies highly localized regulatory changes. Together, these results support the interpretation that regulatory divergence detected by ATAC-seq is largely independent of extended *F_st_* signals and that the two analyses capture complementary aspects of genomic differentiation during early stages of divergence.

### Simulation results

Our results indicate that *F_st_* measured at population-specific ATAC-seq peaks can serve as an indicator of regulatory barrier loci, but its informativeness depends on the demographic context in which selection and gene flow interact. To evaluate this dependency, we performed simulations under varying levels of migration between two populations (fig. S11). When gene flow is high (*m* = 0.1), regions under weak selection and neutral regions exhibit indistinguishable *F_st_* distributions and converge to a shared equilibrium at very low differentiation. As gene flow decreases, selected regions transiently diverge from the genomic background, producing elevated *F_st_* relative to neutral regions. After enough generations, differentiation accumulates genome-wide over time, and *F_st_* values for selected and neutral regions ultimately converge again.

Together, these simulations indicate that *F_st_*-based detection of barrier loci is most informative at intermediate levels of divergence, whereas both high gene flow and advanced divergence reduce contrast relative to background differentiation. This theoretical framework closely matches the empirical patterns observed in our data. While 1-kb windows effectively capture *F_st_* enrichment at ATAC-seq peaks across most allopatric population pairs, comparisons involving *H. e. chestertonii*, the most divergent lineage within the *H. erato* complex, consistently lack detectable enrichment (Figs. 3 and 4). *H. e. chestertonii* exhibits not only wing pattern–based assortative mating but also evidence of hybrid unviability, consistent with elevated genome-wide divergence (*8*). Accordingly, *F_st_* distributions in comparisons involving *H. e. chestertonii* display broader variance around the mean rather than a sharp central peak with limited high-divergence outliers (fig. S12), reducing contrast between chromatin-associated loci and the genomic background. Thus, the absence of *F_st_* enrichment at ATAC-seq peaks in these comparisons is consistent with expectations under advanced divergence rather than indicating an absence of regulatory barrier loci.

### Targets of selection for incipient reproductive isolation in *H. erato*

Genomic research on *Heliconius* butterflies has long focused on wing color pattern genes, yet complete reproductive isolation in this genus has been shown to extend beyond coloration (*30*). A broader suite of traits, including neural pathways, chemical communication, and sensory perception, plays a crucial role in ecological adaptation and speciation (*7*, *31*–*33*). Consistent with this view, by integrating genome-wide patterns of genetic differentiation with population-specific chromatin accessibility, our analyses reveal a complex signature of divergence that likely reflects incipient reproductive isolation acting across multiple functional axes (Table 1).

Divergence in circadian regulation and behavioral timing likely plays a key role in structuring reproductive schedules, with genes linked to circadian clocks and sex-specific expression influencing the timing of mating activity and courtship displays (*34*, *35*). These shifts can reduce hybridization by reinforcing temporal or behavioral isolation mechanisms. Alongside behavioral rhythms, differences in cuticle formation and pigment biosynthesis highlight another axis of divergence. Genes involved in melanin production, chitin remodeling, and cuticle architecture suggest modifications in pigmentation, waterproofing, and even pheromone dispersion (*18*). Such traits influence both environmental adaptation through desiccation resistance or camouflage and mate recognition, where subtle changes in wing coloration or texture may serve as visual mating cues and affect ecological performance.

Morphological divergence emerges as another important trait, driven by changes in genes that regulate developmental patterning and tissue morphogenesis. Variation in components of Wnt, Notch, and epidermal growth factor receptor (EGFR) signaling pathways, along with chromatin modifiers and cytoskeletal regulators, points to differences in body plan specification and organ formation. These developmental changes may underlie subtle yet crucial differences in wing shape, sensory organ development, traits that can influence mate choice, and habitat adaptation. This morphological variation is paralleled by divergence in neural signaling and sensory perception (*31*–*33*). Our results show differentiation in genes tied to synaptic transmission, chemosensory reception, and photoreception, thus suggesting population-specific tuning of sensory systems. Such modifications can alter how individuals perceive visual and chemical stimuli in their environment (*36*), influencing both mate recognition and host plant selection. These sensory shifts are likely to contribute strongly to premating isolation and ecological specialization.

At the physiological level, we also observe divergence in hormone metabolism and detoxification pathways. Genes involved in juvenile hormone regulation, steroid biosynthesis, and xenobiotic detoxification reflect changes in developmental timing, pheromone production, and adaptation to local environmental toxins (e.g., host plant chemistry). These physiological shifts may synchronize development with local ecological conditions, reinforce reproductive timing, or influence female receptivity, all critical components in maintaining population boundaries (*37*, *38*).

Last, divergence in reproductive and postmating processes further strengthens isolation. Genes associated with seminal fluid composition, female postmating responses, and reproductive immunity suggest that barriers to gene flow can persist beyond courtship and copulation. These molecular incompatibilities may reduce fertilization success, bias sperm competition, or modulate female remating behavior, contributing to reproductive isolation even when mating occurs.

The functional axes discussed below correspond to six functional categories: Circadian Rhythm and Behavioral Timing; Cuticle Formation and Pigmentation; Developmental Patterning and Morphogenesis; Hormone Metabolism and Detoxification; Neurotransmission and Sensory Perception; and Reproductive and Postmating Response (Table 1). These categories were selected a priori based on biological traits repeatedly implicated in divergence and reproductive isolation in *Heliconius* and other Lepidoptera, and are intended as a hypothesis-generating framework rather than as functionally validated categories.

### Evolutionary history of SVs

**Fig. 5.**
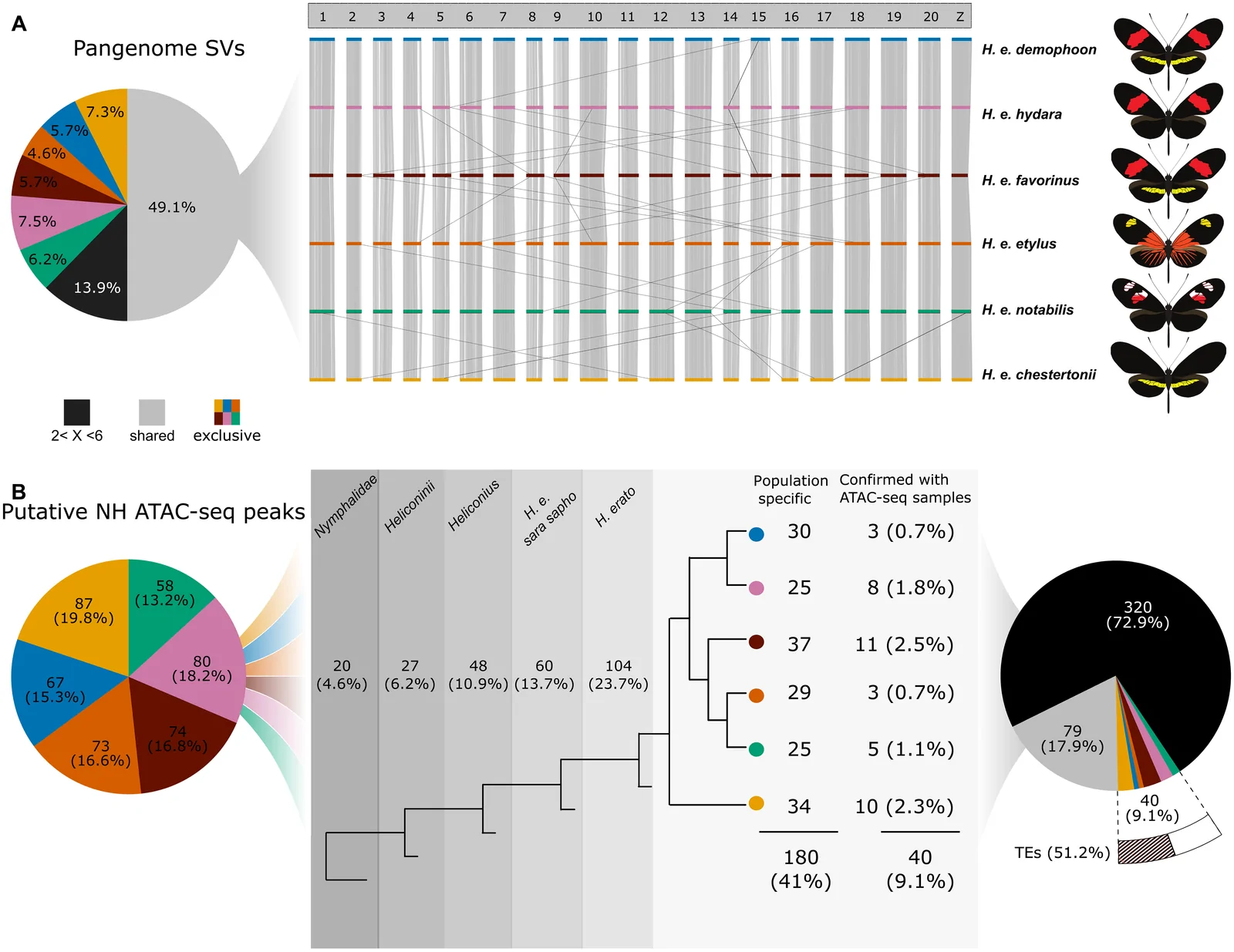
Structural and regulatory divergence among populations. (**A**) Genome-wide synteny plot of shared sequences, with each line corresponding to a different population. Gray lines represent sequences longer than 5 kb. Only transpositions confirmed with genome reads, as described in Materials and Methods, are shown. (**B**) Pie chart depicting the number of ATAC-seq peaks that fall within nonhomologous sequences (NH peaks) for each population. The second panel shows the distribution of these putative NH peaks at different phylogenetic levels within the Nymphalidae family. The rightmost pie chart summarizes how many putative NH peaks are confirmed as nonhomologous based on ATAC-seq data, as detailed in Materials and Methods. In addition, it reports the percentage of exclusive nonhomologous peaks covered by TEs.

Across the constructed pangenome, 49.1% of the sequence was conserved among all *H. erato* populations, forming locally collinear blocks. In contrast, 37% of the pangenome consisted of nonhomologous sequences, with population-specific proportions ranging from 10% in *H. e. etylus* to 16% in *H. e. chestertonii*. An additional 13.9% of the genome was partially conserved and shared among some, but not all, populations (Fig. 5A). The conserved regions exhibited near-complete synteny across all populations, interrupted only by 16 transpositions totaling 196,166 bp (0.03% of the pangenome), with individual events ranging from 2016 to 66,539 bp (Fig. 5A). To explore structural variation more broadly, we analyzed genome-wide correlations between SV frequency and chromosome length. SV frequency was inversely correlated with chromosome length (fig. S13), likely reflecting the effects of linked selection. Longer chromosomes typically experience lower recombination rates per base pair, intensifying selective sweeps and purifying selection, which reduces overall diversity (*39*). This pattern mirrors the distribution of single-nucleotide polymorphism (SNP) variation in *Heliconius* found in previous work (*25*).

Using a single genome per population and unique ATAC-seq peaks, we initially identified a conserved genomic core together with population-specific elements. However, incorporating multiple individuals per population and integrating ATAC-seq data in a broader phylogenetic context revealed a more conservative view of nonhomologous variation. Intersecting ATAC-seq peaks with pangenome-derived nonhomologous regions initially identified 439 putative population-specific peaks across the six populations (Fig. 5B). Phylogenetic screening showed that only 41% of these peaks were restricted to the focal populations, with the remainder detectable across other *H. erato* subspecies or more distantly related Heliconiine and Nymphalid taxa.

To further evaluate population specificity, we leveraged the higher resolution provided by multiple ATAC-seq individuals per population and reassessed these candidates by intersecting each population’s putative nonhomologous peaks with ATAC-seq profiles from the remaining populations. This analysis confirmed that only 9.1% (40 peaks) met the criteria for population-specific nonhomology, demonstrating that analyses based on single genomes substantially overestimate nonhomologous content by failing to capture within-population variation.

The 40 putative population-specific ATAC-seq peaks were enriched for repetitive sequence, with an average of 51.2% of base pairs overlapping annotated transposable elements (TEs; Fig. 5B). Consistent with the importance of population-level sampling, reclassification of the original 439 candidate peaks using resequencing data from 5 to 10 individuals per population confirmed that most represent shared or polymorphic variation rather than fixed population-specific sequence. Under this framework, 75% of peaks were shared across populations, 23% were variable, fewer than 1% were uninformative, and only 0.5% were exclusive to a single population. Notably, all population-exclusive peaks were detected in *H. e. chestertonii*, the most genetically divergent population analyzed, consistent with expectations based on its elevated genome-wide differentiation (fig. S14).

Together, these analyses demonstrate that apparent population-specific structural and regulatory variation inferred from single-reference genomes is substantially overestimated. By integrating population-level sampling, phylogenetic context, chromatin accessibility, and resequencing-based validation, we show that most putative nonhomologous ATAC-seq peaks reflect shared or polymorphic variation rather than fixed population-specific sequence. These findings highlight the importance of multi-individual and multilayered approaches for accurately characterizing structural and regulatory variation during incipient divergence.

## DISCUSSION

The genetic basis of early reproductive isolation remains a central question in evolutionary biology: Is isolation driven by a few major-effect loci or by numerous loci with varying effect sizes? We address this by analyzing *H. erato*, a butterfly species with populations at different stages of divergence across Central and South America. Earlier genomic studies (*15*) identified only a few genomic regions, primarily wing patterning genes, with elevated *F_st_*, supporting the hypothesis that a small number of major-effect loci account for most genome-wide patterns of divergence. These color pattern genes not only directly affect a butterfly’s survival but also reinforce mate choice (*40*–*42*), making them potential targets for early reproductive isolation. However, when *H. erato* populations were compared with the more geographically isolated *Heliconius himera* (*43*, *44*), and *H. e. chestertonii* (*45*), genome-wide divergence increased to the point that *F_st_* peaks near known color pattern loci became undetectable (*8*). These results suggest that genome-wide patterns of divergence appear abruptly rather than gradually and, importantly, may remain undetectable in the presence of gene flow.

To revisit this hypothesis, we constructed an *H. erato* pangenome using six reference genomes from populations spanning a range of geographic distances and levels of gene flow (Fig. 1, A and B). We reanalyzed resequencing published data (*15*), focusing on hybrid zones in Panama (*H. e. hydara* × *H. e. demophoon*) and Ecuador (*H. e. notabilis* × *H. e. etylus*) (Fig. 2). Contrary to approaches of earlier studies, which identified only a few divergence peaks, our analysis revealed an elevated genome-wide *F_st_* divergence (Fig. 2). These additional divergent regions point to potential targets of selection beyond wing patterning, which may be functionally tied to behavioral, ecological, or physiological adaptations (Table 1 and tables S3 and S6). Many of these regions also show elevated *D_xy_*, consistent with the presence of barrier loci (*19*, *46*).

This improved resolution to characterize patterns of genomic divergence in the early stages of population differentiation stems from enhanced mapping using population-specific pangenome assemblies and a finer-scale analysis based on 1-kb *F_st_* windows (fig. S5). Previous studies (*15*) relied on a single *H. e. demophoon* reference genome and used 50-kb sliding windows, which likely masked smaller signals of selection. Recent work has highlighted that selection, gene flow, and recombination heterogeneity demand higher-resolution approaches (*47*, *48*).

A multilocus structure of divergence is also evident at the epigenetic level (Fig. 3). ATAC-seq profiling of wing tissue reveals widespread population-specific differences in chromatin accessibility, indicating regulatory divergence extending well beyond canonical wing patterning genes (Figs. 2 and 3A). These regulatory differences likely affect multiple wing-associated traits, including morphology, pigmentation, pheromone production, and sensory function (table S6). Consistent with this view, TF motif enrichment analyses reveal divergence in regulatory binding landscapes, suggesting broader shifts in regulatory networks rather than changes confined to individual loci (Fig. 2B).

Although chromatin accessibility divergence is not tightly coupled to local *F_st_* across hybrid zones (Fig. 4, A and B), population pairs with reduced gene flow show increasing concordance between population-specific ATAC-seq peaks and regions of elevated *F_st_* and *D_xy_*. This pattern is consistent with regions resisting introgression under migration-selection balance (*19*, *49*) and suggests that regulatory divergence can contribute to barriers to gene flow. Together, these results support a model in which epigenetic and regulatory divergence forms part of a multilocus barrier architecture, complementing sequence-level differentiation during the early stages of speciation.

Previous work in *Heliconius* strongly supports a multilocus and regulatory basis of population divergence rather than simple single-locus effects. Genome-wide analyses have shown that divergence follows a highly polygenic architecture, particularly in low-recombination regions that resist introgression (*13*), and hybrid incompatibilities in butterflies are consistent with a multilocus model, with many small-effect loci accumulating, especially on the Z chromosome (*50*). Early population genetic studies further demonstrated that canonical wing patterning loci are not isolated mutations, but extended haplotypes composed of multiple linked sites under selection, forming genomic “hotspots” of divergence maintained over long evolutionary timescales (*24*). More recent quantitative trait mapping confirmed that even subtle variation in wing pattern shape and size is controlled by numerous small-effect loci, with limited genetic parallelism beyond major patterning genes such as *optix* (*51*). In parallel, population-level epigenomic analyses revealed widespread divergence in chromatin accessibility among *H. erato* populations, with regulatory elements enriched in regions of elevated *F_st_*, directly linking regulatory variation to population structure and local adaptation (*52*). Building on this, another work (*53*) showed that adaptive divergence of red wing patterns is maintained through selection acting on a network of *optix*-bound regulatory elements and downstream target genes, which form physically and functionally connected adaptive hubs that collectively contribute to genomic differentiation across hybrid zones. Together, these lines of evidence support interpreting population-specific ATAC-seq signals and elevated differentiation as components of a broader, multilocus architecture underlying incipient reproductive isolation in *Heliconius*. In this framework, barriers to gene flow emerge incrementally through the cumulative effects of many small- to moderate-effect changes across regulatory, behavioral, physiological, and reproductive pathways, rather than from a single dominant isolating mechanism.

Structural variation is a major driver of genome evolution and speciation (*54*), yet single-genome-per-population approaches capture only a limited fraction of this diversity. Consistent with this, our chromatin accessibility analyses indicate that many population-specific ATAC-seq peaks are polymorphic within populations, revealing substantial standing structural variation. Of the putative nonhomologous regulatory elements identified from the pangenome, only ∼9% pass the filter after incorporating multiple individuals from ATAC-seq data, and fewer than 1% remain supported when evaluated across all resequenced samples (fig. S14). In addition, several of these elements are also detected in other Nymphalidae species, suggesting that a portion of the apparent nonhomology reflects the retention of ancestral variation rather than recent population-specific innovation.

These results highlight an emerging issue: Accurately characterizing population-specific structural and regulatory variation requires sampling a sufficient number of individuals to capture within-population polymorphism. Limited sampling can lead to overestimation of population specificity and obscure the distinction between ancestral and derived variants. This phenomenon, commonly referred to as reference bias (*55*), has been extensively characterized in the context of human disease (*56*). However, our results demonstrate that it represents a pervasive issue in studies of population variation. Nonetheless, our analyses deliberately focus on a highly conservative and reproducible set of chromatin accessibility differences, and the central pattern described in this study remains robust to these limitations. While the exact number of nonhomologous regulatory elements should therefore be interpreted as an approximation, the relationship between chromatin accessibility divergence and genomic differentiation that we document is well supported across datasets and analytical scales. Future work should prioritize expanded population sampling and multi-individual genome assemblies to refine quantitative estimates and further resolve the contribution of structural variation to regulatory divergence and reproductive isolation.

Our findings challenge the long-standing view that genomic divergence in populations experiencing high gene flow is driven primarily by a small number of major-effect loci. Instead, we demonstrate that incipient reproductive isolation emerges from a multilocus and genome-wide architecture, integrating sequence divergence, population-specific chromatin accessibility, and structural variation. The observed patterns extend the genomic basis of divergence in *H. erato* beyond traits traditionally emphasized in this system, revealing coordinated differentiation across regulatory, behavioral, physiological, and ecological dimensions (*31*–*33*). The persistence of ancestral SVs further highlights the dynamic and heterogeneous nature of the *H. erato* genome, shaped by both historical contingency and contemporary selective forces (*57*). Together, these results reinforce the role of polygenic adaptive divergence in initiating reproductive barriers under substantial gene flow (*50*), providing a mechanistic foundation for the earliest stages of speciation and helping to explain the extraordinary diversity of the *Heliconius* radiation.

## MATERIALS AND METHODS

The data generation and analyses described in Materials and Methods below were originally conducted as part of the thesis *Decoding Diversity: The Role of Structural Variants, Gene Regulatory Networks, and Chromatin Dynamics in Shaping the Evolution of Heliconius Butterflies* (*58*).

### Genome assemblies

Genomes were generated for five *H. erato* populations (*H. e. notabilis*, *H. e. etylus*, *H. e. hydara*, *H. e. chestertonii*, and *H. e. favorinus*). Although some sources classify *H. e. chestertonii* as a distinct species (*32*, *59*), the majority, particularly those focused on speciation, support its classification as an instance of incipient speciation (*60*). Consequently, in this study, *H. e. chestertonii* is considered a geographic subspecies of *H. erato*. For each genome, we extracted high–molecular weight DNA from flash-frozen pupas. Library preparation using 10x Chromium technology for linked reads (10x Genomics, San Francisco, USA) and Illumina sequencing was carried out by Novogene Co., Ltd., with a target coverage of 100×. We assembled the linked-read sequencing data using the Supernova 2.1.1 assembler (*61*) using the default recommended settings and a maximum number of reads of 200 million. Raw assembly outputs were transformed to fasta format using the pseudohap2 option to generate two parallel pseudo-haplotypes from the diploid genome. Quality control of the genomes was performed using genome-wide statistics calculated on the phase blocks. Synteny with the *H. e. demophoon* V1 genome was performed using Tigmint v1.2.3 (*62*). Last, Benchmarking Universal Single-Copy Orthologs (BUSCO) was used to assess genome assembly and annotation completeness (*63*).

### Pangenome

Using seq-seq-pan (*64*), we generated an *H. erato* population pangenome aligning a total of six *H. erato* populations: *H. e. demophoon*, *H. e. hydara*, *H. e. notabilis*, *H. e. etylus*, *H. e. favorinus*, and *H. e. chestertonii*. The pangenome is a collection of core and variable genomic regions derived from multigenome alignments, and it can be used as a reference for mapping next-generation sequencing (NGS) data. The *H. e. demophoon* V1 reference genome was used as the first genome in the genome list to ensure that the resulting pangenome alignment was ordered correctly. The *H. e. demophoon* chromosome 21 final coordinates on the pangenome is 688,640,245; sequences after that position were not used to avoid spurious feature mappings. This approach preserves local collinearity with the original reference (*H. e. demophoon*) while expanding the reference space to include sequences absent from a single-genome assembly, generating an extended consensus of the *H. e. demophoon* original genome.

To obtain lineage-specific sequences, we first recorded the sequence coordinates of each genome relative to the pangenome as in (*25*). We then subtracted a merged library of sequence coordinates of all other genomes from these coordinates using BEDTools v2.27.1, obtaining only the sequences exclusive to each genome (*65*).

### Resequencing

Resequencing data were obtained from (*15*). The dataset includes individuals with divergent *H. erato* mimetic subspecies populations that are found closely adjacent and sometimes connected by hybrid zones. To analyze the genomic data, the existing 100-bp paired-end Illumina resequencing data were mapped to the consensus pangenome reference genome using BWA v0.7.13 (*66*) with default parameters. We removed polymerase chain reaction (PCR) duplicates using Picard v1.1 (https://broadinstitute.github.io/picard/) and sorted the data using SAMtools (*67*). Genotypes were called using the Genome Analysis Toolkit (GATK) Haplotypecaller (*68*) with default parameters. For joint genotyping, we used GATK’s genotypeGVCFs with default parameters. We considered a minimum expected heterozygosity of 0.025 to match the populations’ high heterozygosity and grouped individuals based on subpopulation and sampling location. In the downstream analysis, genotype calls were evaluated based on the following criteria: quality (QUAL) ≥ 30, minimum depth ≥ 10, maximum depth ≤ 100 (to avoid false SNPs in repetitive regions), overall depth ≤ 100 times the number of samples, strand bias (FS) < 200, quality by depth ≥ 5, and genotype quality (GQ) ≥ 30 for variant calls.

Resequencing reads from all populations were mapped both to the original *H. e. demophoon* reference genome and to the pangenome to assess potential mapping biases introduced by the expanded reference. Mapping statistics, including the proportion of mapped reads and mismatch/error rates, were quantified for multiple *H. e. demophoon* samples and one representative sample per nondemophoon population. These metrics were compared across reference types to evaluate alignment performance.

#### Population genomics analysis

Genome-wide selective sweep signatures were identified using SweepFinder2 (*69*). A custom Python script was used to generate allele counts for biallelic SNPs, which were polarized with *Heliconius hermathena*. The default settings were used, with 1000-bp window size. *F_st_* and *D_xy_* were calculated using Simon Martin’s (popgenWindows.py; https://github.com/simonhmartin/genomics_general) using the nonoverlapping sliding windows of 1000, 5000, 10,000, and 50,000 bp. To capture the potentially small effects of regulatory elements identified by ATAC-seq (which average ∼400 bp in length), we calculated *F_st_* using 1000-bp windows. Regulatory regions can evolve rapidly and play important adaptive roles, but because they are typically small, using larger windows could miss these signals leading to an underestimation of how strongly selection has shaped genomic differences. This approach increases background noise since the small window size tends to have higher noise due to technical issues (*70*) and biological variability (*71*), which may not reflect meaningful population-wise biological differences. As a result, individual 1-kb *F_st_* windows cannot be interpreted directly as evidence of selection. To distinguish biologically meaningful signals from background noise, we implemented a density-based aggregation framework. Adjacent 1-kb *F_st_* windows were grouped into blocks of 10 consecutive windows (10 kb). For each block, we calculated (i) the number of windows with *F_st_* values exceeding the genome-wide mean (“above-average density”) and (ii) the maximum *F_st_* value observed within the block. A block was classified as a candidate differentiated signal only if it satisfied two independent criteria: (i) Its above-average density exceeded the 0.99 quantile of the genome-wide density distribution, and (ii) it contained at least one window exceeding the 0.99 percentile of the genome-wide *F_st_* distribution. Extended regions of differentiation were defined as contiguous stretches of adjacent blocks independently meeting these criteria, resulting in candidate regions spanning tens to hundreds of kilobases. This conservative framework explicitly prioritizes spatial clustering of elevated *F_st_* values over isolated extreme windows and assesses nonrandomness empirically relative to genome-wide. Because of the difference in *F_st_* distribution that can be present between the sexual chromosome and the other, this analysis was conducted separately for the Z chromosome.

We performed a permutation-based analysis to test whether *D_xy_* values differed across genomic regions defined by elevated *F_st_*. For each comparison (islands versus random, high *F_st_* versus random, and islands versus high *F_st_*), observed *D_xy_* values were compared to null expectations generated by repeated random sampling (1000 iterations) from the genome-wide background. Random samples were sorted and averaged across permutations at each rank to obtain a smoothed expected distribution, which was then contrasted with the observed data using KS tests. In parallel, a permutation framework (10,000 iterations) was used to standardize the observed mean *D_xy_* against a null distribution of sampled means, yielding *z*-scores and empirical *P* values; everything is represented in fig. S3.

### ATAC-seq library preparation

ATAC-seq libraries were prepared following the protocol described in (*52*), with some modifications. Caterpillars of each species were raised on their respective host plants until the wandering stage at the fifth instar. Live larvae were briefly placed on ice and then pinned and dissected in 1× ice-cold phosphate-buffered saline. Developing wings were dissected from fifth instar caterpillars, and the left and right forewings and left and right hindwings were pooled, respectively.

Next, the tissues were homogenized, and the nuclei were extracted by submerging them in 350 μl of sucrose solution inside 2-ml dounce homogenizers. After homogenization on ice, the solution was centrifuged to collect the nuclei. The pellet was resuspended in a cold lysis buffer to burst the cell membranes and release the nuclei. The nuclei were examined under a microscope with a counting chamber to confirm dissociation, assess their concentration, and calculate the number of nuclei (∼400,000) to be used for further processing. This number was determined based on the requirement to obtain the same amount of DNA as present in 50,000 human nuclei, which is the optimized amount for ATAC-seq (*72*).

For quality control, a 15-μl aliquot of the nuclear suspension was stained with trypan blue and imaged at 64× magnification using a hemocytometer. After confirming nuclear quality and concentration, a subsample of the nuclear volume corresponding to 400,000 nuclei was pelleted, resuspended in a transposition mix containing Tn5 enzyme (Illumina DNA Prep) in a transposition buffer, and incubated at 37°C for exactly 30 min. The resulting tagged fragments were purified using a PCR MinElute Purification Kit and amplified with custom-made Nextera primers and a NEBNext High-Fidelity 2× PCR Master Mix (New England Labs). The amplified libraries were sequenced as 37- to 76-bp paired-end fragments using NextSeq 500 Illumina technology at the Sequencing and Genomics Facility of the University of Puerto Rico.

### ATAC-seq analysis

ATAC-seq reads were trimmed with TRIMMOMATIC (*73*) using 2:30:10:2:keepBothReads LEADING:3 TRAILING:3 MINLEN:32. All the reads were mapped to the pangenome with bowtie2 (*74*). Reads with a phred score lower than 20 and nonuniquely mapped were discarded for further analysis (SAMTools,-q 20, -f 0 × 02). Duplicates were removed with Picard MarkDuplicates (http://broadinstitute.github.io/picard/). ATAC-seq peaks were called using MACS2 (*75*) with the genome size set to the pangenome size (-g 688640245) and the *q* value stricter than the default (-q 0.001). We measured ATAC-seq quality using the Fraction of Reads in Peaks (Frip) score, which is the ratio of reads mapped inside the identified ATAC-seq peak to the total number of mapped reads. We retained only those samples with a score above 0.2, which is generally acceptable (www.encodeproject.org/atac-seq/).

The ATAC-seq peaks for each sample in each group were intersected with bedtools and retained only if all three samples had the same peak with a reciprocal minimal 50% overlap (3/3 peak). The resulting peak lists were used for all analyses in the project’s main figures. We also identified a list of variable peaks present in at least two of the three samples (2/3 peaks) that were used in supplementary analyses to support the main finding. Once we obtained the list of conserved peaks, the ATAC-seq reads mapping within the peaks’ intervals were counted with “bedtools coverage -count” generating the reads count matrix used for the plot in Figs. 2A and 3.

Peaks were considered “shared” between populations when they aligned at least 50% [using bedmap, from the bebops toolkit (*76*); using the option --fraction-either 0.5]. In the case that a larger peak in one population aligned with multiple smaller peaks in the other population, the smaller peaks were merged, and the reads were summed. Unique peaks were selected by subtracting every peak identified in one population from the list of 3/3 peaks of the other population (bedtools subtract -A). In this way, we identified peaks that were consistently present in the population of reference and completely absent in the other population. These peaks will be hereafter referred to as “unique ATAC-seq peaks.”

This strategy was chosen because ATAC-seq data are inherently noisy and sensitive to technical variability, sequencing depth differences, and sample-specific artifacts, and conservative consensus approaches are commonly used to minimize false positives in such contexts (*10*, *77*). While this approach reduces sensitivity, residual technical or batch effects are expected to manifest primarily as false negatives rather than false positives.

This replicate-based strategy was adopted to preserve replicate-level information at the peak-calling stage, which is essential for distinguishing regulatory elements that are consistently accessible across individuals from those that may be variable or polymorphic within populations. Because our biological questions focus on regulatory elements potentially under selection during population divergence, we prioritized the identification of reproducible and population-consistent accessible loci rather than maximizing peak number.

For the putative nonhomologous ATAC-seq peaks, the lineage-specific sequences obtained from the pangenome were intersected with the list of 3/3 peaks for each population. Subgroups of these ATAC-seq peaks were annotated to the closest gene using Homer’s “annotatePeaks.pl” (*78*). The closest gene to an ATAC-seq peak was used as a proxy for the potential target of the ATAC-seq peak. Genomic distances between ATAC-seq peaks and nearby genes were computed using pangenome coordinates. To assess the impact of reference expansion on peak-gene associations, closest-gene assignments were also recalculated using the original *H. e. demophoon* reference coordinates, and concordance between references was evaluated. In case of discordance, the closest gene from the original *H. e. demophoon* genome was used.

### Integration of ATAC-seq and *F_st_* patterns

To explore the potential relationships between *F*_*st*,_ selective sweep, and chromatin dynamics, we primarily used two approaches that aimed at identifying signals above background noise: the KS test (two-sample KS test) and a custom test based on the binomial test (Custom binomial test). All the analyses were performed in R using the *F_st_* calculated in 1-kb window size unless otherwise noted.

#### KS test

The KS test is a nonparametric statistical test used to understand whether two distributions come from the same underlying one. In this study, the first distribution was composed of the combined unique ATAC-seq peaks from the 3/3 list of the two populations under study; the second distribution was an average of 1000 random sampling of the same number of ranked points from the genome. The frequencies for the random sampling were modeled on the distribution of all the ATAC-seq peaks identified in the two populations. The test was run with the null hypothesis as “less” because a lower cumulative distribution function (CDF) represents higher values from the first distribution. Hence, this test verified that the unique ATAC-seq peak distribution had overall higher values of *F_st_* than the background in the populations studied. The test outputs *P* values as well as a *D* statistic, which can be interpreted as the maximum absolute difference between the CDF that we used as a proxy for the intensity of *F_st_* signal in unique ATAC-seq regions compared to the genome background for further analysis.

#### Custom binomial test

The Custom binomial test splits the genome into 10 *F_st_* intervals (increasing by 0.1) and then calculates an expected probability to find a unique ATAC-seq peak in each interval (using the average of 1000 random sampling). Next, a binomial test is performed for each interval using the empirical number of unique ATAC-seq peaks as the “number of successes,” the total number of ATAC-seq peaks in that *F_st_* interval as “number of trials,” and the average of random sampling as the “hypothesized probability of success.” The test outputs *P* values for each test (null hypothesis “greater”) that are then adjusted using the Holm-Bonferroni method correction, a more powerful and less conservative version of the classic Bonferroni correction. In summary, this approach measures whether there is a higher probability of finding a unique ATAC-seq peak in each *F_st_* interval than expected, highlighting a higher frequency of peaks in specific intervals compared to randomness. Last, overlap between population-differentiated ATAC-seq peaks showing elevated *F_st_* and the broad candidate *F_st_* regions identified using the genome-wide screening framework described above was assessed using bedtools intersect with a ≥1-bp overlap criterion.

#### Transcription factor binding site motif enrichment

Differential motif enrichment was performed on unique ATAC-seq peaks from the different adjacent pairs of populations using the XSTREME tool from the MEME suite (*79*). As a background model, we used the totality of ATAC-seq peaks identified for each population for their respective analyses; only motifs with an *E* value < 0.05 were considered significant.

### Evolution under progressively reduced gene-flow simulations

To support our empirical finding with a theoretical model, we conducted simulations of the evolutionary dynamics of two populations using SLiM 3 (*80*). The simulations shared the same structure and parameters, differing only in the migration rate after 2000 generations. We modeled two populations, each with two chromosomes. The first chromosome contained 2% of nucleotides under weak selection (*s* = 0.01), while the remaining sites were neutral (*s* = 0). The second chromosome was entirely neutral and served as a control, as neutral sites on the first chromosome could experience hitchhiking effects, whereas those on the second chromosome should not.

To improve computational efficiency, we scaled down the simulations by a factor of 100, following the guidelines in the SLiM manual (page 145). Specifically, we reduced both the population size and the number of generations by 100×, while increasing the mutation and recombination rates by the same factor. The effective population size used was 10,000, assuming the original was 1,000,000.

The mutation rate was scaled from 2.9 × 10^−9^ (*81*) to 2.9 × 10^−7^, and the recombination rate from 2 × 10^−8^ (*13*) to 2 × 10^−6^.

All simulations began with a migration rate of 0.5, which was then reduced after 2000 generations to one of the following: 0.1, 0.00001, 0.000000001, or 0. For each parameter set, we ran 100 replicate simulations.

### Phylogenetic comparisons

We analyzed the dynamics of nonhomologous ATAC-seq peaks outside of the *H. erato* species. This analysis was based on the HAL file generated by Cicconardi *et al.* (*57*), which was updated to include *H. e. hydara*, *H. e. favorinus*, *H. e. etylus*, *H. e. notabilis*, and *H. e. chestertonii*. A genomic region was considered present at a given tree tip if it contained at least 80% of the reference sequence.

### Putative nonhomologous ATAC-seq peaks coverage with resequencing

To assess the robustness of putative nonhomologous ATAC-seq peaks, we performed an additional coverage-based analysis using whole-genome resequencing data. For each resequencing sample, read coverage was quantified within each putative nonhomologous ATAC-seq peak and within its local genomic background using *mosdepth* (*82*). Background regions were defined as the ±1-kb flanking intervals surrounding each peak, excluding the peak itself. Mean read depth was calculated separately for peak and background regions for each sample.

Coverage values were then aggregated across samples and populations into a unified table reporting mean depth per peak, per sample, and per region (peak or background), which served as input for downstream classification analyses in R. For each peak and sample, mean coverage within the peak was compared to local background coverage. Samples with insufficient background coverage (<10 reads) were classified as uninformative. Among informative samples, peaks with coverage ≤10% of background were classified as absent, while all others were classified as present.

At the population level, a peak was considered absent if at least 80% of informative samples from that population were classified as absent; otherwise, it was considered present. Population-level calls were then aggregated across populations to classify each peak as shared (present in all populations), variable (present in multiple but not all populations), exclusive (present in only one population), or uninformative. This framework explicitly distinguishes true biological absence from low sequencing coverage and avoids misclassifying missing signal as biological absence, resulting in a conservative classification of chromatin accessibility across populations.

### Genome architecture variability analysis

We studied the synteny among sequences present in all six populations and identified possible transpositions. The positions of the transpositions between conserved regions in different genomes were calculated with a custom script from the XMFA file of the original pangenome. A transposition was considered real only if (i) it happened between chromosomes or moved at least 100 kb from the reference; (ii) the sequence moved was longer than 5 kb; and (iii) we identified at least 20 reads from the genome sequencing mapping across the beginning or the end of the transposition (that mapped partially on the transposed sequence and part of the surrounding region), indicating that the transposition was not due to a technical misassembly of the scaffold. For this last step, we used minimap2 (*83*) to map the sequences and SAMtools to map from a mapping quality of at least 10.

### TE identification

To identify TEs, we used RepeatMasker (*84*) on the consensus pangenome, using the TE library generated by Ruggieri *et al.* (*25*) with default parameters.

### Animal experimentation

Butterfly collection, rearing, and experimental procedures were conducted in accordance with permits and regulations from the relevant authorities. The necessary butterfly collection permits were obtained from the governments of each country, and importation permits were obtained from the Ministry of the Environment of Panama, according to Panamanian Government and Smithsonian Tropical Research Institute (STRI) regulations. Rearing and experimentation complied with local government regulations on containment and handling. Samples collected in Peru were obtained under permits 0289-2014-MINAGRI-DGFFS/DGEFFS, 020-014/GRSM/PEHCBM/DMA/ACR-CE, and 040-2015/GRSM/PEHCBM/DMA/ACR-CE, granted to N. Rosser, and samples from Panama were collected under permits SEX/A-3-12, SE/A-7-13, and SE/AP-14-18.
