# Supplementary material for: Multilocus basis of incipient reproductive isolation in hybridizing populations is revealed by pangenomic and epigenetic divergence
Source: Sci Adv. 2026 Aug 21;12(34):eadz6665. doi: 10.1126/sciadv.adz6665 (PMC13496208; doi:10.1126/sciadv.adz6665)
Supplement: Supplementary file 1 [file sciadv.adz6665_sm.pdf]

Science Advances
AAAS

Supplementary Materials for

Multilocus basis of incipient reproductive isolation in hybridizing populations is revealed by pangenomic and epigenetic divergence

Angelo A. Ruggieri *et al.*

Corresponding author: Angelo A. Ruggieri, angelo.ruggieri@upr.edu; Riccardo Papa, riccardo.papa@upr.edu

Sci. Adv. **12**, eadz6665 (2026)
DOI: 10.1126/sciadv.adz6665

This PDF file includes:

Figs. S1 to S14
Tables S1 to S7

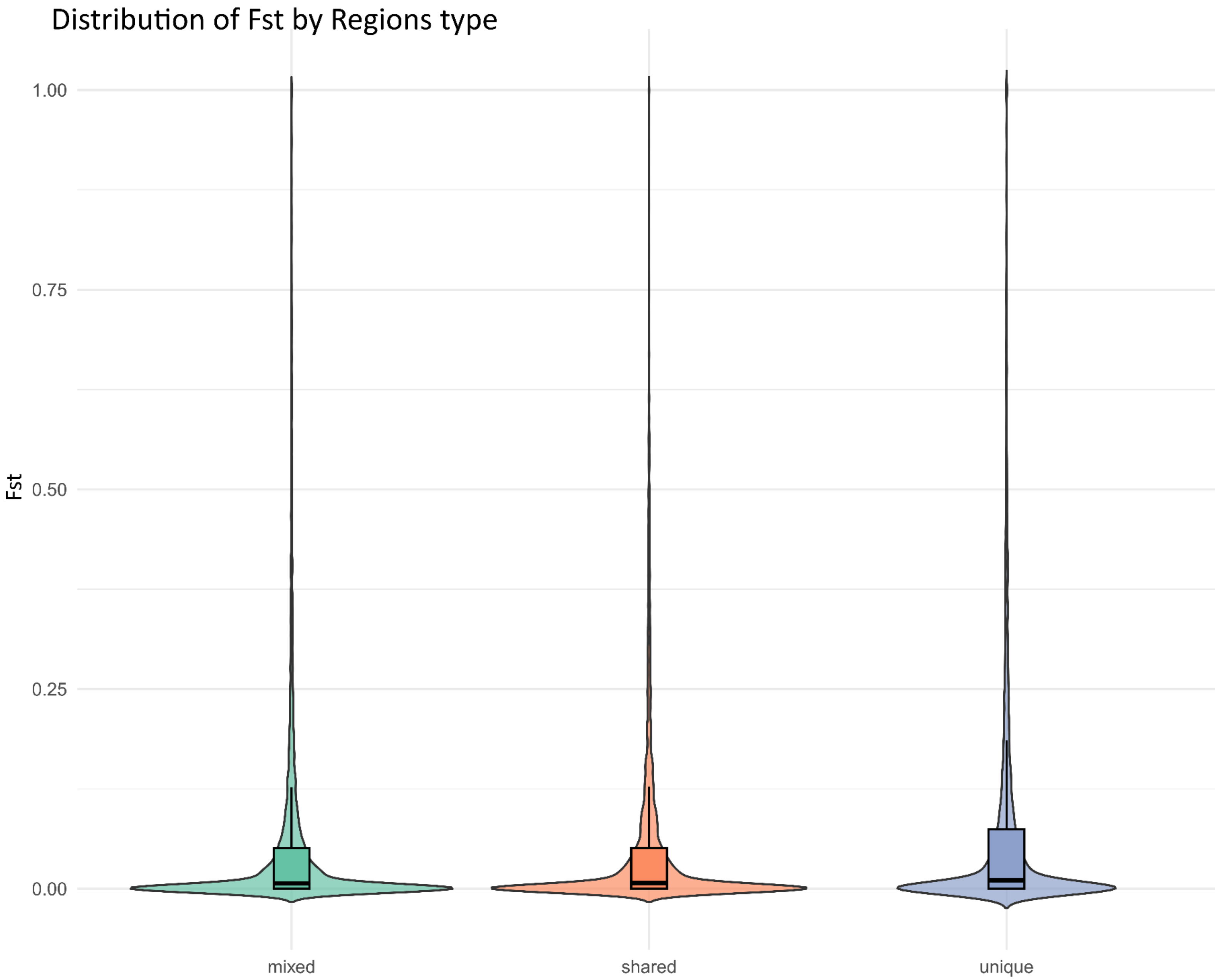

Figure S1. F_{st} in pangenome sequence categories. Violin plot showing genome-wide F_{st} distributions across sequence categories: unique (regions exclusive to a single population in the pangenome), shared (regions present in all six populations), and mixed (regions present in more than one but fewer than six populations)..

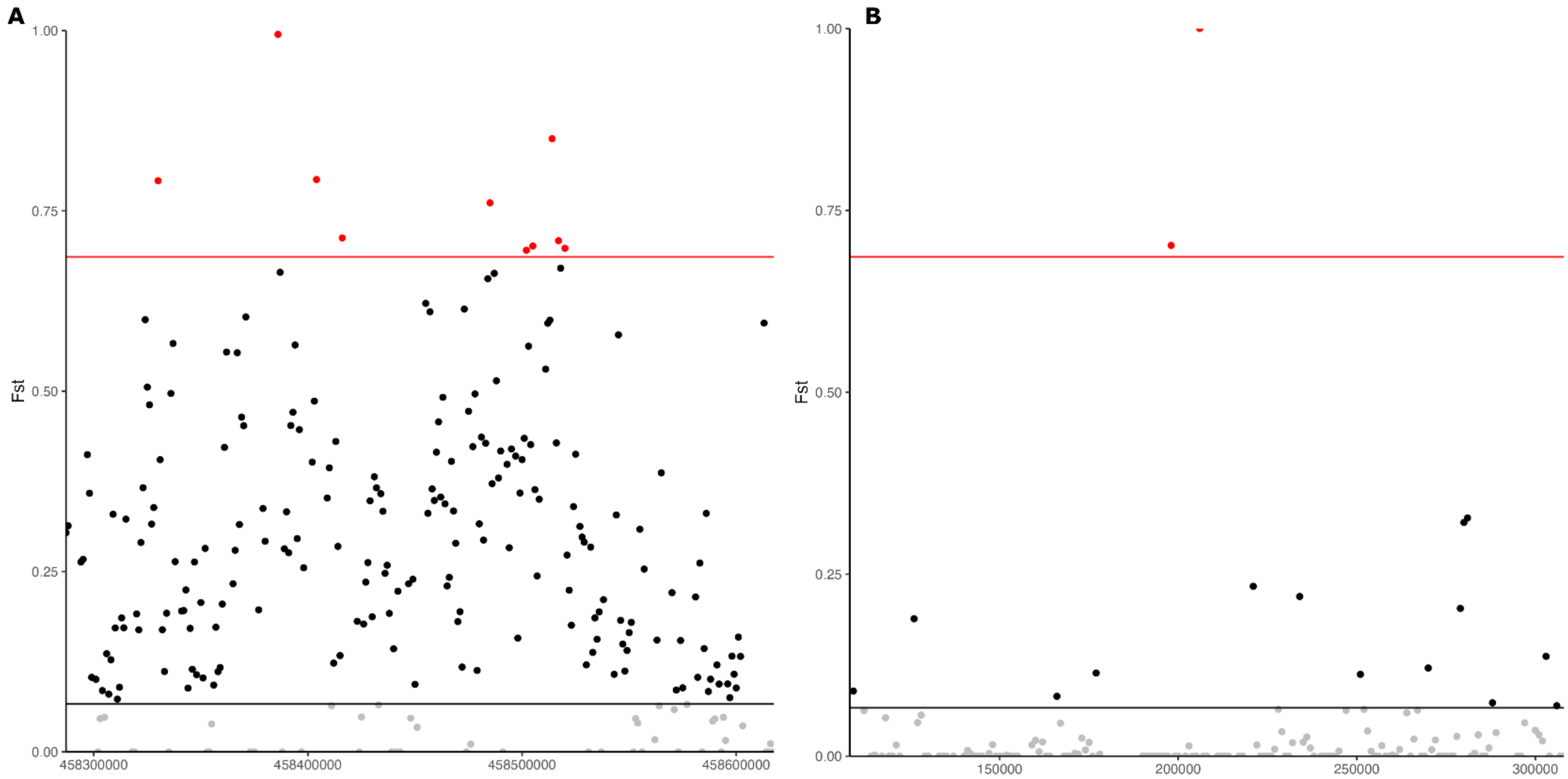

Figure S2:Visual representation of F_{st} results obtained using the new filtering strategy. (A) Example of a true signal identified by the updated system. Several points exceed the 99th percentile threshold (red line), accompanied by a high number of values above the genome-wide mean (black line). (B) Example of a spurious ("fake") signal. Although a few points exceed the 99th percentile threshold, the region lacks a sufficient number of values above the mean.

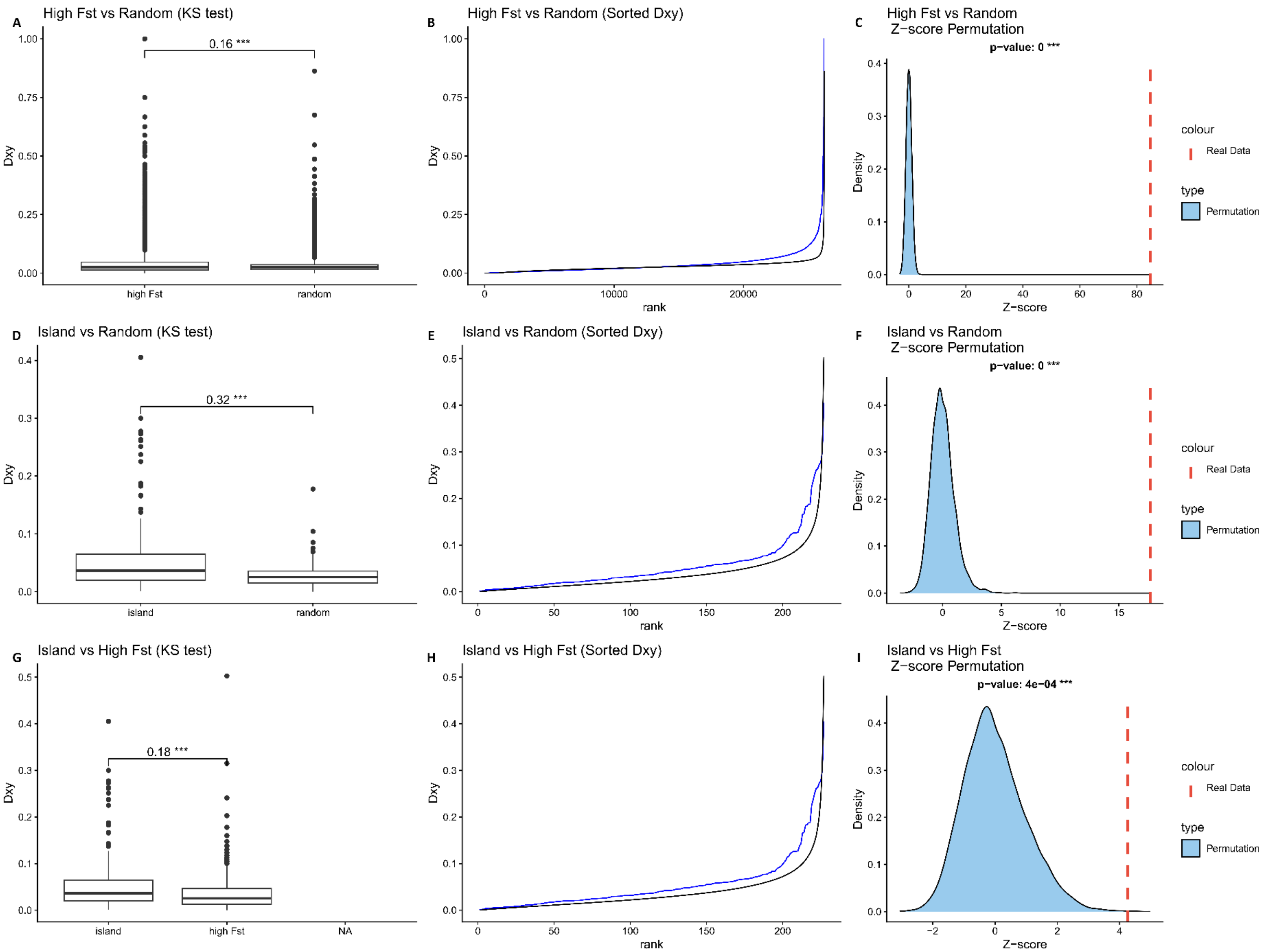

Figure S3: Distribution of D_{xy} in high-F_{st} regions (A) Boxplot showing D_{xy} values in high-F_{st} regions identified from the full 1 kb window F_{st} scan, compared to an equal number of randomly sampled low-F_{st} windows. (B) Distribution of ranked D_{xy} values for the same datasets. (C) Results of permutation tests comparing the two distributions. (D–F) Same analyses as in (A–C), but restricted to high-F_{st} regions identified as significant in the 1 kb window analysis. (G–I) Same analyses as in (A–C), comparing significant high-F_{st} regions to an equal number of high-F_{st} windows drawn from the full 1 kb dataset. Background distributions were generated using 1,000 random samplings for each comparison, and permutation tests were performed with 10,000 iterations.

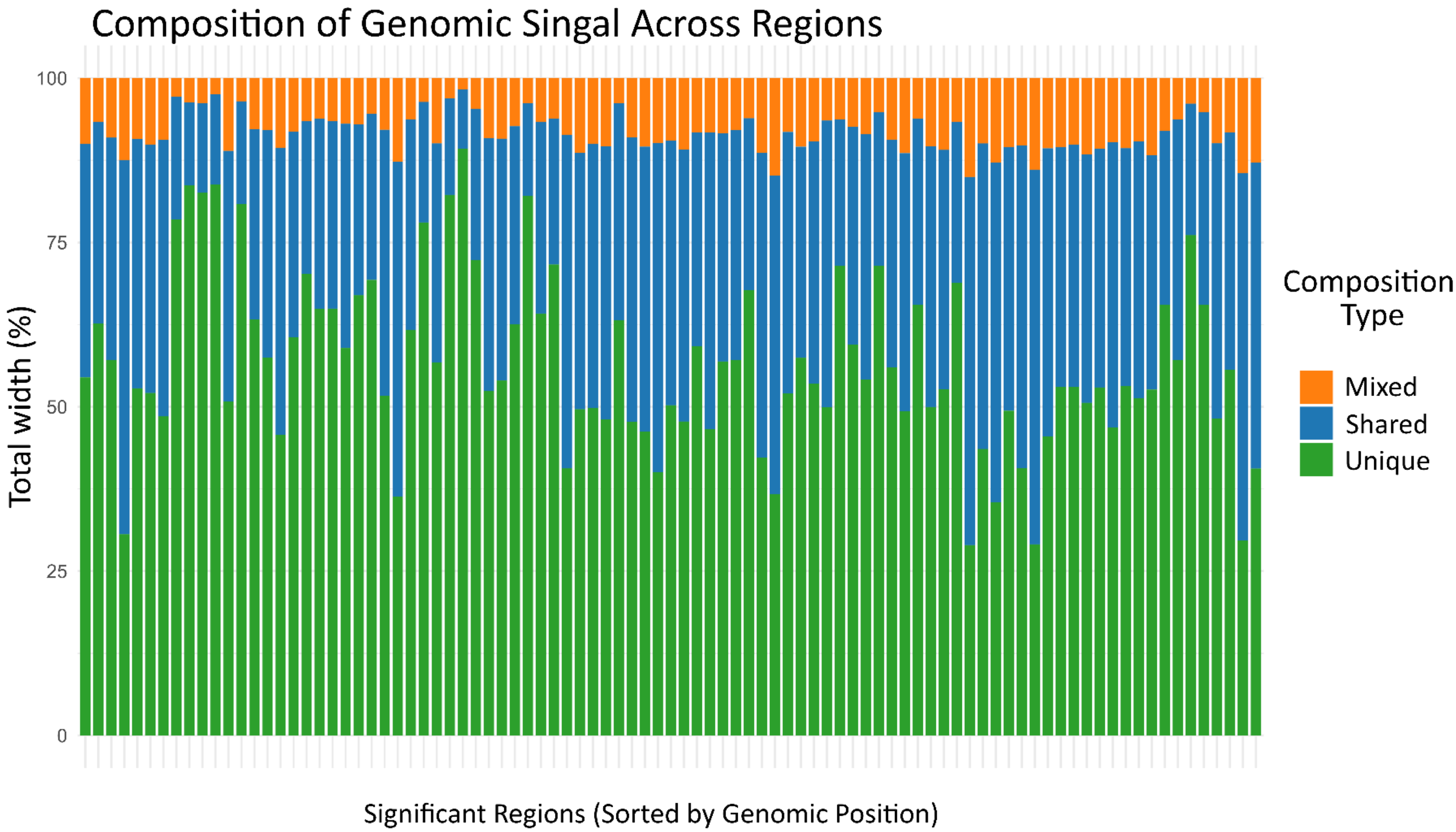

Figure S4. Composition of genomic signal across high-F_{st} regions. Stacked bar plot showing the relative contribution of sequence categories across the 91 high-F_{st} regions identified in the H. demophoon–H. hydara comparison. Each bar represents a region (ordered by genomic position), with proportions of unique (present in a single population), shared (present in all populations), and mixed (present in more than one but not all populations) sequences

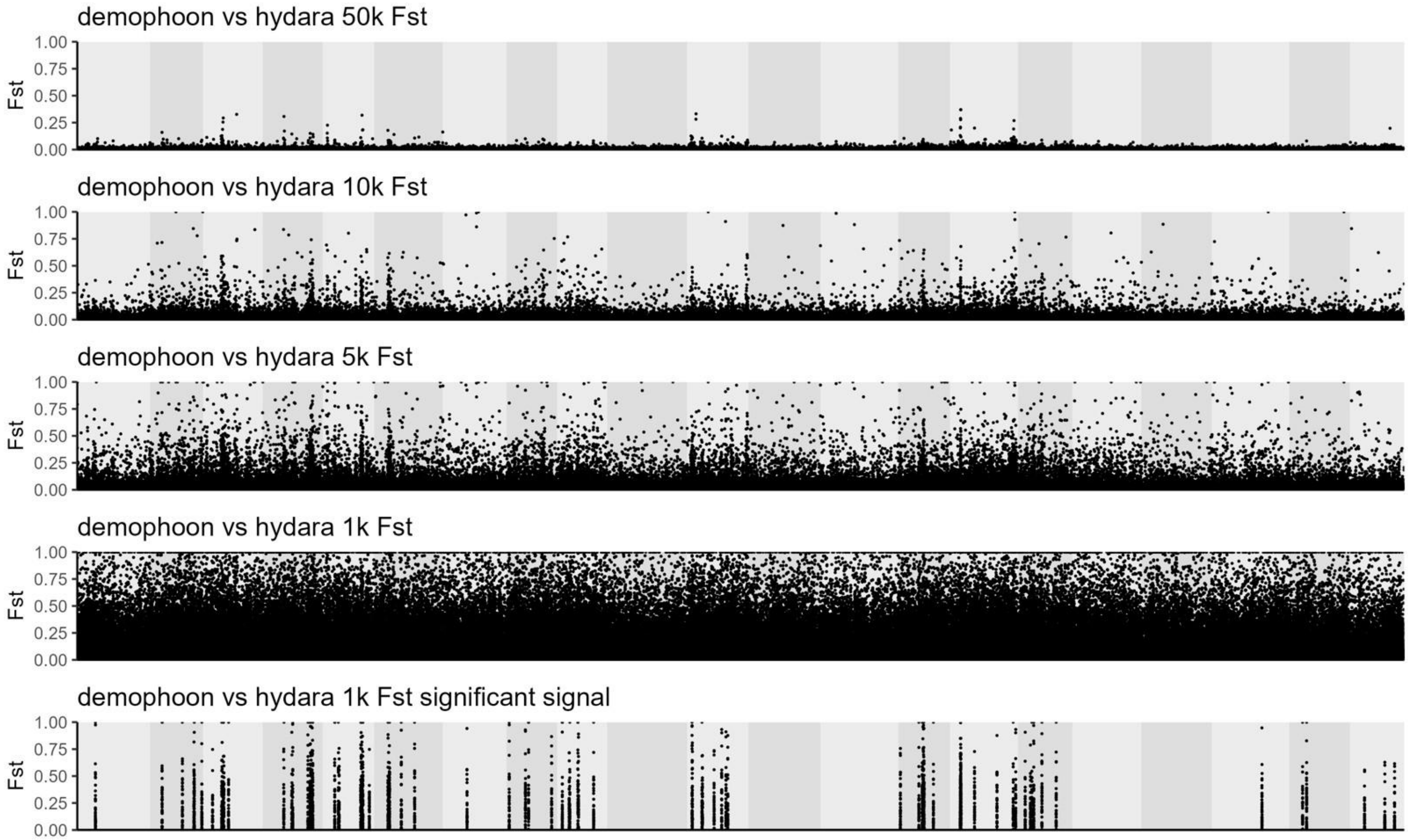

Figure S5: Genome-wide view of F_{st} calculated in different non-overlapping window sizes. The first panel shows genome-wide F_{st} values calculated in 50k windows, the second panel in 10k windows, the third panel in 5k windows, and the fourth panel in 1k windows. The final panel highlights only the significant regions identified in the 1k-window F_{st} analysis.

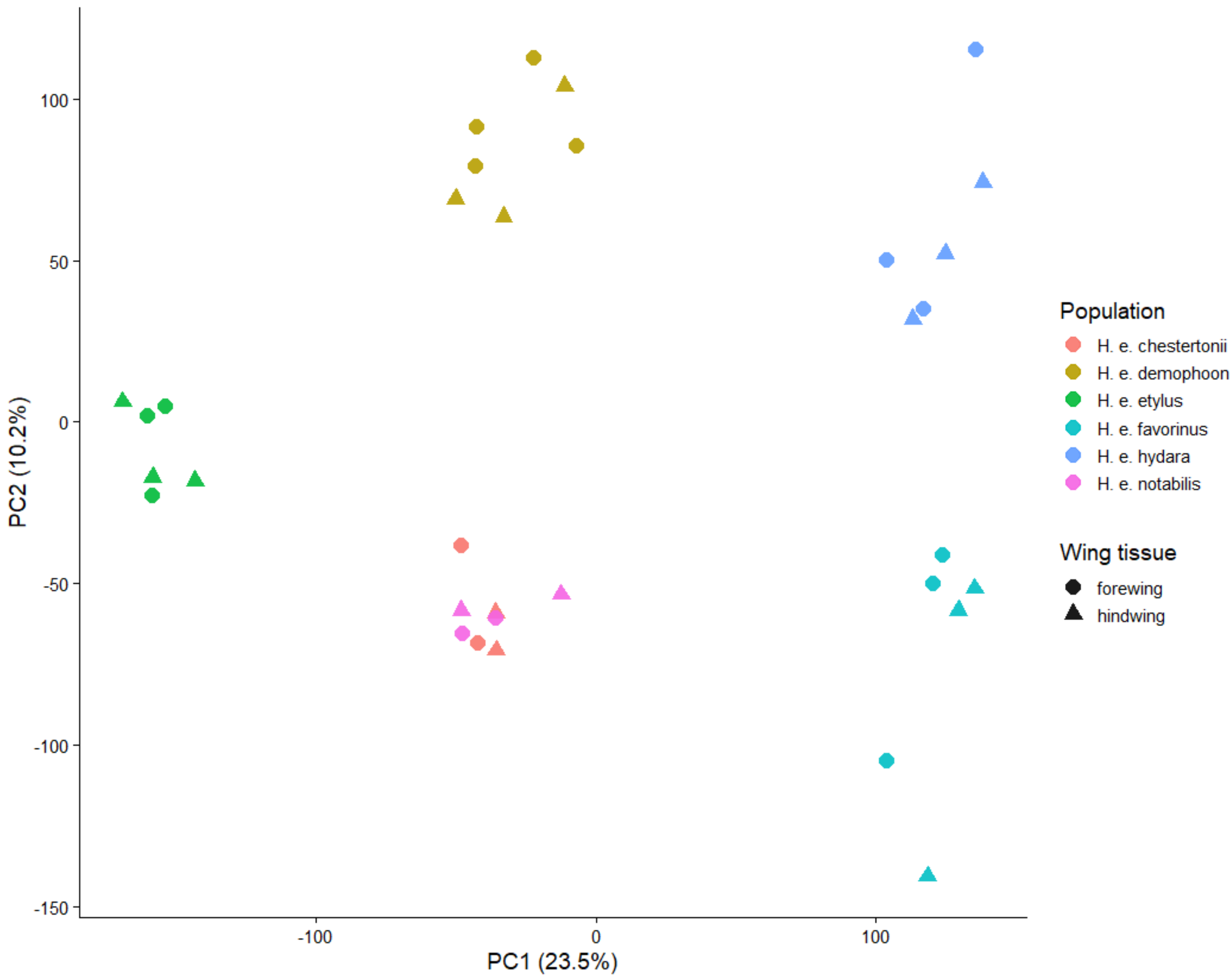

Figure S6: PCA of ATAC-seq data. Principal component analysis based on the full ATAC-seq dataset for the six populations analyzed.

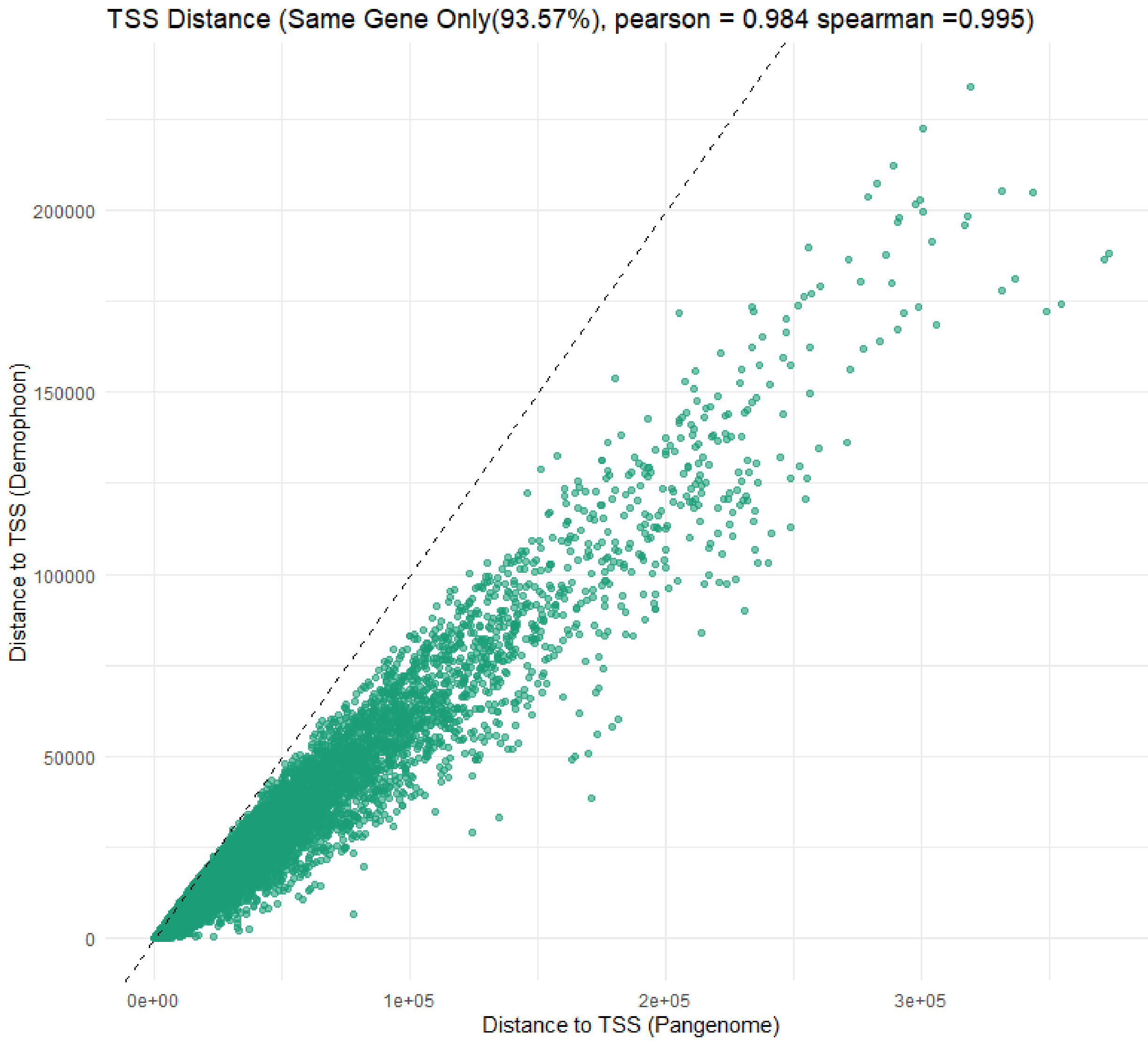

Figure S7:Comparison of ATAC-seq peak distances to the nearest transcription start site (TSS) measured on the *H. demophoon* reference genome and the pan-genome. Each point represents an ATAC-seq peak, with the distance to the closest annotated gene TSS calculated on the pan-genome (x-axis) and on the *H. demophoon* genome (y-axis). Only peaks assigned to the same gene in both annotations are shown (93.57%). The dashed line indicates the 1:1 relationship. Pearson and Spearman correlation coefficients are reported.

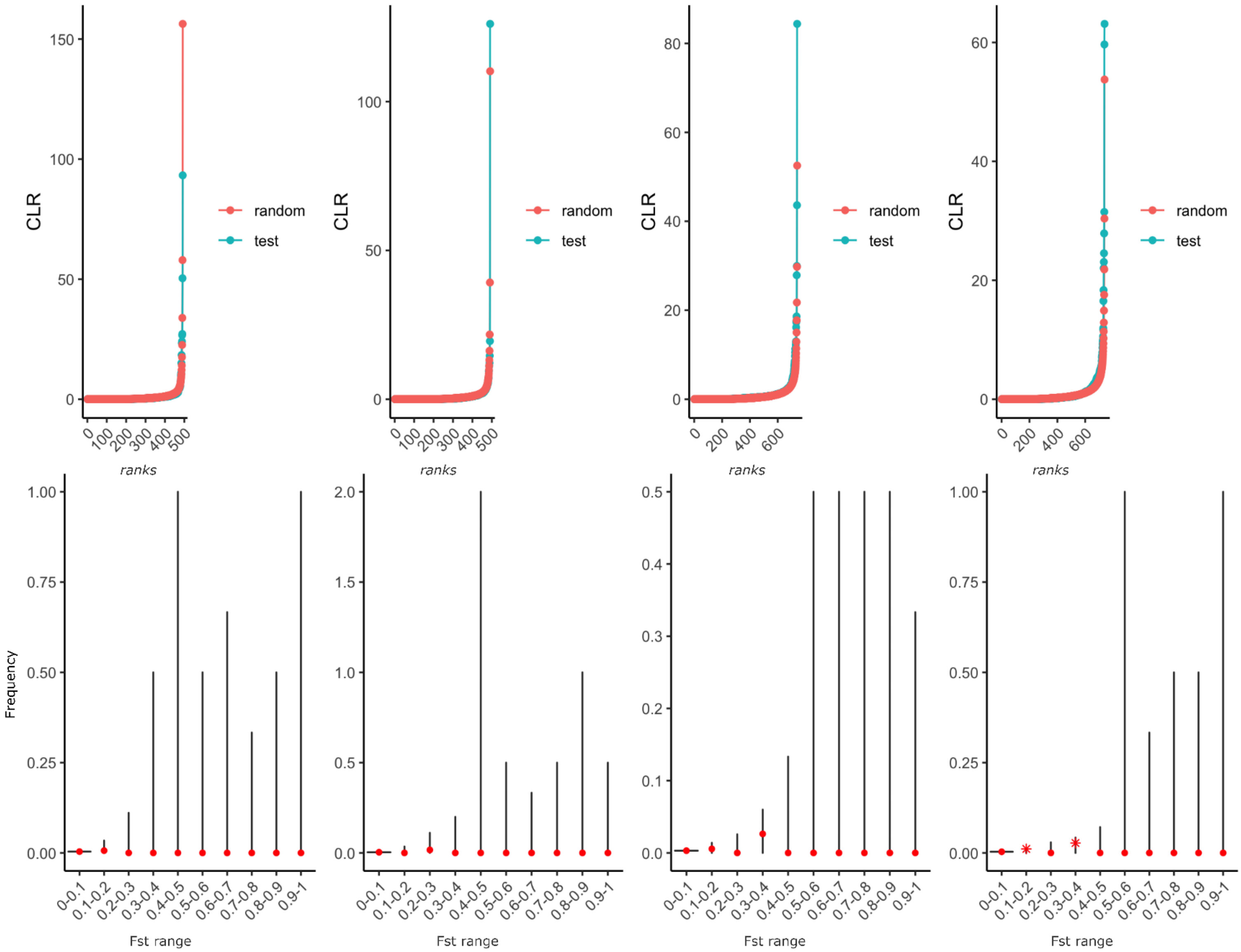

Figure S8: Statistical tests for selective sweep analysis. The first set of tests includes the Kolmogorov-Smirnov (KS) test performed on CLR values for different geographical morphs: (A) *H. e. demophoon*, (B) *H. e. hydara*, (C) *H. e. notabilis*, and (D) *H. e. etylus*. The second set of tests involves a binomial test applied to the same geographical morphs, denoted as (E), (F), (G), and (H), respectively.

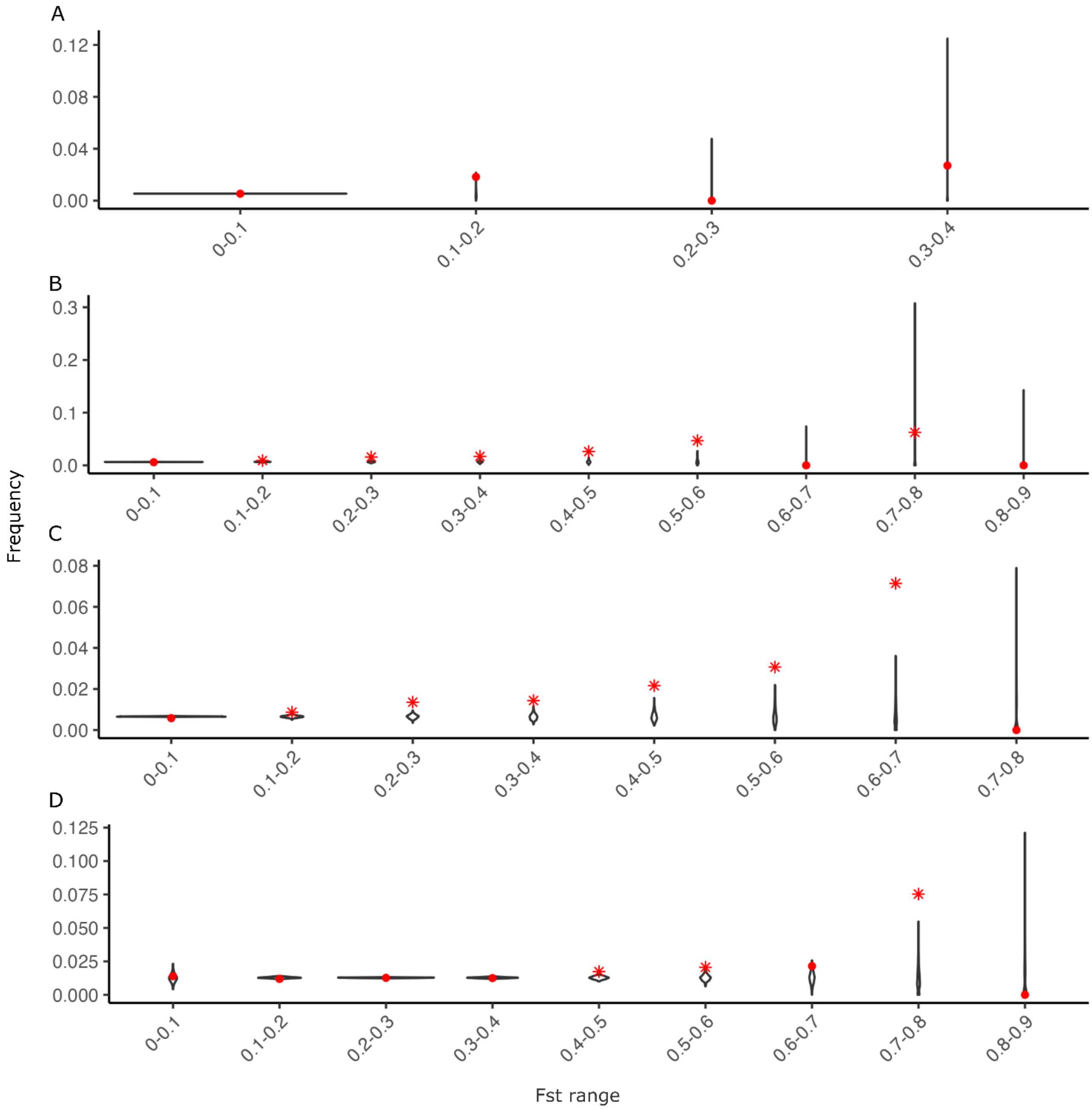

Figure S9: Special application of the binomial test conducted on 50k-window F_{st} values for *H. e. demophoon* compared to four populations. (A) Comparison with *H. e. hydara*; (B) comparison with *H. e. favorinus*; (C) comparison with *H. e. etylus*; (D) comparison with *H. e. chestertonii*.

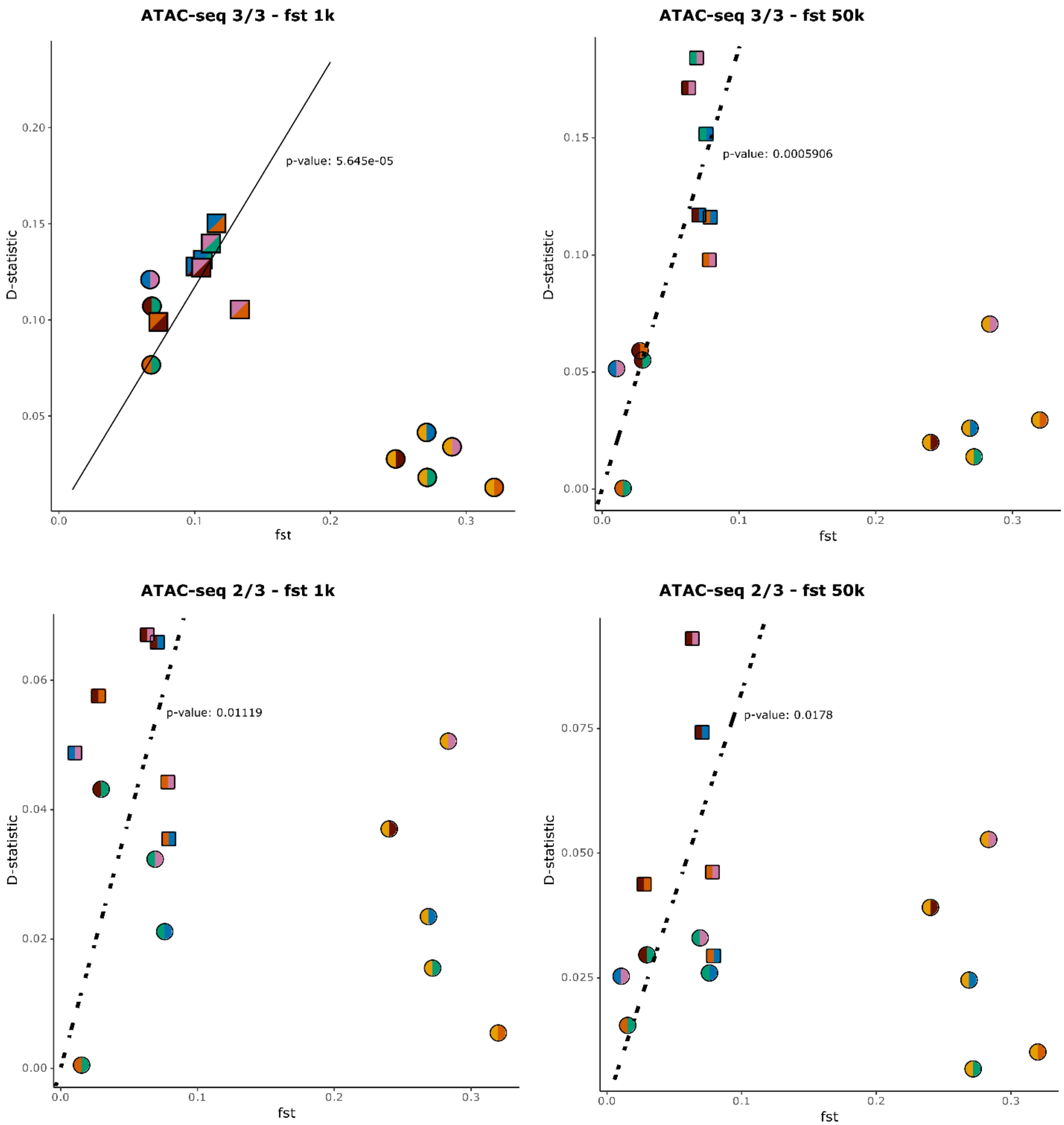

Figure S10: Summary of 15 pairwise Kolmogorov-Smirnov (KS) tests performed on different datasets. The top-left panel shows the comparison between ATAC-seq peaks identified in all 3 out of 3 samples and F_{st} values measured in 1k windows. The top-right panel displays the comparison between ATACseq peaks identified in all 3 out of 3 samples and F_{st} values measured in 50k windows. The bottom-left panel presents comparisons involving ATAC-seq peaks identified in at least 2 out of 3 samples with F_{st} measured in 1k windows, while the bottom-right panel shows comparisons with F_{st} measured in 50k windows. For each plot, a linear regression performed on significant comparisons, along with its p-value, is reported.

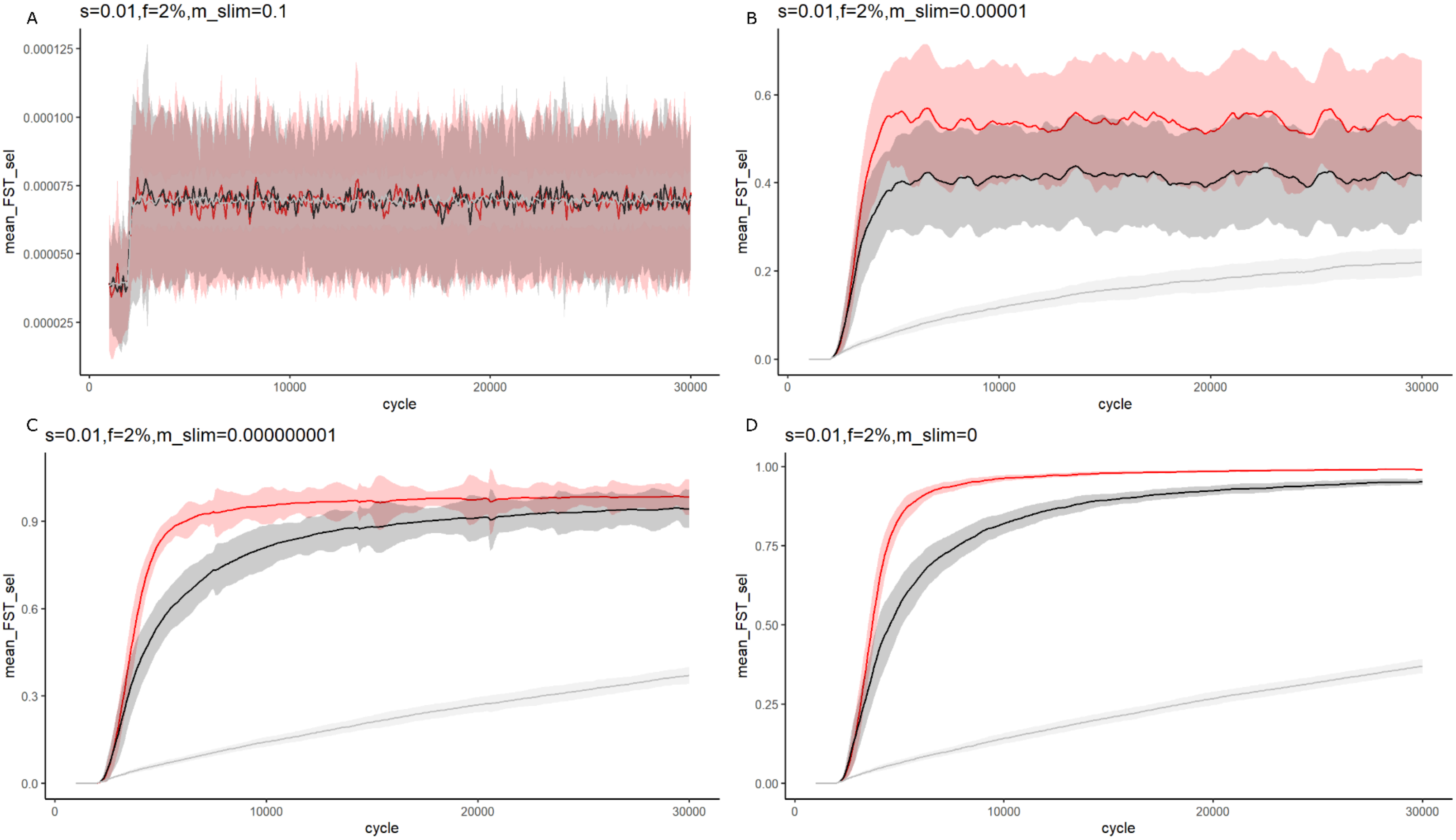

Figure S11: Evolution simulations with different levels of gene flow. A total of 100 simulations were performed for each panel. The red line represents F_{st} for the region under weak selection, the black line represents F_{st} for a neutral region on the same chromosome (subject to potential hitchhiking), and the gray line represents F_{st} for a completely neutral chromosome. The shaded area indicates the variance across simulations. All simulations used identical parameters, including generations and population sizes, with only the gene flow rate changing after 2,000 generations. (A) Migration rate = 0.1; (B) migration rate = 0.00001; (C) migration rate = 0.000000001; (D) migration rate = 0.

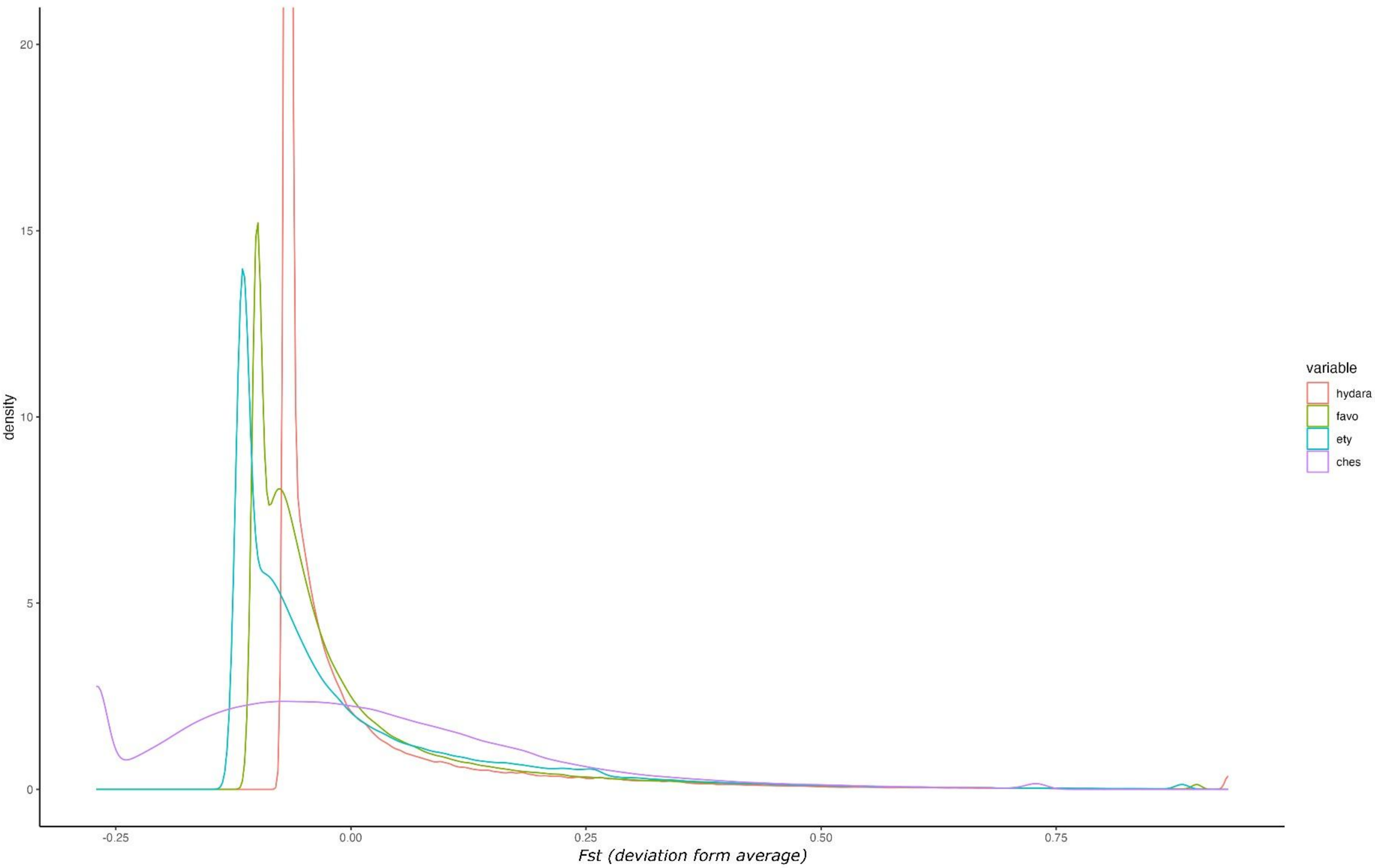

Figure S12**:** *H. e. etylus*, and *H. e. notabilis*. The x-axis represents the deviation from the mean F_{st} value for each data point within the respective populations

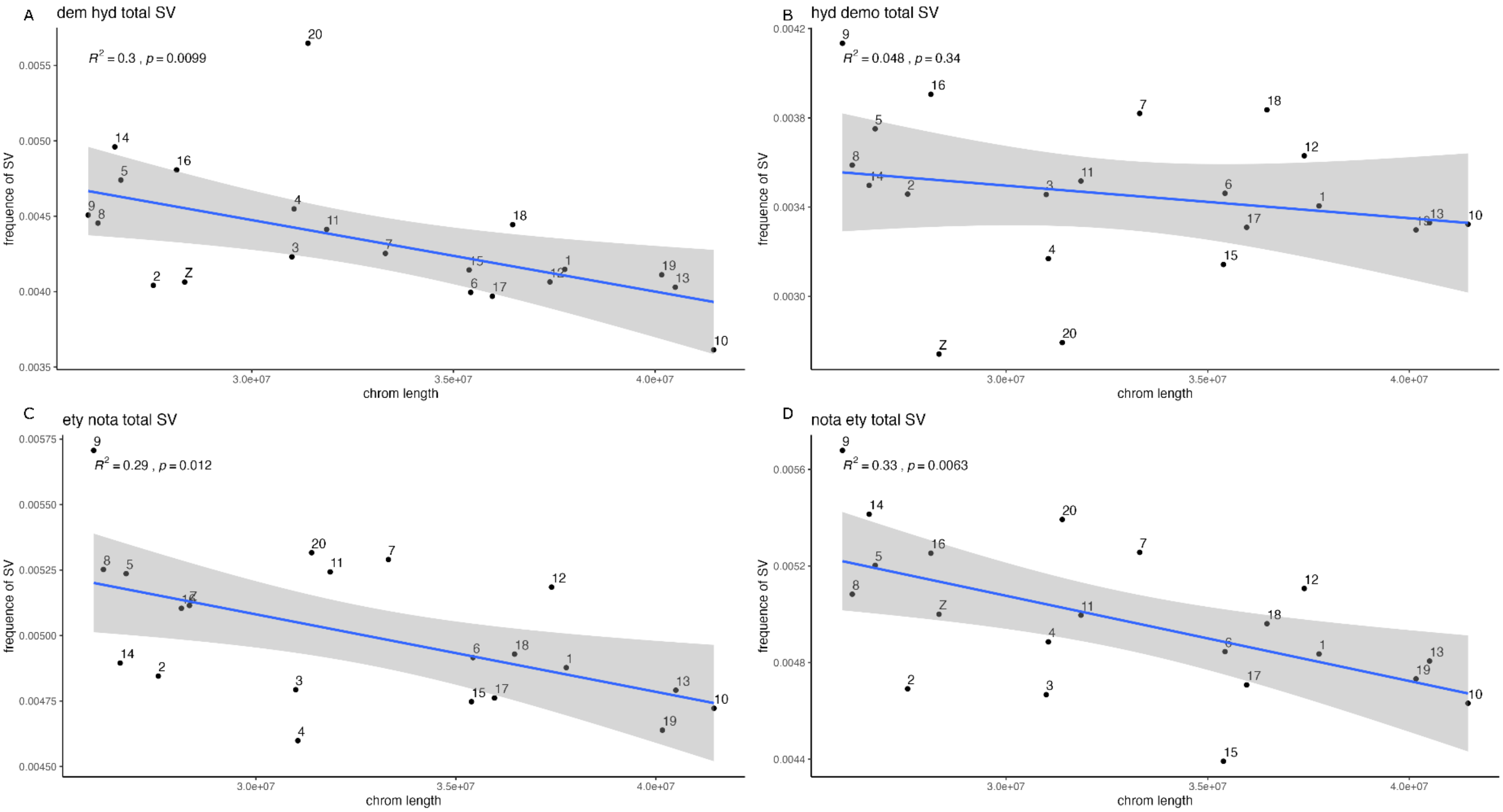

Figure S13: Correlation between the frequency of structural variants (SV) and chromosome length. Each panel includes the reported R-squared values and the significance of linear regression analyses. A) comparison between *H. e. demophoon* and *H. e. hydara.* B) reverse comparison (*H. e. hydara* vs *H. e. demophoon*. C) comparison between *H. e. etylus* and *H. e. notabilis.* D) reverse comparison (*H. e. notabilis* vs *H. e. etylus*).

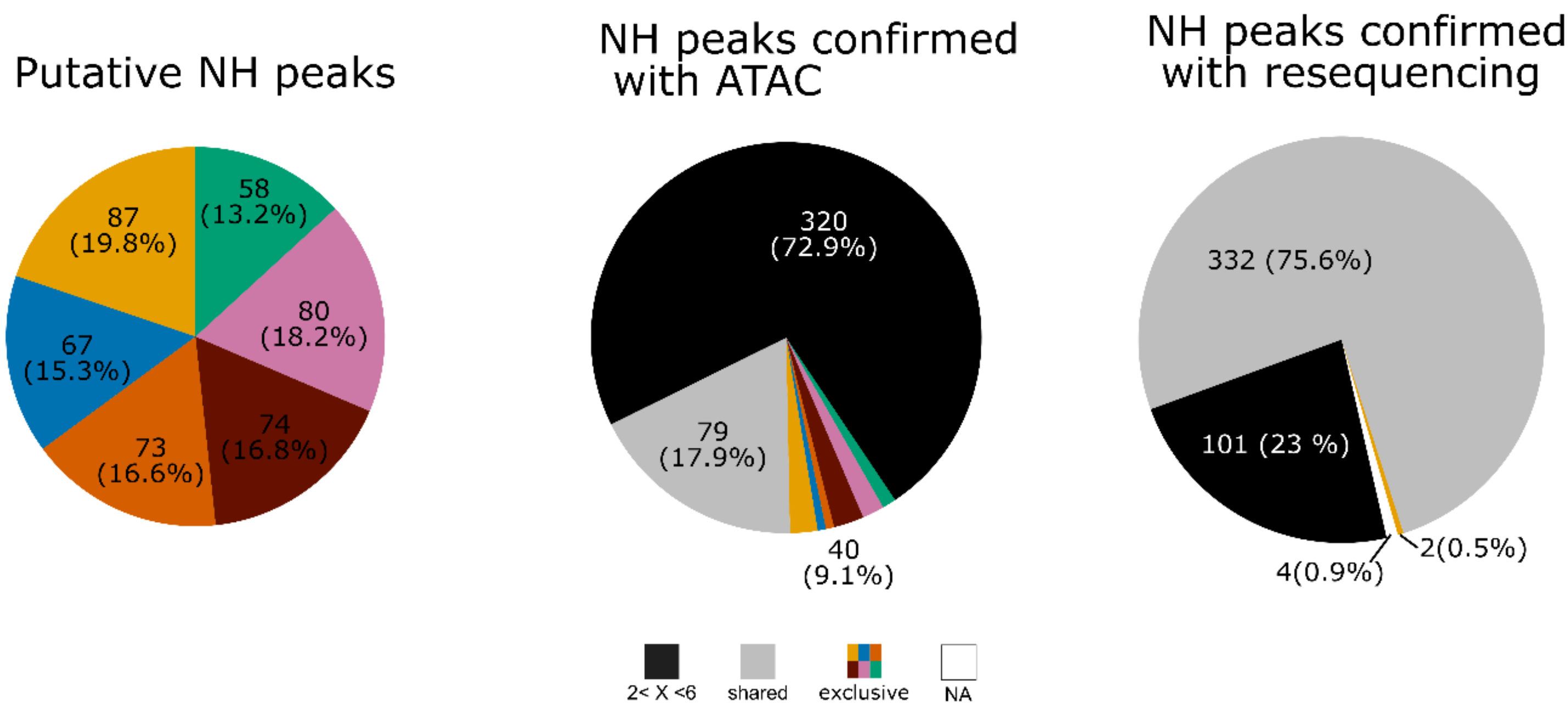

Figure S14: Characterization and validation of putative non-homologous (NH) ATAC-seq peaks. Left: Distribution of putative NH peaks identified prior to data integration. Middle: Subset of NH peaks after integration with ATAC-seq data, showing peaks confirmed across multiple samples, shared between datasets, or exclusive to individual datasets. Right: Subset of NH peaks after integration with whole-genome resequencing data, indicating peaks supported by sequence evidence. Colors denote the different confirmation categories (2 < x < 6, shared, exclusive, NA); NA indicates regions with insufficient read coverage to be informative. Numbers represent counts with corresponding percentages.

pop	genome size	bp NH	NH perc %	npeaks	unique	unique % pop	confirmed NH peak
demophoon	377207547	39762511	11	16455	67	0.41	3
hydara	356541278	51685917	14	16242	80	0.49	8
favorinus	363693562	39369963	11	15280	74	0.48	11
etylus	336746453	32075924	10	18811	73	0.39	3
notabilis	338400759	43070876	13	13165	58	0.44	5
chestertonii	326196349	50560864	16	22804	87	0.38	10

total	shared	shared %	unique	unique %
29633	8799	29.69	439	1.48

Table S1: Summary of genomic and ATAC-seq statistics. The upper section reports population-specific statistics, and the lower section summarizes ATAC-seq peaks across all populations.

Population	Sample	Mapped (pangenome)	Mapped (single reference)	Error (pangenome)	Error (single reference)
demophoon	Pet_ED3	99.82	97.563	0.0367	0.03695
demophoon	Pet_ED4	99.289	96.241	0.0363	0.03655
demophoon	Pet_ED5	99.749	97.217	0.03591	0.03626
demophoon	Pet_ED6	99.899	97.612	0.03627	0.03652
demophoon	STRIWOM5353	98.547	94.795	0.03942	0.03966
demophoon	STRIWOM5362	98.625	96.329	0.03893	0.03766
demophoon	STRI_WOM_0033	99.595	96.329	0.03708	0.03766
favorinus	NCS_0473	98.449	94.231	0.037531	0.043314
etylus	BC_3280	98.421	93.434	0.037495	0.044818
hydara	STRI_WOM_0039	99.644	96.401	0.036983	0.037448
chestertonii	STRI_WOM_0057	98.283	94.037	0.037618	0.040774
notabilis	notabilis_02	97.715	92.566	0.039444	0.045772

Table S2: summary of the differnece in mapping reference between the single refrence ,H. e. demophoon versus the pangenome.

Total (pangenome)	Total (single reference)	Missing (pangenome)	Missing (single reference)	Callable (pangenome)	Callable (single reference)	Variant (pangenome)
635548999	298650628	361357803	84183608	274191196	214467020	30786816
635548999	298650628	365009672	86790568	270539327	211860060	30527965
635548999	298650628	379988801	97673371	255560198	200977257	29267664
635548999	298650628	367906097	85603607	267642902	213047021	30294699
635548999	298650628	351474519	78734906	284074480	219915722	31266078
635548999	298650628	356585749	82197002	278963250	216453626	30974521
635548999	298650628	378756954	97174977	256792045	201475651	29338054
635548999	298650628	397203586	109935869	238345413	188714759	28238916
635548999	298650628	391392032	112746514	244156967	185904114	27818572
635548999	298650628	375863201	94527101	259685798	204123527	29585825
635548999	298650628	398493100	105736679	237055899	192913949	27452595
635548999	298650628	383053843	96444425	252495156	202206203	29218070

Variant (single reference)	Invariant (pangenome)	Invariant (single reference)	CallRate (pangenome)	CallRate (single reference)	VarDensity (pangenome)	VarDensity (single reference)
30198335	243404380	184268685	0.4314	0.7181	0.1123	0.1408
29857140	240011362	182002920	0.4257	0.7094	0.1128	0.1409
28408334	226292534	172568923	0.4021	0.673	0.1145	0.1414
29992623	237348203	183054398	0.4211	0.7134	0.1132	0.1408
30830441	252808402	189085281	0.447	0.7364	0.1101	0.1402
30547917	247988729	185905709	0.4389	0.7248	0.111	0.1411
28495309	227453991	172980342	0.404	0.6746	0.1142	0.1414
27165109	210106497	161549650	0.375	0.6319	0.1185	0.1439
26420520	216338395	159483594	0.3842	0.6225	0.1139	0.1421
28851738	230099973	175271789	0.4086	0.6835	0.1139	0.1413
27509068	209603304	165404881	0.373	0.646	0.1158	0.1426
28930870	223277086	173275333	0.3973	0.6771	0.1157	0.1431

depth (pangenome)	depth (single reference)
23.7302	32.9727
22.8841	32.4826
18.0653	27.6761
21.4406	32.8408
19.1556	28.9119
18.562	28.1054
14.7709	22.5989
14.1969	21.2925
12.5144	18.5716
15.6594	23.9419
16.0864	24.0999
16.3667	24.3341

demophoon vs hydara genes in Fst signals

evm.TU.Herato0206.31	lysosomal acid glucosylceramidase-like
evm.TU.Herato0206.32	farnesol dehydrogenase-like
evm.TU.Herato0211.98	uncharacterized protein LOC112048264
evm.TU.Herato0211.99	RNA-directed DNA polymerase from mobile element jockey-like isoform X1
evm.TU.Herato0215.128_evm.TU.Herato0215.129	phenoloxidase subunit 1
evm.TU.Herato0215.130	phenoloxidase subunit 1
evm.TU.Herato0215.43	uncharacterized protein LOC117985158
evm.TU.Herato0215.44	fatty acid synthase-like
evm.TU.Herato0215.45	uncharacterized protein LOC117985158
evm.TU.Herato0301.47	rootletin
evm.TU.Herato0309.1	Nucleic-acid-binding protein from transposon X-element
evm.TU.Herato0412.1	uncharacterized protein LOC113402078 isoform X2
evm.TU.Herato0412.2	---NA---
evm.TU.Herato0413.1	Retrovirus-related Pol polyprotein from transposon opus-like Protein
evm.TU.Herato0413.2	RNA-directed DNA polymerase from mobile element jockey
evm.TU.Herato0503.111	carboxylesterase 1C
evm.TU.Herato0503.73	beta-1,4-N-acetylgalactosaminyltransferase bre-4-like
evm.TU.Herato0507.1	neuropeptide CCHamide-1 receptor
evm.TU.Herato0508.67	uncharacterized protein LOC113394814 isoform X1
evm.TU.Herato0601.102	protein unc-13 homolog C-like
evm.TU.Herato0601.103	RNA-directed DNA polymerase from mobile element jockey-like
evm.TU.Herato0601.104	protein timeless homolog
evm.TU.Herato0601.105	protein timeless homolog isoform X1
evm.TU.Herato0701.327	ecdysteroid 22-kinase
evm.TU.Herato0701.328	piggyBac transposable element-derived protein 4-like
evm.TU.Herato0801.165	Down syndrome cell adhesion molecule-like protein Dscam2
evm.TU.Herato0801.17	nephrin-like
evm.TU.Herato0821.65	polyribonucleotide nucleotidyltransferase 1, mitochondrial
evm.TU.Herato0821.66	3'-5' ssDNA/RNA exonuclease TatD
evm.TU.Herato0901.158	proteasome assembly chaperone 2
evm.TU.Herato0901.159	peroxisomal biogenesis factor 3
evm.TU.Herato0901.303	unnamed protein product
evm.TU.Herato0901.57	tachykinins-like
evm.TU.Herato1106.1	TRAS3 protein
evm.TU.Herato1106.2	craniofacial development protein 2-like
evm.TU.Herato1108.401	probable serine/threonine-protein kinase DDB_G0286465
evm.TU.Herato1108.68	probable uridine nucleosidase 2
evm.TU.Herato1108.71	gustatory receptor for sugar taste 64f-like
evm.TU.Herato1408.178,evm.TU.Herato1408.179	sex peptide receptor-like isoform X2
evm.TU.Herato1411.103	dynein heavy chain 10, axonemal
evm.TU.Herato1505.164,evm.TU.Herato1505.165,evm.TU.Herato1505.86	PARN
evm.TU.Herato1505.85	Cortex
evm.TU.Herato1505.87	dynein assembly factor 5, axonemal
evm.TU.Herato1512.5	seminal fluid protein HACP057

evm.TU.Herato1601.77,evm.TU.Herato1601.78	RNA-directed DNA polymerase from mobile element jockey
evm.TU.Herato1603.2	zinc finger DNA binding protein
evm.TU.Herato1908.17	uncharacterized protein LOC113400081
evm.TU.Herato2001.136	L-ascorbate oxidase-like
evm.TU.Herato2001.137,evm.TU.Herato2001.138	adenosine receptor A2b
evm.TU.Herato2101.158	multidrug resistance protein homolog 49-like
evm.TU.Herato2101.159	dendritic arbor reduction protein 1-like isoform X1
evm.TU.Herato2101.388,evm.TU.Herato2101.389,evm.TU.Herato2101.390	major facilitator superfamily domain-containing protein 6

notabilis vs etylus genes in Fst signal

evm.TU.Herato0101.589	chromodomain-helicase-DNA-binding protein 7 isoform X1
evm.TU.Herato0101.590	probable NADH dehydrogenase [ubiquinone] iron-sulfur protein 6, mitochondrial
evm.TU.Herato0101.591	UPF0184 protein C9orf16 homolog
evm.TU.Herato0101.592	ras-related protein Rab-14
evm.TU.Herato0101.593	carnitine O-acetyltransferase-like isoform X1
evm.TU.Herato0101.594	kinesin-associated protein 3
evm.TU.Herato0206.21	fatty acid synthase-like
evm.TU.Herato0209.38_evm.TU.Herato0209.39	phenoloxidase subunit 2-like
evm.TU.Herato0209.40	RNA-directed DNA polymerase from mobile element jockey-like
evm.TU.Herato0209.41	RNA-directed DNA polymerase from mobile element jockey-like
evm.TU.Herato0209.42	phenoloxidase subunit 2-like
evm.TU.Herato0214.23,novel_gene_22	rabphilin-3A isoform X1
evm.TU.Herato0215.146,evm.TU.Herato0215.147,evm.TU.Herato0215.9	esterase FE4-like
evm.TU.Herato0215.7	esterase FE4-like
evm.TU.Herato0215.8	piggyBac transposable element-derived protein 2-like
evm.TU.Herato0301.61,evm.TU.Herato0301.62,evm.TU.Herato0301.63,novel_gene_23	valacyclovir hydrolase-like
evm.TU.Herato0301.64,evm.TU.Herato0301.65	valacyclovir hydrolase
evm.TU.Herato0309.2	---NA---
evm.TU.Herato0310.1	hypothetical protein EVAR_58729_1
evm.TU.Herato0413.1	Retrovirus-related Pol polyprotein from transposon opus-like Protein
evm.TU.Herato0413.2	RNA-directed DNA polymerase from mobile element jockey
evm.TU.Herato0507.1	neuropeptide CCHamide-1 receptor
evm.TU.Herato0605.1	nucleic-acid-binding protein from mobile element jockey-like
evm.TU.Herato0606.237	uncharacterized protein LOC113398345 isoform X2
evm.TU.Herato0801.11	structural maintenance of chromosomes protein 2
evm.TU.Herato0801.12	phytanoyl-CoA dioxygenase, peroxisomal-like
evm.TU.Herato0801.13	uncharacterized protein LOC113397033
evm.TU.Herato0801.302	isopentenyl-diphosphate Delta-isomerase 1
evm.TU.Herato0801.303	kinesin heavy chain-like
evm.TU.Herato0801.87,evm.TU.Herato0801.88	protein doublesex isoform X1
evm.TU.Herato0807.1,evm.TU.Herato0807.2	Brain-specific angiogenesis inhibitor 1
evm.TU.Herato0821.63	uncharacterized protein LOC116773344
evm.TU.Herato0821.64	ribonuclease P protein subunit rpr2

evm.TU.Herato0821.65	polyribonucleotide nucleotidyltransferase 1, mitochondrial
evm.TU.Herato0821.66	3'-5' ssDNA/RNA exonuclease TatD
evm.TU.Herato0901.255	hypothetical protein OBRU01_15968
evm.TU.Herato0901.256	Retrovirus-related Pol polyprotein from transposon 412
evm.TU.Herato1001.161	WntA signaling ligand
evm.TU.Herato1103.1	zinc finger DNA binding protein
evm.TU.Herato1104.1	Zinc finger DNA binding protein
evm.TU.Herato1105.1	---NA---
evm.TU.Herato1108.384	nose resistant to fluoxetine protein 6-like
evm.TU.Herato1202.820	protein deltex
evm.TU.Herato1411.45	angiogenic factor with G patch and FHA domains 1 isoform X2
evm.TU.Herato1505.54	guanylate cyclase 32E
evm.TU.Herato1505.55	galactokinase-like
evm.TU.Herato1507.220	putative acetyltransferase
evm.TU.Herato1507.259,evm.TU.Herato1507.260,evm.TU.Herato1507.261,evm.TU.Herato1507.262	uncharacterized protein LOC117987370
evm.TU.Herato1507.347	esterase FE4-like
evm.TU.Herato1601.83	---NA---
evm.TU.Herato1603.1	Gag-like protein
evm.TU.Herato1701.353_evm.TU.Herato1701.354	lipopolysaccharide-induced tumor necrosis factor-alpha factor homolog
evm.TU.Herato1701.355	lipopolysaccharide-induced tumor necrosis factor-alpha factor homolog
evm.TU.Herato1701.356	lipopolysaccharide-induced tumor necrosis factor-alpha factor homolog
evm.TU.Herato1801.58,evm.TU.Herato1801.59,evm.TU.Herato1801.60	SCY1-like protein 2
evm.TU.Herato1801.61	leucine repeat-rich protein
evm.TU.Herato1801.62	leucine repeat-rich protein
evm.TU.Herato1801.63	integrator complex subunit 7
evm.TU.Herato1801.64	homeobox protein SIX6-like
evm.TU.Herato1801.65	epoxide hydrolase 4-like
evm.TU.Herato1801.66	adhesion G protein-coupled receptor A3
evm.TU.Herato1801.67	kinesin-like protein KIF20B
evm.TU.Herato1801.68	putative rabkinesin 6
evm.TU.Herato1801.69_evm.TU.Herato1801.70	pre-mRNA-splicing factor Slu7
evm.TU.Herato1801.71	sorting nexin-12
evm.TU.Herato1801.74	blood vessel epicardial substance-like
evm.TU.Herato1801.75	dnaJ homolog subfamily C member 25 homolog
evm.TU.Herato1801.76	DALR anticodon-binding domain-containing protein 3
evm.TU.Herato1801.77	max dimerization-like protein
evm.TU.Herato1801.78	craniofacial development protein 2-like
evm.TU.Herato1805.60,evm.TU.Herato1805.61,evm.TU.Herato1805.62	uncharacterized aarF domain-containing protein kinase 1
evm.TU.Herato1805.63	ubiquinone biosynthesis O-methyltransferase, mitochondrial-like
evm.TU.Herato1904.217	---NA---
evm.TU.Herato1904.218	fatty acyl-CoA reductase 1-like
evm.TU.Herato1904.222_evm.TU.Herato1904.223	putative fatty acyl-CoA reductase CG5065
evm.TU.Herato1904.224	Piezo-type mechanosensitive ion channel component
evm.TU.Herato1904.225	hypothetical protein EVAR_12794_1
evm.TU.Herato1904.226	hypothetical protein
evm.TU.Herato1904.230	glucose dehydrogenase [FAD, quinone]-like

evm.TU.Herato2101.292	Y+L amino acid transporter 2
evm.TU.Herato2101.293	uncharacterized protein LOC117995498
evm.TU.Herato2101.294	---NA---
evm.TU.Herato2101.295	---NA---
evm.TU.Herato2101.296	uncharacterized protein LOC117989496
evm.TU.Herato2101.392	uncharacterized protein LOC116777930 isoform X2
evm.TU.Herato2101.399	peptidoglycan recognition protein 1-like isoform X1

TableS3: genes withing Fst signal identified

sample name	organism	stage	wing	filtered reads	# peaks	FRiP score
E3_FW	H. e. demophoon	5th instar	forewing	1769739	14771	0.27
Not1_HW	H. e. notabilis	5th instar	hindwing	425629	5791	0.30
E_Not3_FW	H. e. notabilis	5th instar	hindwing	3647599	17706	0.46
LB_16	H. e. favorinus	5th instar	forewing	4807567	20963	0.52
LB_29	H. e. hydara	5th instar	forewing	5307641	22277	0.54
LB_27	H. e. hydara	5th instar	forewing	6788764	24672	0.54
LB_28	H. e. hydara	5th instar	hindwing	4960205	21625	0.57
LB_17	H. e. favorinus	5th instar	forewing	1696138	18796	0.29
Not2_FW	H. e. notabilis	5th instar	forewing	4763907	21362	0.42
Not1_FW	H. e. notabilis	5th instar	forewing	6229449	24050	0.50
LI7_demophoon_FW	H. e. demophoon	5th instar	hindwing	4075717	28539	0.37
LB_19	H. e. favorinus	5th instar	hindwing	6359448	21068	0.56
LB_21	H. e. hydara	5th instar	forewing	4383080	27916	0.29
LB_18	H. e. favorinus	5th instar	hindwing	5422264	24091	0.38
LB_30	H. e. hydara	5th instar	hindwing	10360642	27559	0.56
LI7_demophoon_HW	H. e. demophoon	5th instar	forewing	2438333	21901	0.24
LB_6	H. e. favorinus	5th instar	forewing	7763380	45087	0.41
LB_41	H. e. demophoon	5th instar	forewing	6924461	25476	0.45
E_ety2_FW	H. e. etylus	5th instar	hindwing	13046479	26273	0.69
Et4_HW	H. e. etylus	5th instar	hindwing	4631453	27294	0.28
LB_7	H. e. favorinus	5th instar	forewing	4460626	31410	0.31
Et4_FW	H. e. etylus	5th instar	hindwing	10151822	31012	0.52
LB_42	H. e. demophoon	5th instar	hindwing	8606606	30192	0.49
E_ety2_HW	H. e. etylus	5th instar	hindwing	13949150	21508	0.76
Not2_HW	H. e. notabilis	5th instar	hindwing	3611979	30479	0.24
E3_HW	H. e. demophoon	5th instar	hindwing	2923863	25027	0.21
E_Not3_HW	H. e. notabilis	5th instar	hindwing	10549824	11992	0.64
Chest2_HW	H. e. chest	5th instar	forewing	5340579	23635	0.39
Chest2_FW	H. e. chest	5th instar	forewing	12035516	34886	0.49
E_Ches1_FW	H. e. chest	5th instar	hindwing	12676179	30999	0.68
Et5_HW	H. e. etylus	5th instar	forewing	10904324	49179	0.40

LB_20	H. e. hydara	5th instar	forewing	15676858	81833	0.39
Et5_FW	H. e. etylus	5th instar	forewing	8833798	71982	0.28
FW-pboy	H. e. demophoon	5th instar	forewing	14092769	64209	0.42
E_Ches1_Hw	H. e. chest	5th instar	hindwing	18248292	52120	0.60

TableS4: Quality measumernet for ATAC-seq data used in the study

comparison	morph	Wing	total	shared peak	shared peak %	unique peak	unique peak %	Variable peaks	Variable peaks %
demophoon vs	*demophoon*	FW	15721	11003	69.99	124	0.79	4594	29.22
	hydara		14691	11003	74.90	117	0.80	3571	24.31
	demophoon	HW	14978	7696	51.38	161	1.07	7121	47.54
	hydara		14877	7696	51.73	144	0.97	7037	47.30
notabilis vs et	*notabilis*	FW	14877	7871	52.91	69	0.46	6937	46.63
	etylus		16722	7871	47.07	138	0.83	8713	52.11
	notabilis	HW	12216	5997	49.09	50	0.41	6169	50.50
	etylus		15655	5997	38.31	123	0.79	9535	60.91

Table S5: ATAC-seq numbers in hybrid zones

demophoon-hydara hybridzone

Chr	Start	End	Distance from TSS	gene ID	gene name
pan	1241506	1241884	36048	evm.model.Herato0101.16	putative ATP-dependent RNA helicase DHX35
pan	1241506	1241884	36048	evm.model.Herato0101.16	putative ATP-dependent RNA helicase DHX35
pan	2511606	2512631	1823	evm.model.Herato0101.32	WD repeat-containing protein 43
pan	2608280	2608738	1749	evm.model.Herato0101.34	uncharacterized protein LOC113404900 isoform X1
pan	2608280	2608738	1749	evm.model.Herato0101.34	uncharacterized protein LOC113404900 isoform X1
pan	2748279	2748528	17639	evm.model.Herato0101.39	protein enabled isoform X1
pan	2748279	2748528	17639	evm.model.Herato0101.39	protein enabled isoform X1
pan	6366710	6366962	28181	evm.model.Herato0101.116	hypothetical protein EVAR_74554_1
pan	7110891	7111366	-50860	evm.model.Herato0101.120	adhesion transmembrane protein
pan	10555853	10556334	-6031	evm.model.Herato0101.165	spherulin-2A-like
pan	10555853	10556334	-6031	evm.model.Herato0101.165	spherulin-2A-like
pan	11326660	11327160	5987	evm.model.Herato0101.188_evm.TU.Herato0101.189	uncharacterized protein LOC113392023
pan	11636324	11636866	41364	evm.model.Herato0101.199	---NA---
pan	11636324	11636866	41364	evm.model.Herato0101.199	---NA---
pan	12304831	12305318	-13754	evm.model.Herato0101.214	uncharacterized protein LOC115254287
pan	12304831	12305318	-13754	evm.model.Herato0101.214	uncharacterized protein LOC115254287
pan	13633886	13634100	-13361	evm.model.Herato0101.233	lipase 1-like
pan	13641039	13641747	-5961	evm.model.Herato0101.233	lipase 1-like
pan	13641039	13641747	-5961	evm.model.Herato0101.233	lipase 1-like
pan	19246250	19246712	6357	evm.model.Herato0101.315	carbohydrate sulfotransferase 11
pan	19246250	19246712	6357	evm.model.Herato0101.315	carbohydrate sulfotransferase 11
pan	19803749	19804139	-24067	evm.model.Herato0101.328	myosin-VIIa isoform X2
pan	19811956	19812548	-32375	evm.model.Herato0101.328	myosin-VIIa isoform X2
pan	19812271	19812736	-32626	evm.model.Herato0101.328	myosin-VIIa isoform X2

pan	19812271	19812736	-32626	evm.model.Herato0101.328	myosin-VIIa isoform X2
pan	22562146	22562365	11637	evm.model.Herato0101.454	putative Activating molecule in BECN1-regulated autophagy protein 1
pan	23671630	23672039	-1046	evm.model.Herato0101.494	chitin synthase chs-2-like
pan	23671630	23672039	-1046	evm.model.Herato0101.494	chitin synthase chs-2-like
pan	26527099	26527430	50594	evm.model.Herato0101.573	programmed cell death protein 10
pan	30814989	30815268	-2654	evm.model.Herato0101.726	integrin beta-6-like
pan	32861049	32861503	-2415	evm.model.Herato0101.762	lysosomal thioesterase PPT2 homolog
pan	32947001	32947704	53776	evm.model.Herato0101.764	defense protein 2
pan	35205254	35205514	6074	evm.model.Herato0101.801	chromobox protein homolog 5-like
pan	47526218	47526699	10029	evm.model.Herato0209.49	uncharacterized protein LOC113395223 isoform X1
pan	49470162	49470492	254	evm.model.Herato0211.19	gephyrin
pan	51808408	51808761	8318	evm.model.Herato0211.62	serine-rich adhesin for platelets-like isoform X1
pan	54414211	54414554	18211	evm.model.Herato0211.97	very-long-chain 3-oxoacyl-CoA reductase
pan	56168200	56168407	80081	evm.model.Herato0214.21	RILP protein
pan	56725004	56725550	-14583	evm.model.Herato0214.40	PAX-interacting protein 1-like
pan	57750752	57751494	-28201	evm.model.Herato0215.1	alpha-tocopherol transfer protein-like
pan	62335562	62335924	-15576	evm.model.Herato0215.81	hsp70-binding protein 1
pan	62335562	62335924	-15576	evm.model.Herato0215.81	hsp70-binding protein 1
pan	62846636	62847280	13033	evm.model.Herato0215.90	UPF0061 protein PFL_0486-like
pan	62846636	62847280	13033	evm.model.Herato0215.90	UPF0061 protein PFL_0486-like
pan	63593567	63593960	-8361	evm.model.Herato0215.117	required for meiotic nuclear division protein 1 homolog
pan	63653005	63653552	51154	evm.model.Herato0215.117	required for meiotic nuclear division protein 1 homolog
pan	66877779	66878193	-28283	evm.model.Herato0301.18	phenylalanine--tRNA ligase alpha subunit
pan	69956244	69957008	-156764	evm.model.Herato0301.46	ras-related protein Rap1
pan	71048936	71049273	-540	evm.model.Herato0301.71	RING finger and transmembrane domain-containing protein 2
pan	72024285	72024687	-38253	evm.model.Herato0301.93	sodium channel protein Nach-like

pan	73175222	73175617	45031	evm.model.Herato0301.104	vacuolar protein sorting-associated protein 72 homolog
pan	77123835	77124491	-35324	evm.model.Herato0310.11	DNA ligase 3
pan	78670499	78670934	100885	evm.model.Herato0310.24	---NA---
pan	81054520	81055002	-79536	evm.model.Herato0310.51	hypothetical protein
pan	81054548	81055003	-79550	evm.model.Herato0310.51	hypothetical protein
pan	87628124	87628485	7077	evm.model.Herato0310.157	transmembrane protease serine 9-like
pan	87636170	87636571	-2377	evm.model.Herato0310.158	transmembrane protease serine 9-like
pan	87636170	87636571	-2377	evm.model.Herato0310.158	transmembrane protease serine 9-like
pan	94181948	94182261	-21239	evm.model.Herato0310.290	synaptic vesicle glycoprotein 2B-like
pan	95540245	95540730	-154	evm.model.Herato0310.316	synaptic vesicle 2-related protein-like
pan	95540245	95540730	-154	evm.model.Herato0310.316	synaptic vesicle 2-related protein-like
pan	96380421	96380679	12137	evm.model.Herato0401.2	cytochrome P450 6B6-like
pan	96380421	96380679	12137	evm.model.Herato0401.2	cytochrome P450 6B6-like
pan	98093809	98094620	39669	evm.model.Herato0401.15	chondroitin sulfate synthase 1
pan	98093809	98094620	39669	evm.model.Herato0401.15	chondroitin sulfate synthase 1
pan	98154855	98155231	4033	evm.model.Herato0401.16	cuticular protein glycine-rich 20
pan	98154855	98155231	4033	evm.model.Herato0401.16	cuticular protein glycine-rich 20
pan	98743985	98744373	15289	evm.model.Herato0401.23	hypothetical protein RR46_05687
pan	1E+08	1E+08	78888	evm.model.Herato0401.50	heterogeneous nuclear ribonucleoprotein 27C isoform X4
pan	1.03E+08	1.03E+08	38071	evm.model.Herato0403.10	hypothetical protein EVAR_75896_1
pan	1.03E+08	1.03E+08	-126208	evm.model.Herato0403.11	secretory carrier-associated membrane protein 5B isoform X1
pan	1.04E+08	1.04E+08	94308	evm.model.Herato0404.1	furin-like protease 2
pan	1.04E+08	1.04E+08	94308	evm.model.Herato0404.1	furin-like protease 2
pan	1.04E+08	1.04E+08	398	evm.model.Herato0405.13	bumetanide-sensitive sodium-(potassium)-chloride cotransporter
pan	1.04E+08	1.04E+08	398	evm.model.Herato0405.13	bumetanide-sensitive sodium-(potassium)-chloride cotransporter
pan	1.04E+08	1.04E+08	3584	evm.model.Herato0405.14	40S ribosomal protein S15
pan	1.04E+08	1.04E+08	3580	evm.model.Herato0405.14	40S ribosomal protein S15

pan	1.06E+08	1.06E+08	-11438	evm.model.Herato0405.42	alpha-tocopherol transfer protein-like
pan	1.08E+08	1.08E+08	15332	evm.model.Herato0411.2	---NA---
pan	1.08E+08	1.08E+08	15326	evm.model.Herato0411.2	---NA---
pan	1.09E+08	1.09E+08	86143	evm.model.Herato0411.15	Gag protein
pan	1.29E+08	1.29E+08	-67680	evm.model.Herato0501.37	bone morphogenetic protein receptor type-1B isoform X1
pan	1.29E+08	1.29E+08	-58369	evm.model.Herato0501.38	60S ribosomal protein L34-like
pan	1.29E+08	1.29E+08	-58369	evm.model.Herato0501.38	60S ribosomal protein L34-like
pan	1.31E+08	1.31E+08	28300	evm.model.Herato0503.21	zinc finger-containing ubiquitin peptidase 1-like
pan	1.31E+08	1.31E+08	28300	evm.model.Herato0503.21	zinc finger-containing ubiquitin peptidase 1-like
pan	1.34E+08	1.34E+08	-2380	evm.model.Herato0503.85	uncharacterized protein LOC112048264
pan	1.35E+08	1.35E+08	3683	evm.model.Herato0503.96	SET and MYND domain-containing protein 4
pan	1.37E+08	1.37E+08	2115	evm.model.Herato0503.120	NFU1 iron-sulfur cluster scaffold homolog, mitochondrial-like
pan	1.37E+08	1.37E+08	-91249	evm.model.Herato0503.121	protein spaetzle 3
pan	1.37E+08	1.37E+08	-41708	evm.model.Herato0503.122	seminal fluid protein HACP011
pan	1.38E+08	1.38E+08	36585	evm.model.Herato0503.131	cGMP-dependent protein kinase, isozyme 2 forms cD5/T2-like isoform X1
pan	1.38E+08	1.38E+08	36585	evm.model.Herato0503.131	cGMP-dependent protein kinase, isozyme 2 forms cD5/T2-like isoform X1
pan	1.39E+08	1.39E+08	67480	evm.model.Herato0503.132	rap1 GTPase-activating protein 1 isoform X1
pan	1.42E+08	1.42E+08	-1030	evm.model.Herato0503.221	ADP-ribosylation factor 2
pan	1.42E+08	1.42E+08	-50081	evm.model.Herato0503.225	uncharacterized protein LOC113400287
pan	1.43E+08	1.43E+08	-20815	evm.model.Herato0503.241	estradiol 17-beta-dehydrogenase 11-like
pan	1.43E+08	1.43E+08	-1026	evm.model.Herato0503.241	estradiol 17-beta-dehydrogenase 11-like
pan	1.43E+08	1.43E+08	-1631	evm.model.Herato0503.250	potassium channel AKT3-like
pan	1.45E+08	1.45E+08	742	evm.model.Herato0503.286	uncharacterized protein LOC111354683
pan	1.45E+08	1.45E+08	748	evm.model.Herato0503.286	uncharacterized protein LOC111354683
pan	1.45E+08	1.45E+08	967	evm.model.Herato0503.287	uncharacterized protein LOC111354683
pan	1.45E+08	1.45E+08	8944	evm.model.Herato0503.287	uncharacterized protein LOC111354683

pan	1.45E+08	1.45E+08	8933	evm.model.Herato0503.287	uncharacterized protein LOC111354683
pan	1.45E+08	1.45E+08	-10491	evm.model.Herato0503.288	early boundary activity protein 2-like isoform X2
pan	1.48E+08	1.48E+08	-33852	evm.model.Herato0508.4	glyoxylate reductase/hydroxypyruvate reductase-like
pan	1.54E+08	1.54E+08	15281	evm.model.Herato0511.7	hypothetical protein B5V51_8296
pan	1.54E+08	1.54E+08	-17371	evm.model.Herato0511.8	potassium/sodium hyperpolarization-activated cyclic nucleotide-gated channel 4-like isoform X15
pan	1.54E+08	1.54E+08	-25023	evm.model.Herato0511.9	unnamed protein product
pan	1.66E+08	1.66E+08	-141200	evm.model.Herato0606.49	pyruvate kinase-like
pan	1.66E+08	1.66E+08	1321	evm.model.Herato0606.54	uncharacterized protein LOC117987079 isoform X3
pan	1.67E+08	1.67E+08	120	evm.model.Herato0606.58	dnaJ homolog subfamily C member 8
pan	1.69E+08	1.69E+08	-2682	evm.model.Herato0606.96	protein spindle-F
pan	1.7E+08	1.7E+08	-9094	evm.model.Herato0606.111	---NA---
pan	1.7E+08	1.7E+08	-9094	evm.model.Herato0606.111	---NA---
pan	1.7E+08	1.7E+08	-20961	evm.model.Herato0606.122	trypsin-1-like
pan	1.7E+08	1.7E+08	-20961	evm.model.Herato0606.122	trypsin-1-like
pan	1.7E+08	1.7E+08	-36387	evm.model.Herato0606.122	trypsin-1-like
pan	1.71E+08	1.71E+08	-19160	evm.model.Herato0606.132	barH-like 2 homeobox protein
pan	1.74E+08	1.74E+08	-486	evm.model.Herato0606.204	uncharacterized protein C15orf61 isoform X1
pan	1.76E+08	1.76E+08	37645	evm.model.Herato0606.240	Neuronal acetylcholine receptor subunit alpha-3
pan	1.79E+08	1.79E+08	26090	evm.model.Herato0606.284	uncharacterized protein LOC116766716 isoform X2
pan	1.8E+08	1.8E+08	-10412	evm.model.Herato0606.291	phospholipase B1, membrane-associated-like
pan	1.8E+08	1.8E+08	-10412	evm.model.Herato0606.291	phospholipase B1, membrane-associated-like
pan	1.8E+08	1.8E+08	350	evm.model.Herato0606.302	neurexin-4 isoform X1
pan	1.81E+08	1.81E+08	-158677	evm.model.Herato0606.306	thioredoxin-2
pan	1.81E+08	1.81E+08	-158671	evm.model.Herato0606.306	thioredoxin-2
pan	1.82E+08	1.82E+08	12679	evm.model.Herato0606.331	Copper chaperone for superoxide dismutase
pan	1.82E+08	1.82E+08	12679	evm.model.Herato0606.331	Copper chaperone for superoxide dismutase

pan	1.83E+08	1.83E+08	3878	evm.model.Herato0606.374	Replicase large subunit
pan	1.83E+08	1.83E+08	3878	evm.model.Herato0606.374	Replicase large subunit
pan	1.85E+08	1.85E+08	-18359	evm.model.Herato0606.452	uncharacterized protein LOC113391393
pan	1.87E+08	1.87E+08	2827	evm.model.Herato0608.5	zinc finger BED domain-containing protein 5-like
pan	1.87E+08	1.87E+08	2827	evm.model.Herato0608.5	zinc finger BED domain-containing protein 5-like
pan	1.89E+08	1.89E+08	38617	evm.model.Herato0609.84	unnamed protein product
pan	1.89E+08	1.89E+08	38617	evm.model.Herato0609.84	unnamed protein product
pan	1.9E+08	1.9E+08	9902	evm.model.Herato0609.90	unnamed protein product
pan	1.9E+08	1.9E+08	9922	evm.model.Herato0609.90	unnamed protein product
pan	1.92E+08	1.92E+08	5958	evm.model.Herato0701.46	pyruvate dehydrogenase (acetyl-transferring) kinase, mitochondrial
pan	1.96E+08	1.96E+08	-6567	evm.model.Herato0701.183	Disks large-associated protein 5
pan	1.96E+08	1.96E+08	-6567	evm.model.Herato0701.183	Disks large-associated protein 5
pan	1.99E+08	1.99E+08	-4135	evm.model.Herato0701.244	uncharacterized protein LOC112043869
pan	1.99E+08	1.99E+08	-4135	evm.model.Herato0701.244	uncharacterized protein LOC112043869
pan	2.01E+08	2.01E+08	-62103	evm.model.Herato0701.283	E3 ubiquitin-protein ligase ZNRF1
pan	2.01E+08	2.01E+08	-62103	evm.model.Herato0701.283	E3 ubiquitin-protein ligase ZNRF1
pan	2.01E+08	2.01E+08	82199	evm.model.Herato0701.284	E3 ubiquitin-protein ligase ZNRF1
pan	2.01E+08	2.01E+08	82199	evm.model.Herato0701.284	E3 ubiquitin-protein ligase ZNRF1
pan	2.01E+08	2.01E+08	-7417	evm.model.Herato0701.286	TATA element modulatory factor
pan	2.04E+08	2.04E+08	-82347	novel_exon_70	transposase
pan	2.06E+08	2.06E+08	36767	evm.model.Herato0701.400	supervillin-like isoform X4
pan	2.06E+08	2.06E+08	36767	evm.model.Herato0701.400	supervillin-like isoform X4
pan	2.08E+08	2.08E+08	1680	evm.model.Herato0701.446	anamorsin homolog
pan	2.09E+08	2.09E+08	-8832	evm.model.Herato0701.470	zinc finger CCCH domain-containing protein 18 isoform X1
pan	2.09E+08	2.09E+08	-16	evm.model.Herato0701.495	low-density lipoprotein receptor class A domain-containing protein 4 isoform X2
pan	2.09E+08	2.09E+08	-16	evm.model.Herato0701.495	low-density lipoprotein receptor class A domain-containing protein 4 isoform X2

pan	2.1E+08	2.1E+08	11445	evm.model.Herato0701.521	xylosyltransferase oxt
pan	2.1E+08	2.1E+08	16430	evm.model.Herato0701.539	putative Phosphatidylinositol 3-and 4-kinase
pan	2.11E+08	2.11E+08	-14886	evm.model.Herato0701.583	hypothetical protein EVAR_58729_1
pan	2.11E+08	2.11E+08	-14886	evm.model.Herato0701.583	hypothetical protein EVAR_58729_1
pan	2.18E+08	2.18E+08	-66657	evm.model.Herato0701.693	opsin
pan	2.2E+08	2.2E+08	-48747	evm.model.Herato0701.740_evm.TU.Herato0701.738	pre-mRNA-splicing factor ATP-dependent RNA helicase PRP16
pan	2.2E+08	2.2E+08	-48747	evm.model.Herato0701.740_evm.TU.Herato0701.738	pre-mRNA-splicing factor ATP-dependent RNA helicase PRP16
pan	2.22E+08	2.22E+08	-98	evm.model.Herato0701.757	longitudinals lacking protein-like isoform X1
pan	2.22E+08	2.22E+08	-98	evm.model.Herato0701.757	longitudinals lacking protein-like isoform X1
pan	2.22E+08	2.22E+08	-31030	evm.model.Herato0701.762	uncharacterized protein LOC113514709
pan	2.28E+08	2.28E+08	-500	evm.model.Herato0801.46	zinc transporter 7
pan	2.28E+08	2.28E+08	-500	evm.model.Herato0801.46	zinc transporter 7
pan	2.29E+08	2.29E+08	13396	evm.model.Herato0801.66	spastin isoform X3
pan	2.29E+08	2.29E+08	13396	evm.model.Herato0801.66	spastin isoform X3
pan	2.3E+08	2.3E+08	9270	evm.model.Herato0801.72	IQ domain-containing protein G-like
pan	2.32E+08	2.32E+08	23680	evm.model.Herato0801.101	chromosome-associated kinesin KIF4
pan	2.35E+08	2.35E+08	9263	evm.model.Herato0801.181	uncharacterized protein LOC117990688
pan	2.39E+08	2.39E+08	-706	evm.model.Herato0801.280	caspase-8
pan	2.42E+08	2.42E+08	-140427	evm.model.Herato0813.1	uncharacterized protein LOC115455229, partial
pan	2.42E+08	2.42E+08	-34003	evm.model.Herato0813.1	uncharacterized protein LOC115455229, partial
pan	2.47E+08	2.47E+08	-7274	evm.model.Herato0821.85	decaprenyl-diphosphate synthase subunit 2-like
pan	2.47E+08	2.47E+08	-7274	evm.model.Herato0821.85	decaprenyl-diphosphate synthase subunit 2-like
pan	2.49E+08	2.49E+08	-20158	evm.model.Herato0821.132	protein serrate
pan	2.49E+08	2.49E+08	-3903	novel_exon_96	RNA-directed DNA polymerase from mobile element jockey-like
pan	2.49E+08	2.49E+08	-3903	novel_exon_96	RNA-directed DNA polymerase from mobile element jockey-like
pan	2.58E+08	2.58E+08	51260	evm.model.Herato0901.104_evm.TU.Herato0901.106	39S ribosomal protein L19, mitochondrial

pan	2.66E+08	2.66E+08	-15141	evm.model.Herato0901.275	trithorax group protein osa isoform X5
pan	2.66E+08	2.66E+08	-15141	evm.model.Herato0901.275	trithorax group protein osa isoform X5
pan	2.67E+08	2.67E+08	-19596	evm.model.Herato0901.280	another transcription unit protein
pan	2.7E+08	2.7E+08	44475	evm.model.Herato0901.324	dystrophin, isoforms A/C/F/G/H-like
pan	2.73E+08	2.73E+08	-104127	evm.model.Herato0901.372	neural cell adhesion molecule 1-like isoform X2
pan	2.75E+08	2.75E+08	-1658	evm.model.Herato0904.20	endonuclease-reverse transcriptase
pan	2.75E+08	2.75E+08	-1658	evm.model.Herato0904.20	endonuclease-reverse transcriptase
pan	2.75E+08	2.75E+08	-58398	evm.model.Herato0904.25	UPF0160 protein MYG1, mitochondrial isoform X2
pan	2.76E+08	2.76E+08	-15188	evm.model.Herato1001.23	---NA---
pan	2.76E+08	2.76E+08	-8977	evm.model.Herato1001.26	tektin-4-like
pan	2.76E+08	2.76E+08	30687	evm.model.Herato1001.27	ribosomal protein S6 kinase delta-1-like isoform X2
pan	2.76E+08	2.76E+08	33212	evm.model.Herato1001.39	facilitated trehalose transporter Tret1-like
pan	2.76E+08	2.76E+08	33212	evm.model.Herato1001.39	facilitated trehalose transporter Tret1-like
pan	2.8E+08	2.8E+08	-26098	evm.model.Herato1001.106	rho guanine nucleotide exchange factor 18 isoform X4
pan	2.87E+08	2.87E+08	-1130	evm.model.Herato1003.9	serine protease inhibitor 2.1-like
pan	2.87E+08	2.87E+08	-1130	evm.model.Herato1003.9	serine protease inhibitor 2.1-like
pan	2.88E+08	2.88E+08	-18403	evm.model.Herato1003.23	zinc transporter 2-like
pan	2.88E+08	2.88E+08	-703	evm.model.Herato1003.31	chymotrypsin-like elastase family member 2A
pan	2.88E+08	2.88E+08	-703	evm.model.Herato1003.31	chymotrypsin-like elastase family member 2A
pan	2.9E+08	2.9E+08	-565	evm.model.Herato1003.65	chymotrypsin-like elastase family member 2A
pan	2.9E+08	2.9E+08	-565	evm.model.Herato1003.65	chymotrypsin-like elastase family member 2A
pan	2.9E+08	2.9E+08	17234	evm.model.Herato1003.66	chymotrypsin-like elastase family member 2A
pan	2.94E+08	2.94E+08	53589	evm.model.Herato1003.114	reverse transcriptase
pan	2.94E+08	2.94E+08	53589	evm.model.Herato1003.114	reverse transcriptase
pan	2.96E+08	2.96E+08	16996	evm.model.Herato1003.171	ras-related protein Rab6 isoform X1
pan	2.96E+08	2.96E+08	16996	evm.model.Herato1003.171	ras-related protein Rab6 isoform X1
pan	2.99E+08	2.99E+08	8432	evm.model.Herato1003.265	UPF0489 protein C5orf22 homolog

pan	2.99E+08	2.99E+08	8416	evm.model.Herato1003.265	UPF0489 protein C5orf22 homolog
pan	3.01E+08	3.01E+08	-68	novel_exon_116	
pan	3.02E+08	3.02E+08	-5959	evm.model.Herato1005.84	agrin-like
pan	3.02E+08	3.02E+08	-5959	evm.model.Herato1005.84	agrin-like
pan	3.06E+08	3.06E+08	5124	evm.model.Herato1005.202	Kinetochore-associated protein 1
pan	3.06E+08	3.06E+08	5087	evm.model.Herato1005.202	Kinetochore-associated protein 1
pan	3.1E+08	3.1E+08	64787	evm.model.Herato1007.84	chitin deacetylase 5 isoform X6
pan	3.1E+08	3.1E+08	64792	evm.model.Herato1007.84	chitin deacetylase 5 isoform X6
pan	3.13E+08	3.13E+08	-29584	evm.model.Herato1007.146	uncharacterized LOC106124082
pan	3.14E+08	3.14E+08	22072	evm.model.Herato1007.171	immunoglobulin-binding protein 1
pan	3.14E+08	3.14E+08	22072	evm.model.Herato1007.171	immunoglobulin-binding protein 1
pan	3.14E+08	3.14E+08	24561	evm.model.Herato1007.182	uncharacterized MFS-type transporter C09D4.1 isoform X2
pan	3.14E+08	3.14E+08	-136	evm.model.Herato1007.183	obscurin isoform X3
pan	3.27E+08	3.27E+08	2915	evm.model.Herato1108.138	steroid receptor RNA activator 1-like
pan	3.28E+08	3.28E+08	67770	evm.model.Herato1108.153	YTH domain-containing family protein 3 isoform X1
pan	3.3E+08	3.3E+08	-115249	evm.model.Herato1108.241	filamin-A isoform X1
pan	3.3E+08	3.3E+08	-115249	evm.model.Herato1108.241	filamin-A isoform X1
pan	3.32E+08	3.32E+08	-66	evm.model.Herato1108.271	transcription termination factor 5, mitochondrial-like
pan	3.32E+08	3.32E+08	-66	evm.model.Herato1108.271	transcription termination factor 5, mitochondrial-like
pan	3.37E+08	3.37E+08	781	evm.model.Herato1108.386	---NA---
pan	3.37E+08	3.37E+08	-9668	evm.model.Herato1108.396	hypothetical protein RR48_11784
pan	3.37E+08	3.37E+08	-9668	evm.model.Herato1108.396	hypothetical protein RR48_11784
pan	3.38E+08	3.38E+08	-2064	evm.model.Herato1108.406	UPF0676 protein C1494.01-like
pan	3.38E+08	3.38E+08	-2064	evm.model.Herato1108.406	UPF0676 protein C1494.01-like
pan	3.38E+08	3.38E+08	-349	evm.model.Herato1108.414	general transcription factor 3C polypeptide 6-like isoform X2
pan	3.39E+08	3.39E+08	26212	evm.model.Herato1108.449	probable Ras GTPase-activating protein isoform X6
pan	3.46E+08	3.46E+08	47235	evm.model.Herato1108.603	protein crumbs
pan	3.46E+08	3.46E+08	47235	evm.model.Herato1108.603	protein crumbs

pan	3.49E+08	3.49E+08	56287	evm.model.Herato1201.11	transmembrane protease serine 11G-like
pan	3.5E+08	3.5E+08	11370	evm.model.Herato1202.25	serine protease 55-like
pan	3.5E+08	3.5E+08	11370	evm.model.Herato1202.25	serine protease 55-like
pan	3.52E+08	3.52E+08	-2476	evm.model.Herato1202.59	ethanolaminephosphotransferase 1-like
pan	3.52E+08	3.52E+08	10765	evm.model.Herato1202.60	partitioning defective protein 6
pan	3.52E+08	3.52E+08	10765	evm.model.Herato1202.60	partitioning defective protein 6
pan	3.53E+08	3.53E+08	-679	evm.model.Herato1202.63	putative kakapo
pan	3.53E+08	3.53E+08	167189	novel_exon_140	Transposon Ty3-G Gag-Pol polyprotein
pan	3.54E+08	3.54E+08	-75263	evm.model.Herato1202.86	trypsin-5-like isoform X1
pan	3.56E+08	3.56E+08	-2281	evm.model.Herato1202.123	focal adhesion kinase 1 isoform X1
pan	3.56E+08	3.56E+08	-2281	evm.model.Herato1202.123	focal adhesion kinase 1 isoform X1
pan	3.56E+08	3.56E+08	6	evm.model.Herato1202.130	probable sodium-coupled neutral amino acid transporter 6
pan	3.57E+08	3.57E+08	5810	evm.model.Herato1202.151	phosphoenolpyruvate carboxykinase [GTP]-like isoform X1
pan	3.59E+08	3.59E+08	-46118	evm.model.Herato1202.216	sodium- and chloride-dependent transporter XTRP3 isoform X1
pan	3.59E+08	3.59E+08	-46118	evm.model.Herato1202.216	sodium- and chloride-dependent transporter XTRP3 isoform X1
pan	3.59E+08	3.59E+08	-14594	evm.model.Herato1202.225	GMP reductase 1-like
pan	3.59E+08	3.59E+08	-14594	evm.model.Herato1202.225	GMP reductase 1-like
pan	3.59E+08	3.59E+08	6822	evm.model.Herato1202.227	protein draper-like isoform X1
pan	3.59E+08	3.59E+08	-19960	evm.model.Herato1202.227	protein draper-like isoform X1
pan	3.59E+08	3.59E+08	-19960	evm.model.Herato1202.227	protein draper-like isoform X1
pan	3.59E+08	3.59E+08	-3870	evm.model.Herato1202.247	KH domain-containing, RNA-binding, signal transduction-associated protein 2-like isoform X1
pan	3.59E+08	3.59E+08	-3870	evm.model.Herato1202.247	KH domain-containing, RNA-binding, signal transduction-associated protein 2-like isoform X1
pan	3.67E+08	3.67E+08	54898	evm.model.Herato1202.407	calmodulin-binding transcription activator 2
pan	3.67E+08	3.67E+08	54913	evm.model.Herato1202.407	calmodulin-binding transcription activator 2
pan	3.69E+08	3.69E+08	10236	evm.model.Herato1202.439	COP9 signalosome complex subunit 7b

pan	3.7E+08	3.7E+08	-31585	evm.model.Herato1202.447	BMP and activin membrane-bound inhibitor homolog
pan	3.71E+08	3.71E+08	-7284	evm.model.Herato1202.464	dynactin subunit 5
pan	3.71E+08	3.71E+08	-7284	evm.model.Herato1202.464	dynactin subunit 5
pan	3.75E+08	3.75E+08	-14373	evm.model.Herato1202.553	LDLR chaperone boca
pan	3.76E+08	3.76E+08	18221	evm.model.Herato1202.573	exonuclease GOR-like isoform X1
pan	3.76E+08	3.76E+08	18221	evm.model.Herato1202.573	exonuclease GOR-like isoform X1
pan	3.77E+08	3.77E+08	525	evm.model.Herato1202.592	derlin-2
pan	3.77E+08	3.77E+08	-2223	evm.model.Herato1202.596	clustered mitochondria protein homolog isoform X1
pan	3.77E+08	3.77E+08	-2724	evm.model.Herato1202.600	DNA-directed RNA polymerase II 16 kDa polypeptide
pan	3.77E+08	3.77E+08	-2724	evm.model.Herato1202.600	DNA-directed RNA polymerase II 16 kDa polypeptide
pan	3.78E+08	3.78E+08	9602	evm.model.Herato1202.621	EF-hand domain-containing family member C2-like
pan	3.78E+08	3.78E+08	9602	evm.model.Herato1202.621	EF-hand domain-containing family member C2-like
pan	3.79E+08	3.79E+08	-46737	evm.model.Herato1202.659	---NA---
pan	3.79E+08	3.79E+08	-46737	evm.model.Herato1202.659	---NA---
pan	3.79E+08	3.79E+08	4704	evm.model.Herato1202.684	uncharacterized protein LOC117988862
pan	3.8E+08	3.8E+08	75260	evm.model.Herato1202.713	polyhomeotic-like protein 3 isoform X1
pan	3.81E+08	3.81E+08	15692	evm.model.Herato1202.729	hemicentin-1 isoform X1
pan	3.81E+08	3.81E+08	5538	evm.model.Herato1202.739	protein deadpan
pan	3.84E+08	3.84E+08	-11738	evm.model.Herato1202.790	amyloid protein-binding protein 2
pan	3.84E+08	3.84E+08	-11738	evm.model.Herato1202.790	amyloid protein-binding protein 2
pan	3.85E+08	3.85E+08	121127	evm.model.Herato1202.824	glucose dehydrogenase [FAD, quinone]-like
pan	3.87E+08	3.87E+08	-139649	evm.model.Herato1301.28	neuropeptide FF receptor 1-like isoform X1
pan	3.89E+08	3.89E+08	-219	evm.model.Herato1301.52	uncharacterized protein LOC113396369
pan	3.89E+08	3.89E+08	-219	evm.model.Herato1301.52	uncharacterized protein LOC113396369
pan	3.89E+08	3.89E+08	-2077	evm.model.Herato1301.52	uncharacterized protein LOC113396369
pan	3.89E+08	3.89E+08	-2077	evm.model.Herato1301.52	uncharacterized protein LOC113396369

pan	3.9E+08	3.9E+08	-7570	evm.model.Herato1301.57	elongation of very long chain fatty acids protein 6
pan	3.9E+08	3.9E+08	-100259	evm.model.Herato1301.64	serine/threonine-protein kinase DDB_G0283821
pan	3.91E+08	3.91E+08	-36338	evm.model.Herato1301.72	uncharacterized protein LOC116765631
pan	3.91E+08	3.91E+08	-36422	evm.model.Herato1301.72	uncharacterized protein LOC116765631
pan	3.92E+08	3.92E+08	19983	evm.model.Herato1301.97	hypothetical protein KGM_210035
pan	3.94E+08	3.94E+08	-476	evm.model.Herato1301.126	---NA---
pan	3.97E+08	3.97E+08	-4866	evm.model.Herato1301.221	mucin-5AC-like
pan	4.04E+08	4.04E+08	2156	evm.model.Herato1301.423	probable RNA-binding protein 46
pan	4.06E+08	4.06E+08	-21349	evm.model.Herato1301.474	ADP-ribosylation factor-like protein 8
pan	4.06E+08	4.06E+08	-6530	evm.model.Herato1301.474	ADP-ribosylation factor-like protein 8
pan	4.07E+08	4.07E+08	-21189	evm.model.Herato1301.483	elongation of very long chain fatty acids protein 7-like
pan	4.1E+08	4.1E+08	99232	evm.model.Herato1301.535	pseudouridylate synthase 7 homolog
pan	4.1E+08	4.1E+08	99232	evm.model.Herato1301.535	pseudouridylate synthase 7 homolog
pan	4.11E+08	4.11E+08	49174	evm.model.Herato1301.557	neurobeachin isoform X1
pan	4.14E+08	4.14E+08	5752	evm.model.Herato1301.596	adiponectin receptor protein
pan	4.15E+08	4.15E+08	17866	evm.model.Herato1301.606	hypothetical protein evm_004063
pan	4.2E+08	4.2E+08	2198	evm.model.Herato1301.692	protein mini spindles isoform X1
pan	4.21E+08	4.21E+08	679	evm.model.Herato1301.719	uncharacterized protein LOC113396606 isoform X3
pan	4.21E+08	4.21E+08	-16177	evm.model.Herato1301.721	---NA---
pan	4.21E+08	4.21E+08	-16177	evm.model.Herato1301.721	---NA---
pan	4.21E+08	4.21E+08	-6569	evm.model.Herato1301.721	---NA---
pan	4.21E+08	4.21E+08	-23474	evm.model.Herato1301.722	cyclin-dependent kinase inhibitor 1-like
pan	4.21E+08	4.21E+08	-23474	evm.model.Herato1301.722	cyclin-dependent kinase inhibitor 1-like
pan	4.25E+08	4.25E+08	-155	evm.model.Herato1301.780	protein cramped
pan	4.28E+08	4.28E+08	-39428	evm.model.Herato1408.13	E3 ubiquitin-protein ligase AMFR-like
pan	4.28E+08	4.28E+08	-39428	evm.model.Herato1408.13	E3 ubiquitin-protein ligase AMFR-like
pan	4.28E+08	4.28E+08	30888	evm.model.Herato1408.23	cholinesterase 2-like

pan	4.28E+08	4.28E+08	-235217	evm.model.Herato1408.26	transcription factor Sox-12-like
pan	4.28E+08	4.28E+08	-235217	evm.model.Herato1408.26	transcription factor Sox-12-like
pan	4.28E+08	4.28E+08	-229636	evm.model.Herato1408.26	transcription factor Sox-12-like
pan	4.28E+08	4.28E+08	-229636	evm.model.Herato1408.26	transcription factor Sox-12-like
pan	4.3E+08	4.3E+08	6034	evm.model.Herato1408.31	serine/threonine-protein kinase MARK2-like
pan	4.3E+08	4.3E+08	-41974	evm.model.Herato1408.55	oxysterol-binding protein-related protein 9
pan	4.3E+08	4.3E+08	-41974	evm.model.Herato1408.55	oxysterol-binding protein-related protein 9
pan	4.33E+08	4.33E+08	-6288	evm.model.Herato1408.85	probable beta-hexosaminidase fdl
pan	4.33E+08	4.33E+08	-6288	evm.model.Herato1408.85	probable beta-hexosaminidase fdl
pan	4.38E+08	4.38E+08	-24119	evm.model.Herato1408.228	glutamyl-tRNA(Gln) amidotransferase subunit B, mitochondrial
pan	4.38E+08	4.38E+08	-24119	evm.model.Herato1408.228	glutamyl-tRNA(Gln) amidotransferase subunit B, mitochondrial
pan	4.39E+08	4.39E+08	52752	novel_exon_163	uncharacterized protein LOC113403206
pan	4.39E+08	4.39E+08	52606	novel_exon_163	uncharacterized protein LOC113403206
pan	4.4E+08	4.4E+08	10914	evm.model.Herato1411.14	RING finger protein nhl-1-like
pan	4.43E+08	4.43E+08	-8754	evm.model.Herato1411.56	potassium/sodium hyperpolarization-activated cyclic nucleotide-gated channel 2 isoform X1
pan	4.43E+08	4.43E+08	-8754	evm.model.Herato1411.56	potassium/sodium hyperpolarization-activated cyclic nucleotide-gated channel 2 isoform X1
pan	4.45E+08	4.45E+08	18302	evm.model.Herato1411.123	protein FAM91A1
pan	4.47E+08	4.47E+08	3589	evm.model.Herato1411.170	hemocyte protein-glutamine gamma-glutamyltransferase-like
pan	4.48E+08	4.48E+08	196	evm.model.Herato1411.178	phospholipid phosphatase 2-like isoform X1
pan	4.5E+08	4.5E+08	15645	evm.model.Herato1411.196	pregnancy zone protein-like
pan	4.5E+08	4.5E+08	10368	evm.model.Herato1411.199	pregnancy zone protein-like
pan	4.5E+08	4.5E+08	10368	evm.model.Herato1411.199	pregnancy zone protein-like

pan	4.51E+08	4.51E+08	46707	evm.model.Herato1411.214_evm.TU.Herato1411.216_evm.TU.Herato1411.214_evm.TU.Herato1411.215	nose resistant to fluoxetine protein 6-like
pan	4.52E+08	4.52E+08	-20544	evm.model.Herato1411.231	endonuclease-reverse transcriptase
pan	4.53E+08	4.53E+08	-12243	evm.model.Herato1411.254	solute carrier family 22 member 1-like
pan	4.53E+08	4.53E+08	-12243	evm.model.Herato1411.254	solute carrier family 22 member 1-like
pan	4.58E+08	4.58E+08	208876	evm.model.Herato1505.86	poly(A)-specific ribonuclease PARN-like isoform X1
pan	4.58E+08	4.58E+08	208876	evm.model.Herato1505.86	poly(A)-specific ribonuclease PARN-like isoform X1
pan	4.58E+08	4.58E+08	21031	evm.model.Herato1505.86	poly(A)-specific ribonuclease PARN-like isoform X1
pan	4.59E+08	4.59E+08	-11	evm.model.Herato1505.100	protein penguin
pan	4.59E+08	4.59E+08	-11	evm.model.Herato1505.100	protein penguin
pan	4.6E+08	4.6E+08	19245	evm.model.Herato1505.131	RNA-directed DNA polymerase from mobile element jockey
pan	4.6E+08	4.6E+08	-18023	evm.model.Herato1505.133	protein FAM234A
pan	4.65E+08	4.65E+08	11691	evm.model.Herato1507.46	Protein pangolin, isoforms A/H/I
pan	4.69E+08	4.69E+08	-11630	evm.model.Herato1507.135	alpha-(1,3)-fucosyltransferase 6-like isoform X1
pan	4.72E+08	4.72E+08	-5632	evm.model.Herato1507.159	uncharacterized protein LOC110377276
pan	4.73E+08	4.73E+08	17394	evm.model.Herato1507.187	uncharacterized protein LOC113394940
pan	4.73E+08	4.73E+08	17394	evm.model.Herato1507.187	uncharacterized protein LOC113394940
pan	4.78E+08	4.78E+08	-64886	evm.model.Herato1507.262	uncharacterized protein LOC113402867
pan	4.78E+08	4.78E+08	-64886	evm.model.Herato1507.262	uncharacterized protein LOC113402867
pan	4.78E+08	4.78E+08	-3995	evm.model.Herato1507.272	E3 ubiquitin-protein ligase RING1
pan	4.78E+08	4.78E+08	-26188	evm.model.Herato1507.272	E3 ubiquitin-protein ligase RING1
pan	4.79E+08	4.79E+08	1600	evm.model.Herato1507.291	vacuolar protein sorting-associated protein 18 homolog
pan	4.88E+08	4.88E+08	11649	evm.model.Herato1524.19	---NA---
pan	4.89E+08	4.89E+08	-20090	evm.model.Herato1601.22	general transcriptional corepressor trfA isoform X1
pan	4.89E+08	4.89E+08	-20090	evm.model.Herato1601.22	general transcriptional corepressor trfA isoform X1
pan	4.89E+08	4.89E+08	-27042	evm.model.Herato1601.22	general transcriptional corepressor trfA isoform X1

pan	5E+08	5E+08	-101664	evm.model.Herato1602.9	G-protein coupled receptor moody-like
pan	5.01E+08	5.01E+08	1591	evm.model.Herato1603.20	xaa-Pro dipeptidase
pan	5.07E+08	5.07E+08	2421	evm.model.Herato1605.14	diphthine--ammonia ligase
pan	5.09E+08	5.09E+08	-18545	evm.model.Herato1605.35	inositol-trisphosphate 3-kinase B isoform X3
pan	5.09E+08	5.09E+08	7494	evm.model.Herato1605.36	inositol-trisphosphate 3-kinase A isoform X1
pan	5.09E+08	5.09E+08	3344	evm.model.Herato1605.40	unnamed protein product
pan	5.09E+08	5.09E+08	3188	evm.model.Herato1605.40	unnamed protein product
pan	5.09E+08	5.09E+08	-10422	evm.model.Herato1605.40	unnamed protein product
pan	5.11E+08	5.11E+08	6412	evm.model.Herato1605.174	uncharacterized protein LOC110991711
pan	5.11E+08	5.11E+08	38760	evm.model.Herato1605.65	disheveled-associated activator of morphogenesis 1 isoform X1
pan	5.11E+08	5.11E+08	38760	evm.model.Herato1605.65	disheveled-associated activator of morphogenesis 1 isoform X1
pan	5.11E+08	5.11E+08	5395	evm.model.Herato1605.84	proline-rich protein 12-like
pan	5.11E+08	5.11E+08	-32853	evm.model.Herato1605.84	proline-rich protein 12-like
pan	5.11E+08	5.11E+08	-47204	evm.model.Herato1605.84	proline-rich protein 12-like
pan	5.15E+08	5.15E+08	-13216	evm.model.Herato1605.131	uncharacterized protein LOC113402104 isoform X1
pan	5.16E+08	5.16E+08	9927	evm.model.Herato1605.141	symplekin
pan	5.16E+08	5.16E+08	9840	evm.model.Herato1605.141	symplekin
pan	5.17E+08	5.17E+08	36528	evm.model.Herato1701.18	spondin-1
pan	5.17E+08	5.17E+08	36528	evm.model.Herato1701.18	spondin-1
pan	5.18E+08	5.18E+08	-339539	evm.model.Herato1701.36	teneurin-m isoform X1
pan	5.18E+08	5.18E+08	-339539	evm.model.Herato1701.36	teneurin-m isoform X1
pan	5.18E+08	5.18E+08	-155223	evm.model.Herato1701.36	teneurin-m isoform X1
pan	5.18E+08	5.18E+08	-155223	evm.model.Herato1701.36	teneurin-m isoform X1
pan	5.19E+08	5.19E+08	-38879	evm.model.Herato1701.36	teneurin-m isoform X1
pan	5.19E+08	5.19E+08	-38879	evm.model.Herato1701.36	teneurin-m isoform X1
pan	5.25E+08	5.25E+08	-29201	evm.model.Herato1701.114	flocculation protein FLO11 isoform X1
pan	5.26E+08	5.26E+08	8479	evm.model.Herato1701.125	MIF-like protein mif-2

pan	5.26E+08	5.26E+08	8479	evm.model.Herato1701.125	MIF-like protein mif-2
pan	5.28E+08	5.28E+08	1864	evm.model.Herato1701.180	TIP41-like protein
pan	5.28E+08	5.28E+08	1864	evm.model.Herato1701.180	TIP41-like protein
pan	5.28E+08	5.28E+08	-18871	evm.model.Herato1701.183	gamma-tubulin complex component 2-like isoform X2
pan	5.28E+08	5.28E+08	22789	evm.model.Herato1701.184	uncharacterized protein LOC110997031
pan	5.28E+08	5.28E+08	22789	evm.model.Herato1701.184	uncharacterized protein LOC110997031
pan	5.28E+08	5.28E+08	-3359	evm.model.Herato1701.186	single-stranded DNA-binding protein, mitochondrial
pan	5.3E+08	5.3E+08	2417	evm.model.Herato1701.227	Macrophage MHC class 1 receptor 2 protein
pan	5.3E+08	5.3E+08	2417	evm.model.Herato1701.227	Macrophage MHC class 1 receptor 2 protein
pan	5.31E+08	5.31E+08	-3700	evm.model.Herato1701.248	LIM domain only protein 3-like
pan	5.31E+08	5.31E+08	-3700	evm.model.Herato1701.248	LIM domain only protein 3-like
pan	5.33E+08	5.33E+08	-18678	evm.model.Herato1701.307	cytoplasmic dynein 2 heavy chain 1
pan	5.37E+08	5.37E+08	123999	evm.model.Herato1703.2	tyrosine-protein phosphatase Lar-like isoform X1
pan	5.38E+08	5.38E+08	-157	evm.model.Herato1703.13	CREB-binding protein-like
pan	5.38E+08	5.38E+08	-157	evm.model.Herato1703.13	CREB-binding protein-like
pan	5.39E+08	5.39E+08	14549	evm.model.Herato1703.47	---NA---
pan	5.39E+08	5.39E+08	14549	evm.model.Herato1703.47	---NA---
pan	5.44E+08	5.44E+08	111	evm.model.Herato1705.19	leucine-rich repeat serine/threonine-protein kinase 1
pan	5.44E+08	5.44E+08	111	evm.model.Herato1705.19	leucine-rich repeat serine/threonine-protein kinase 1
pan	5.46E+08	5.46E+08	164	evm.model.Herato1705.97	DNA-directed RNA polymerases I, II, and III subunit RPABC3
pan	5.48E+08	5.48E+08	-15301	evm.model.Herato1708.12	dynactin subunit 1
pan	5.51E+08	5.51E+08	-5811	evm.model.Herato1708.126	phenoloxidase-activating factor 2-like
pan	5.58E+08	5.58E+08	-3336	evm.model.Herato1801.127	Kv channel-interacting protein 4-like
pan	5.6E+08	5.6E+08	13128	evm.model.Herato1801.164	---NA---
pan	5.68E+08	5.68E+08	-69501	evm.model.Herato1805.88	fatty acid synthase-like
pan	5.68E+08	5.68E+08	-69501	evm.model.Herato1805.88	fatty acid synthase-like
pan	5.69E+08	5.69E+08	-36359	evm.model.Herato1805.102	circadian clock-controlled protein-like

pan	5.72E+08	5.72E+08	-415	evm.model.Herato1805.135	axoneme-associated protein mst101(2) isoform X1
pan	5.72E+08	5.72E+08	-415	evm.model.Herato1805.135	axoneme-associated protein mst101(2) isoform X1
pan	5.75E+08	5.75E+08	-20368	evm.model.Herato1805.220	organic cation transporter protein-like
pan	5.76E+08	5.76E+08	-111627	evm.model.Herato1805.227	Down syndrome cell adhesion molecule-like protein 1 homolog
pan	5.76E+08	5.76E+08	-111585	evm.model.Herato1805.227	Down syndrome cell adhesion molecule-like protein 1 homolog
pan	5.77E+08	5.77E+08	-20644	evm.model.Herato1805.249	glycine receptor subunit alpha-2-like
pan	5.77E+08	5.77E+08	-20644	evm.model.Herato1805.249	glycine receptor subunit alpha-2-like
pan	5.79E+08	5.79E+08	-56668	evm.model.Herato1805.290	seminal fluid protein HACP027
pan	5.8E+08	5.8E+08	12657	evm.model.Herato1807.5	Tyrosine-protein phosphatase 99A
pan	5.8E+08	5.8E+08	12657	evm.model.Herato1807.5	Tyrosine-protein phosphatase 99A
pan	5.86E+08	5.86E+08	7785	evm.model.Herato1807.83	---NA---
pan	5.86E+08	5.86E+08	7785	evm.model.Herato1807.83	---NA---
pan	5.89E+08	5.89E+08	17710	evm.model.Herato1901.15	uncharacterized protein LOC113403184
pan	5.89E+08	5.89E+08	-17226	evm.model.Herato1901.17	putative lymphocyte cytosolic protein 2
pan	5.94E+08	5.94E+08	15325	evm.model.Herato1901.91	uncharacterized protein LOC113398113
pan	5.94E+08	5.94E+08	15325	evm.model.Herato1901.91	uncharacterized protein LOC113398113
pan	5.96E+08	5.96E+08	-164	evm.model.Herato1903.5	serine/threonine-protein kinase pelle
pan	5.98E+08	5.98E+08	-36632	evm.model.Herato1904.42	vesicular integral-membrane protein VIP36
pan	5.99E+08	5.99E+08	-42940	evm.model.Herato1904.61	disintegrin and metalloproteinase domain-containing protein 12
pan	6.03E+08	6.03E+08	-2069	evm.model.Herato1904.138	28S ribosomal protein S18c, mitochondrial
pan	6.03E+08	6.03E+08	2920	evm.model.Herato1904.147	TBC1 domain family member 7
pan	6.05E+08	6.05E+08	268	evm.model.Herato1904.204	UBX domain-containing protein 1
pan	6.05E+08	6.05E+08	268	evm.model.Herato1904.204	UBX domain-containing protein 1
pan	6.06E+08	6.06E+08	-7735	evm.model.Herato1904.213	excitatory amino acid transporter isoform X2
pan	6.07E+08	6.07E+08	75318	evm.model.Herato1904.238	hypothetical protein EVAR_56129_1
pan	6.19E+08	6.19E+08	-11732	evm.model.Herato1908.103	probable salivary secreted peptide

pan	6.19E+08	6.19E+08	1557	evm.model.Herato1910.19	RNA polymerase II transcriptional coactivator
pan	6.19E+08	6.19E+08	1557	evm.model.Herato1910.19	RNA polymerase II transcriptional coactivator
pan	6.2E+08	6.2E+08	21223	evm.model.Herato1910.71	esterase B1-like isoform X1
pan	6.21E+08	6.21E+08	-2536	evm.model.Herato1910.82	phospholipase A1-like
pan	6.22E+08	6.22E+08	50688	evm.model.Herato1910.116	Beat-IIIc isoform A
pan	6.22E+08	6.22E+08	50688	evm.model.Herato1910.116	Beat-IIIc isoform A
pan	6.25E+08	6.25E+08	155285	evm.model.Herato1910.159	kinesin-like protein CG14535 isoform X2
pan	6.26E+08	6.26E+08	5176	evm.model.Herato1910.175	---NA---
pan	6.27E+08	6.27E+08	-45418	evm.model.Herato1910.187	Leucine-rich repeats and immunoglobulin-like domains protein 2
pan	6.27E+08	6.27E+08	-45418	evm.model.Herato1910.187	Leucine-rich repeats and immunoglobulin-like domains protein 2
pan	6.3E+08	6.3E+08	34517	evm.model.Herato2001.14	hyphal wall protein 2-like
pan	6.3E+08	6.3E+08	-205483	evm.model.Herato2001.17	zinc finger protein Xfin-like
pan	6.3E+08	6.3E+08	-205483	evm.model.Herato2001.17	zinc finger protein Xfin-like
pan	6.31E+08	6.31E+08	35655	evm.model.Herato2001.29	protein KIAA0100
pan	6.32E+08	6.32E+08	23614	evm.model.Herato2001.43	chitin binding protein
pan	6.32E+08	6.32E+08	23614	evm.model.Herato2001.43	chitin binding protein
pan	6.36E+08	6.36E+08	103608	evm.model.Herato2001.133	troponin C-like
pan	6.4E+08	6.4E+08	3959	evm.model.Herato2001.242	NADPH--cytochrome P450 reductase isoform X1
pan	6.4E+08	6.4E+08	3959	evm.model.Herato2001.242	NADPH--cytochrome P450 reductase isoform X1
pan	6.42E+08	6.42E+08	-8697	evm.model.Herato2001.314	RNA binding protein fox-1 homolog 3
pan	6.47E+08	6.47E+08	27704	evm.model.Herato2001.443	protein white
pan	6.47E+08	6.47E+08	27704	evm.model.Herato2001.443	protein white
pan	6.49E+08	6.49E+08	1663	evm.model.Herato2001.501	fidgetin-like protein 1
pan	6.52E+08	6.52E+08	9215	evm.model.Herato2001.584	kinesin-related protein 6-like isoform X1
pan	6.53E+08	6.53E+08	4059	evm.model.Herato2001.585	fatty acyl-CoA reductase wat-like
pan	6.53E+08	6.53E+08	31734	evm.model.Herato2001.586	zinc transporter ZIP9

pan	6.54E+08	6.54E+08	-66828	evm.model.Herato2001.607	kanadaptin
pan	6.56E+08	6.56E+08	-167046	evm.model.Herato2001.648	synaptotagmin-7 isoform X2
pan	6.58E+08	6.58E+08	-1856	evm.model.Herato2001.699	acetyl-CoA carboxylase isoform X2
pan	6.59E+08	6.59E+08	7120	evm.model.Herato2001.718	ankyrin-3-like isoform X2
pan	6.59E+08	6.59E+08	-3235	evm.model.Herato2001.722	suppression of tumorigenicity 5 protein-like
pan	6.6E+08	6.6E+08	-120	evm.model.Herato2101.7	autophagy-related protein 13 homolog
pan	6.61E+08	6.61E+08	3890	evm.model.Herato2101.11	transmembrane protein 198
pan	6.61E+08	6.61E+08	-981	evm.model.Herato2101.20	L-galactose dehydrogenase-like
pan	6.62E+08	6.62E+08	-59944	evm.model.Herato2101.34	hypothetical protein KGM_202987
pan	6.63E+08	6.63E+08	-81094	evm.model.Herato2101.49	protein cycle isoform X3
pan	6.65E+08	6.65E+08	-30606	evm.model.Herato2101.110	probable cytochrome P450 303a1
pan	6.65E+08	6.65E+08	-30606	evm.model.Herato2101.110	probable cytochrome P450 303a1
pan	6.65E+08	6.65E+08	-40768	evm.model.Herato2101.110	probable cytochrome P450 303a1
pan	6.65E+08	6.65E+08	-40777	evm.model.Herato2101.110	probable cytochrome P450 303a1
pan	6.65E+08	6.65E+08	23014	evm.model.Herato2101.112	dual specificity mitogen-activated protein kinase kinase 1-like
pan	6.66E+08	6.66E+08	130086	evm.model.Herato2101.113	dipeptidase 1-like isoform X2
pan	6.68E+08	6.68E+08	-9271	evm.model.Herato2101.160	Intraflagellar transport protein 122
pan	6.7E+08	6.7E+08	20613	evm.model.Herato2101.201	solute carrier family 15 member 1-like
pan	6.71E+08	6.71E+08	25321	evm.model.Herato2101.220	prostatic acid phosphatase-like
pan	6.71E+08	6.71E+08	25301	evm.model.Herato2101.220	prostatic acid phosphatase-like
pan	6.71E+08	6.71E+08	-4840	evm.model.Herato2101.227	lysophospholipid acyltransferase 5
pan	6.71E+08	6.71E+08	-180	evm.model.Herato2101.229	protein enabled homolog
pan	6.71E+08	6.71E+08	5860	evm.model.Herato2101.236	uncharacterized protein LOC113404094
pan	6.71E+08	6.71E+08	2111	evm.model.Herato2101.242	uncharacterized protein LOC113404092
pan	6.72E+08	6.72E+08	-3582	evm.model.Herato2101.261	Neuronal pentraxin-2
pan	6.72E+08	6.73E+08	-9645	evm.model.Herato2101.272	DNA-binding protein D-ETS-3 isoform X1
pan	6.73E+08	6.73E+08	11168	evm.model.Herato2101.280	LIM/homeobox protein Lhx9-like

pan	6.73E+08	6.73E+08	15569	evm.model.Herato2101.283	probable NADH dehydrogenase [ubiquinone] 1 alpha subcomplex subunit 12
pan	6.75E+08	6.75E+08	-66374	evm.model.Herato2101.309	---NA---
pan	6.76E+08	6.76E+08	-517	evm.model.Herato2101.342	peptidyl-prolyl cis-trans isomerase-like 1
pan	6.76E+08	6.76E+08	-474	evm.model.Herato2101.342	peptidyl-prolyl cis-trans isomerase-like 1
pan	6.8E+08	6.8E+08	31693	evm.model.Herato2101.402	Nucleic-acid-binding protein from transposon X-element
pan	6.85E+08	6.85E+08	-17192	evm.model.Herato2101.489	mediator of RNA polymerase II transcription subunit 23
pan	6.87E+08	6.87E+08	-17529	evm.model.Herato2101.513	---NA---

notabilis-etylus hybridzone

Chr	Start	End	Distance from TSS	gene ID	gene name
pan	1605135	1605616	-90218	evm.model.Herato0101.19	nephrin
pan	1605135	1605616	-90218	evm.model.Herato0101.19	nephrin
pan	6276632	6276993	-61843	evm.model.Herato0101.116	hypothetical protein EVAR_74554_1
pan	6276632	6276993	-61843	evm.model.Herato0101.116	hypothetical protein EVAR_74554_1
pan	13657376	13657890	-615	evm.model.Herato0101.234	signal transducing adapter molecule 1
pan	13657376	13657890	-615	evm.model.Herato0101.234	signal transducing adapter molecule 1
pan	14129526	14129780	-53734	evm.model.Herato0101.241	hypothetical protein EVAR_80933_1
pan	17792572	17792960	40483	evm.model.Herato0101.296	protein spire isoform X4
pan	23940215	23940540	35192	evm.model.Herato0101.505	Zinc finger CCHC domain-containing protein 2
pan	23940215	23940540	35192	evm.model.Herato0101.505	Zinc finger CCHC domain-containing protein 2
pan	26332556	26332910	-4477	evm.model.Herato0101.568	---NA---
pan	26332556	26332910	-4477	evm.model.Herato0101.568	---NA---
pan	35110929	35111103	-26360	evm.model.Herato0101.800	dedicator of cytokinesis protein 7 isoform X2
pan	35110929	35111103	-26360	evm.model.Herato0101.800	dedicator of cytokinesis protein 7 isoform X2
pan	35995814	35996042	-8956	evm.model.Herato0101.826	glutenin, high molecular weight subunit DX5-like
pan	35995814	35996042	-8956	evm.model.Herato0101.826	glutenin, high molecular weight subunit DX5-like

pan	36982574	36982853	16807	evm.model.Herato0101.847	carbonyl reductase [NADPH] 3-like
pan	40385859	40386148	-276906	evm.model.Herato0204.28	---NA---
pan	40385859	40386148	-276906	evm.model.Herato0204.28	---NA---
pan	47403311	47403589	-27716	evm.model.Herato0209.47	uncharacterized protein LOC113395221
pan	48098230	48098500	51952	evm.model.Herato0209.67	flotillin-2 isoform X1
pan	48098230	48098500	51952	evm.model.Herato0209.67	flotillin-2 isoform X1
pan	55888488	55889005	-7048	evm.model.Herato0214.15	protein unc-119 homolog
pan	56240827	56241073	7434	evm.model.Herato0214.21	RILP protein
pan	57467577	57467997	14045	evm.model.Herato0214.54	luciferin 4-monooxygenase-like
pan	57467577	57467997	14045	evm.model.Herato0214.54	luciferin 4-monooxygenase-like
pan	58478191	58478746	-13038	evm.model.Herato0215.147	hypothetical protein
pan	58478206	58478760	-13023	evm.model.Herato0215.147	hypothetical protein
pan	59014803	59015305	104210	evm.model.Herato0215.16	fructose-bisphosphate aldolase isoform X1
pan	59014803	59015305	104210	evm.model.Herato0215.16	fructose-bisphosphate aldolase isoform X1
pan	65154561	65155053	43188	evm.model.Herato0215.143	F-box/LRR-repeat protein 7-like
pan	65310311	65310658	11605	evm.model.Herato0215.145	apolipophorin-3-like
pan	65310311	65310658	11605	evm.model.Herato0215.145	apolipophorin-3-like
pan	78052216	78053201	8166	evm.model.Herato0310.17	NADP-dependent malic enzyme
pan	78052216	78053201	8166	evm.model.Herato0310.17	NADP-dependent malic enzyme
pan	78053606	78054030	7056	evm.model.Herato0310.17	NADP-dependent malic enzyme
pan	78053606	78054030	7056	evm.model.Herato0310.17	NADP-dependent malic enzyme
pan	83389895	83390116	2077	evm.model.Herato0310.77	microtubule-associated protein tau isoform X3
pan	83389895	83390116	2077	evm.model.Herato0310.77	microtubule-associated protein tau isoform X3
pan	87334574	87335509	3228	evm.model.Herato0310.147	prefoldin subunit 6
pan	90473573	90473833	24154	evm.model.Herato0310.191	tyrosine-protein kinase Fer isoform X1
pan	94688111	94688582	-30732	evm.model.Herato0310.299	transmembrane protease serine 12-like

pan	96538512	96538733	56841	evm.model.Herato0401.5	zwei Ig domain protein zig-8-like
pan	96538512	96538733	56841	evm.model.Herato0401.5	zwei Ig domain protein zig-8-like
pan	96564053	96564533	82512	evm.model.Herato0401.5	zwei Ig domain protein zig-8-like
pan	98043098	98043785	-11104	evm.model.Herato0401.15	chondroitin sulfate synthase 1
pan	1E+08	1E+08	-7041	evm.model.Herato0401.55	serine protease snake-like
pan	1E+08	1E+08	-7041	evm.model.Herato0401.55	serine protease snake-like
pan	1.02E+08	1.02E+08	-54527	evm.model.Herato0403.3	protein groucho-like isoform X6
pan	1.02E+08	1.02E+08	-37006	evm.model.Herato0403.39	---NA---
pan	1.1E+08	1.1E+08	195837	evm.model.Herato0411.29	serine/arginine repetitive matrix protein 2
pan	1.1E+08	1.1E+08	195837	evm.model.Herato0411.29	serine/arginine repetitive matrix protein 2
pan	1.18E+08	1.18E+08	9176	evm.model.Herato0411.120	tumor necrosis factor alpha-induced protein 8-like protein isoform X1
pan	1.19E+08	1.19E+08	69103	evm.model.Herato0411.133	TP53-binding protein 1-like
pan	1.21E+08	1.21E+08	-114765	evm.model.Herato0412.1	uncharacterized protein LOC113402078 isoform X2
pan	1.21E+08	1.21E+08	-98246	evm.model.Herato0412.1	uncharacterized protein LOC113402078 isoform X2
pan	1.21E+08	1.21E+08	-98246	evm.model.Herato0412.1	uncharacterized protein LOC113402078 isoform X2
pan	1.25E+08	1.25E+08	-355	evm.model.Herato0419.41	phosphatidylinositol 3-kinase catalytic subunit type 3
pan	1.26E+08	1.26E+08	59172	evm.model.Herato0419.66	hydrocephalus-inducing protein homolog
pan	1.26E+08	1.26E+08	59172	evm.model.Herato0419.66	hydrocephalus-inducing protein homolog
pan	1.28E+08	1.28E+08	7195	evm.model.Herato0501.13	Bm8 interacting protein 2d-2
pan	1.28E+08	1.28E+08	7195	evm.model.Herato0501.13	Bm8 interacting protein 2d-2
pan	1.32E+08	1.32E+08	-13443	evm.model.Herato0503.30	Outer dense fiber protein 3-like protein 2
pan	1.32E+08	1.32E+08	-13443	evm.model.Herato0503.30	Outer dense fiber protein 3-like protein 2
pan	1.33E+08	1.33E+08	-5378	evm.model.Herato0503.64	inhibitor of growth protein 5
pan	1.33E+08	1.33E+08	-5378	evm.model.Herato0503.64	inhibitor of growth protein 5
pan	1.36E+08	1.36E+08	5356	evm.model.Herato0503.112	carboxylesterase 1E
pan	1.36E+08	1.36E+08	4900	evm.model.Herato0503.112	carboxylesterase 1E
pan	1.4E+08	1.4E+08	-23210	evm.model.Herato0503.143	protein gooseberry-neuro isoform X1

pan	1.4E+08	1.4E+08	-23210	evm.model.Herato0503.143	protein gooseberry-neuro isoform X1
pan	1.4E+08	1.4E+08	-208	evm.model.Herato0503.171	---NA---
pan	1.41E+08	1.41E+08	-3807	evm.model.Herato0503.179	---NA---
pan	1.43E+08	1.43E+08	123	evm.model.Herato0503.245	60S ribosomal protein L37a
pan	1.5E+08	1.5E+08	52170	evm.model.Herato0508.56	N6-adenosine-methyltransferase non-catalytic subunit
pan	1.5E+08	1.5E+08	52170	evm.model.Herato0508.56	N6-adenosine-methyltransferase non-catalytic subunit
pan	1.54E+08	1.54E+08	8322	evm.model.Herato0601.4	tensin-2-like isoform X3
pan	1.54E+08	1.54E+08	8322	evm.model.Herato0601.4	tensin-2-like isoform X3
pan	1.56E+08	1.56E+08	-70178	evm.model.Herato0601.10	zinc finger protein Noc
pan	1.56E+08	1.56E+08	-24580	evm.model.Herato0601.12	solute carrier family 22 member 13-like
pan	1.56E+08	1.56E+08	-24580	evm.model.Herato0601.12	solute carrier family 22 member 13-like
pan	1.6E+08	1.6E+08	15470	evm.model.Herato0601.77	corticotropin-releasing factor-binding protein
pan	1.61E+08	1.61E+08	-74536	evm.model.Herato0601.104	protein timeless homolog
pan	1.61E+08	1.61E+08	-74536	evm.model.Herato0601.104	protein timeless homolog
pan	1.66E+08	1.66E+08	-349	evm.model.Herato0606.53	serine protease gd-like
pan	1.66E+08	1.66E+08	-349	evm.model.Herato0606.53	serine protease gd-like
pan	1.69E+08	1.69E+08	-12211	evm.model.Herato0606.102	uncharacterized protein LOC113401263
pan	1.7E+08	1.7E+08	23406	evm.model.Herato0606.118	zinc finger MIZ domain-containing protein 1
pan	1.73E+08	1.73E+08	17420	evm.model.Herato0606.182	Inactive ubiquitin carboxyl-terminal hydrolase 54
pan	1.73E+08	1.73E+08	17420	evm.model.Herato0606.182	Inactive ubiquitin carboxyl-terminal hydrolase 54
pan	1.77E+08	1.77E+08	19841	evm.model.Herato0606.254	uncharacterized protein LOC113398311
pan	1.77E+08	1.77E+08	19841	evm.model.Herato0606.254	uncharacterized protein LOC113398311
pan	1.8E+08	1.8E+08	-2402	evm.model.Herato0606.293	palmitoyltransferase ZDHHC6
pan	1.8E+08	1.8E+08	-2402	evm.model.Herato0606.293	palmitoyltransferase ZDHHC6
pan	1.8E+08	1.8E+08	6899	evm.model.Herato0606.302	neurexin-4 isoform X1
pan	1.84E+08	1.84E+08	-4124	evm.model.Herato0606.417	L-dopachrome tautomerase yellow-f-like

pan	1.85E+08	1.85E+08	-18519	evm.model.Herato0606.452	uncharacterized protein LOC113391393
pan	1.92E+08	1.92E+08	-19187	evm.model.Herato0701.47	pyruvate dehydrogenase (acetyl-transferring) kinase, mitochondrial
pan	1.92E+08	1.92E+08	-19187	evm.model.Herato0701.47	pyruvate dehydrogenase (acetyl-transferring) kinase, mitochondrial
pan	1.94E+08	1.94E+08	-56988	evm.model.Herato0701.148	probable nuclear hormone receptor HR3 isoform X1
pan	1.95E+08	1.95E+08	-22104	evm.model.Herato0701.165	cullin-1
pan	2.01E+08	2.01E+08	15461	evm.model.Herato0701.307	---NA---
pan	2.01E+08	2.01E+08	15461	evm.model.Herato0701.307	---NA---
pan	2.09E+08	2.09E+08	28845	evm.model.Herato0701.469	ethanolamine-phosphate cytidylyltransferase
pan	2.09E+08	2.09E+08	28845	evm.model.Herato0701.469	ethanolamine-phosphate cytidylyltransferase
pan	2.13E+08	2.13E+08	-140734	evm.model.Herato0701.624	collagen alpha-1(XVIII) chain-like
pan	2.14E+08	2.14E+08	11752	evm.model.Herato0701.643	protein extra-macrochaetae
pan	2.14E+08	2.14E+08	11752	evm.model.Herato0701.643	protein extra-macrochaetae
pan	2.15E+08	2.15E+08	-5967	evm.model.Herato0701.645	intersectin-1 isoform X2
pan	2.2E+08	2.2E+08	-33835	evm.model.Herato0701.733	retinol dehydrogenase 11-like
pan	2.2E+08	2.2E+08	-33835	evm.model.Herato0701.733	retinol dehydrogenase 11-like
pan	2.2E+08	2.2E+08	-49650	evm.model.Herato0701.735	facilitated trehalose transporter Tret1 isoform X5
pan	2.23E+08	2.23E+08	94111	evm.model.Herato0801.5	---NA---
pan	2.31E+08	2.31E+08	1533	evm.model.Herato0801.94	nucleolin 2-like
pan	2.31E+08	2.31E+08	1533	evm.model.Herato0801.94	nucleolin 2-like
pan	2.32E+08	2.32E+08	-55423	evm.model.Herato0801.100	putative epidermal cell surface receptor isoform X1
pan	2.32E+08	2.32E+08	-103518	evm.model.Herato0801.106	---NA---
pan	2.32E+08	2.32E+08	-103482	evm.model.Herato0801.106	---NA---
pan	2.33E+08	2.33E+08	-64935	evm.model.Herato0801.107	hemicentin-1-like isoform X1
pan	2.33E+08	2.33E+08	-64935	evm.model.Herato0801.107	hemicentin-1-like isoform X1
pan	2.33E+08	2.33E+08	20983	evm.model.Herato0801.107	hemicentin-1-like isoform X1
pan	2.33E+08	2.33E+08	20983	evm.model.Herato0801.107	hemicentin-1-like isoform X1
pan	2.36E+08	2.36E+08	-94873	evm.model.Herato0801.217	neural cell adhesion molecule 1-like

pan	2.36E+08	2.36E+08	-94873	evm.model.Herato0801.217	neural cell adhesion molecule 1-like
pan	2.45E+08	2.45E+08	23816	evm.model.Herato0821.60	piggyBac transposable element-derived protein 3-like
pan	2.45E+08	2.45E+08	23816	evm.model.Herato0821.60	piggyBac transposable element-derived protein 3-like
pan	2.47E+08	2.47E+08	-96	evm.model.Herato0821.82	succinate dehydrogenase [ubiquinone] cytochrome b small subunit, mitochondrial
pan	2.53E+08	2.53E+08	162889	evm.model.Herato0901.76	uncharacterized protein LOC113401684 isoform X1
pan	2.53E+08	2.53E+08	162889	evm.model.Herato0901.76	uncharacterized protein LOC113401684 isoform X1
pan	2.57E+08	2.57E+08	-176441	evm.model.Herato0901.100	neurogenic locus protein delta
pan	2.57E+08	2.57E+08	-176441	evm.model.Herato0901.100	neurogenic locus protein delta
pan	2.57E+08	2.57E+08	-39256	evm.model.Herato0901.100	neurogenic locus protein delta
pan	2.57E+08	2.57E+08	-39256	evm.model.Herato0901.100	neurogenic locus protein delta
pan	2.58E+08	2.58E+08	4493	evm.model.Herato0901.104_evm.TU.Herato0901.106	39S ribosomal protein L19, mitochondrial
pan	2.67E+08	2.67E+08	24648	evm.model.Herato0901.281	---NA---
pan	2.67E+08	2.67E+08	24648	evm.model.Herato0901.281	---NA---
pan	2.71E+08	2.71E+08	-4865	evm.model.Herato0901.330	---NA---
pan	2.74E+08	2.74E+08	-1978	evm.model.Herato0901.374	chitinase A-like
pan	2.75E+08	2.75E+08	-15795	evm.model.Herato0904.26	---NA---
pan	2.75E+08	2.75E+08	-15795	evm.model.Herato0904.26	---NA---
pan	2.75E+08	2.75E+08	-4908	evm.model.Herato1001.9	retinal homeobox protein Rx1-like
pan	2.76E+08	2.76E+08	-15229	evm.model.Herato1001.23	---NA---
pan	2.76E+08	2.76E+08	-5572	evm.model.Herato1001.23	---NA---
pan	2.76E+08	2.76E+08	-5572	evm.model.Herato1001.23	---NA---
pan	2.76E+08	2.76E+08	-8697	evm.model.Herato1001.34	putative helix-loop-helix protein hen
pan	2.76E+08	2.76E+08	-8697	evm.model.Herato1001.34	putative helix-loop-helix protein hen
pan	2.77E+08	2.77E+08	433	evm.model.Herato1001.47_evm.TU.Herato1001.48	inner centromere protein
pan	2.77E+08	2.77E+08	433	evm.model.Herato1001.47_evm.TU.Herato1001.48	inner centromere protein

pan	2.81E+08	2.81E+08	8765	evm.model.Herato1001.118	laccase-4-like
pan	2.81E+08	2.81E+08	8765	evm.model.Herato1001.118	laccase-4-like
pan	2.84E+08	2.84E+08	-57549	evm.model.Herato1001.169	endonuclease-reverse transcriptase HmRTE-e01
pan	2.85E+08	2.85E+08	-3038	evm.model.Herato1001.176	E3 ubiquitin-protein ligase MARCH1-like
pan	2.85E+08	2.85E+08	-3038	evm.model.Herato1001.176	E3 ubiquitin-protein ligase MARCH1-like
pan	2.87E+08	2.87E+08	-1121	evm.model.Herato1003.9	serine protease inhibitor 2.1-like
pan	2.87E+08	2.87E+08	-1121	evm.model.Herato1003.9	serine protease inhibitor 2.1-like
pan	2.89E+08	2.89E+08	14476	evm.model.Herato1003.41	ankyrin repeat domain-containing protein 12
pan	2.89E+08	2.89E+08	14476	evm.model.Herato1003.41	ankyrin repeat domain-containing protein 12
pan	2.95E+08	2.95E+08	2919	evm.model.Herato1003.150	oxysterol-binding protein-related protein 1
pan	2.97E+08	2.97E+08	78407	evm.model.Herato1003.199	uncharacterized protein LOC113393764 isoform X3
pan	3.01E+08	3.01E+08	-48471	evm.model.Herato1005.77	NADH dehydrogenase [ubiquinone] flavoprotein 2, mitochondrial-like
pan	3.02E+08	3.02E+08	22552	evm.model.Herato1005.88	semaphorin-1A-like
pan	3.03E+08	3.03E+08	38109	evm.model.Herato1005.116	putative prIL-16
pan	3.03E+08	3.03E+08	-18521	evm.model.Herato1005.117	acetylcholine receptor subunit beta-like 1
pan	3.03E+08	3.03E+08	-18521	evm.model.Herato1005.117	acetylcholine receptor subunit beta-like 1
pan	3.03E+08	3.03E+08	33088	evm.model.Herato1005.222	---NA---
pan	3.04E+08	3.04E+08	20837	evm.model.Herato1005.168	septin-7 isoform X1
pan	3.09E+08	3.09E+08	10128	evm.model.Herato1007.63	glycine-rich protein DOT1-like
pan	3.09E+08	3.09E+08	10128	evm.model.Herato1007.63	glycine-rich protein DOT1-like
pan	3.1E+08	3.1E+08	41041	evm.model.Herato1007.88	peptidylglycine alpha-hydroxylating monooxygenase
pan	3.11E+08	3.11E+08	-36901	evm.model.Herato1007.97	---NA---
pan	3.12E+08	3.12E+08	-18926	evm.model.Herato1007.128	---NA---
pan	3.12E+08	3.12E+08	-18926	evm.model.Herato1007.128	---NA---
pan	3.13E+08	3.13E+08	-50168	evm.model.Herato1007.146	uncharacterized LOC106124082
pan	3.14E+08	3.14E+08	69833	evm.model.Herato1007.172	rho GTPase-activating protein conundrum isoform X1

pan	3.14E+08	3.14E+08	69833	evm.model.Herato1007.172	rho GTPase-activating protein conundrum isoform X1
pan	3.14E+08	3.14E+08	22876	evm.model.Herato1007.182	uncharacterized MFS-type transporter C09D4.1 isoform X2
pan	3.14E+08	3.14E+08	22876	evm.model.Herato1007.182	uncharacterized MFS-type transporter C09D4.1 isoform X2
pan	3.14E+08	3.14E+08	23842	evm.model.Herato1007.182	uncharacterized MFS-type transporter C09D4.1 isoform X2
pan	3.15E+08	3.15E+08	-23520	evm.model.Herato1007.187	dynein assembly factor with WDR repeat domains 1
pan	3.15E+08	3.15E+08	-23520	evm.model.Herato1007.187	dynein assembly factor with WDR repeat domains 1
pan	3.15E+08	3.15E+08	605	evm.model.Herato1007.188	lysosomal Pro-X carboxypeptidase
pan	3.15E+08	3.15E+08	605	evm.model.Herato1007.188	lysosomal Pro-X carboxypeptidase
pan	3.26E+08	3.26E+08	3641	evm.model.Herato1108.86	phospholipid phosphatase 6 isoform X1
pan	3.26E+08	3.26E+08	3641	evm.model.Herato1108.86	phospholipid phosphatase 6 isoform X1
pan	3.26E+08	3.26E+08	9332	evm.model.Herato1108.114	---NA---
pan	3.4E+08	3.4E+08	-3478	evm.model.Herato1108.457	ZZ-type zinc finger-containing protein 3
pan	3.41E+08	3.41E+08	12736	evm.model.Herato1108.489	rab GTPase-activating protein 1-like isoform X1
pan	3.41E+08	3.41E+08	12736	evm.model.Herato1108.489	rab GTPase-activating protein 1-like isoform X1
pan	3.41E+08	3.41E+08	23771	evm.model.Herato1108.491	serine/arginine repetitive matrix protein 2
pan	3.41E+08	3.41E+08	23771	evm.model.Herato1108.491	serine/arginine repetitive matrix protein 2
pan	3.43E+08	3.43E+08	-1666	evm.model.Herato1108.519	zinc finger protein 62 homolog
pan	3.45E+08	3.45E+08	-12164	evm.model.Herato1108.568	innexin inx7
pan	3.45E+08	3.45E+08	-12164	evm.model.Herato1108.568	innexin inx7
pan	3.46E+08	3.46E+08	-4186	evm.model.Herato1108.590	U7 snRNA-associated Sm-like protein LSm11
pan	3.46E+08	3.46E+08	-10261	evm.model.Herato1108.596	vesicle-trafficking protein SEC22b-B
pan	3.47E+08	3.47E+08	28480	evm.model.Herato1108.618	---NA---
pan	3.47E+08	3.47E+08	28480	evm.model.Herato1108.618	---NA---
pan	3.53E+08	3.53E+08	3246	evm.model.Herato1202.64	protein Cep89 homolog isoform X1
pan	3.59E+08	3.59E+08	-46069	evm.model.Herato1202.216	sodium- and chloride-dependent transporter XTRP3 isoform X1
pan	3.59E+08	3.59E+08	-46069	evm.model.Herato1202.216	sodium- and chloride-dependent transporter XTRP3 isoform X1
pan	3.69E+08	3.69E+08	-57092	evm.model.Herato1202.440	probable cytochrome P450 49a1

pan	3.7E+08	3.7E+08	51091	evm.model.Herato1202.451	fibroblast growth factor receptor 3-like isoform X2
pan	3.73E+08	3.73E+08	-1642	evm.model.Herato1202.504	ESF1 homolog
pan	3.73E+08	3.73E+08	-1642	evm.model.Herato1202.504	ESF1 homolog
pan	3.82E+08	3.82E+08	-523	evm.model.Herato1202.756	SWI/SNF-related matrix-associated actin-dependent regulator of chromatin subfamily A containing DEAD/H box 1 homolog
pan	3.82E+08	3.82E+08	-523	evm.model.Herato1202.756	SWI/SNF-related matrix-associated actin-dependent regulator of chromatin subfamily A containing DEAD/H box 1 homolog
pan	3.83E+08	3.83E+08	55028	evm.model.Herato1202.776	mucin-5AC isoform X1
pan	3.83E+08	3.83E+08	55028	evm.model.Herato1202.776	mucin-5AC isoform X1
pan	3.84E+08	3.84E+08	38770	evm.model.Herato1202.791	complement component 1 Q subcomponent-binding protein, mitochondrial
pan	3.84E+08	3.84E+08	38770	evm.model.Herato1202.791	complement component 1 Q subcomponent-binding protein, mitochondrial
pan	3.86E+08	3.86E+08	24826	evm.model.Herato1301.6	protein vestigial
pan	3.86E+08	3.86E+08	6431	evm.model.Herato1301.12	---NA---
pan	3.86E+08	3.86E+08	6431	evm.model.Herato1301.12	---NA---
pan	3.86E+08	3.86E+08	931	evm.model.Herato1301.12	---NA---
pan	3.86E+08	3.86E+08	931	evm.model.Herato1301.12	---NA---
pan	3.88E+08	3.88E+08	-6907	evm.model.Herato1301.33	protein vein isoform X1
pan	3.88E+08	3.88E+08	-6907	evm.model.Herato1301.33	protein vein isoform X1
pan	3.9E+08	3.9E+08	-24813	evm.model.Herato1301.55	paxillin homolog 1 isoform X1
pan	3.9E+08	3.9E+08	-20965	evm.model.Herato1301.57	elongation of very long chain fatty acids protein 6
pan	3.9E+08	3.9E+08	-20965	evm.model.Herato1301.57	elongation of very long chain fatty acids protein 6
pan	3.91E+08	3.91E+08	-8017	evm.model.Herato1301.89	cuticular protein RR-1 motif 47 precursor
pan	3.91E+08	3.91E+08	-8015	evm.model.Herato1301.89	cuticular protein RR-1 motif 47 precursor
pan	3.97E+08	3.97E+08	-5341	evm.model.Herato1301.242	glycine dehydrogenase (decarboxylating), mitochondrial
pan	4.07E+08	4.07E+08	52834	evm.model.Herato1301.481	uncharacterized protein LOC117982178 isoform X1
pan	4.07E+08	4.07E+08	52834	evm.model.Herato1301.481	uncharacterized protein LOC117982178 isoform X1

pan	4.11E+08	4.11E+08	71842	evm.model.Herato1301.557	neurobeachin isoform X1
pan	4.11E+08	4.11E+08	71842	evm.model.Herato1301.557	neurobeachin isoform X1
pan	4.13E+08	4.13E+08	-192819	evm.model.Herato1301.581	ubinuclein-1 isoform X3
pan	4.14E+08	4.14E+08	10715	evm.model.Herato1301.589	ras-related protein Rab-30
pan	4.17E+08	4.17E+08	-3	evm.model.Herato1301.628	AF4/FMR2 family member 1 isoform X1
pan	4.17E+08	4.17E+08	-3	evm.model.Herato1301.628	AF4/FMR2 family member 1 isoform X1
pan	4.18E+08	4.18E+08	5454	evm.model.Herato1301.664	probable cytochrome P450 301a1, mitochondrial
pan	4.18E+08	4.18E+08	5454	evm.model.Herato1301.664	probable cytochrome P450 301a1, mitochondrial
pan	4.19E+08	4.19E+08	-183997	evm.model.Herato1301.673	heat shock protein 21.3
pan	4.2E+08	4.2E+08	1081	evm.model.Herato1301.693	protein goliath isoform X2
pan	4.21E+08	4.21E+08	-33041	evm.model.Herato1301.721	---NA---
pan	4.28E+08	4.28E+08	-72647	evm.model.Herato1408.25	esterase FE4-like
pan	4.28E+08	4.28E+08	-72647	evm.model.Herato1408.25	esterase FE4-like
pan	4.29E+08	4.29E+08	128059	evm.model.Herato1408.27	---NA---
pan	4.3E+08	4.3E+08	-21879	evm.model.Herato1408.48	reverse transcriptase
pan	4.32E+08	4.32E+08	28860	evm.model.Herato1408.71	putative phosphatidate phosphatase
pan	4.36E+08	4.36E+08	-80444	evm.model.Herato1408.165	Outer dense fiber protein 3-like protein 2
pan	4.36E+08	4.36E+08	-80444	evm.model.Herato1408.165	Outer dense fiber protein 3-like protein 2
pan	4.43E+08	4.43E+08	57850	evm.model.Herato1411.54	transcription factor TFIIIB component B''
pan	4.45E+08	4.45E+08	26941	evm.model.Herato1411.107	receptor-type tyrosine-protein phosphatase kappa isoform X1
pan	4.45E+08	4.45E+08	26941	evm.model.Herato1411.107	receptor-type tyrosine-protein phosphatase kappa isoform X1
pan	4.45E+08	4.45E+08	-754	evm.model.Herato1411.114	ras-like GTP-binding protein Rho1 isoform X2
pan	4.45E+08	4.45E+08	-754	evm.model.Herato1411.114	ras-like GTP-binding protein Rho1 isoform X2
pan	4.48E+08	4.48E+08	-35295	evm.model.Herato1411.184	endocuticle structural glycoprotein SgAbd-3-like
pan	4.48E+08	4.48E+08	-35295	evm.model.Herato1411.184	endocuticle structural glycoprotein SgAbd-3-like
pan	4.53E+08	4.53E+08	-18148	evm.model.Herato1411.255	general odorant-binding protein 1-like
pan	4.53E+08	4.53E+08	-18148	evm.model.Herato1411.255	general odorant-binding protein 1-like

pan	4.53E+08	4.53E+08	72512	evm.model.Herato1502.1	60S ribosomal protein L38
pan	4.53E+08	4.53E+08	72512	evm.model.Herato1502.1	60S ribosomal protein L38
pan	4.54E+08	4.54E+08	-9086	evm.model.Herato1505.20	peptidyl-tRNA hydrolase ICT1, mitochondrial
pan	4.54E+08	4.54E+08	-9086	evm.model.Herato1505.20	peptidyl-tRNA hydrolase ICT1, mitochondrial
pan	4.6E+08	4.6E+08	-19207	evm.model.Herato1505.143_evm.TU.Herato1505.146	nudC domain-containing protein 3
pan	4.6E+08	4.6E+08	-19232	evm.model.Herato1505.143_evm.TU.Herato1505.146	nudC domain-containing protein 3
pan	4.68E+08	4.68E+08	-87459	evm.model.Herato1507.78	nudix hydrolase 8-like
pan	4.68E+08	4.68E+08	-87459	evm.model.Herato1507.78	nudix hydrolase 8-like
pan	4.68E+08	4.68E+08	-6697	evm.model.Herato1507.89	uncharacterized protein LOC113400528
pan	4.68E+08	4.68E+08	-6697	evm.model.Herato1507.89	uncharacterized protein LOC113400528
pan	4.71E+08	4.71E+08	-4022	evm.model.Herato1507.150	---NA---
pan	4.72E+08	4.72E+08	-28094	evm.model.Herato1507.156	brain-specific angiogenesis inhibitor 1-associated protein 2 isoform X3
pan	4.72E+08	4.72E+08	-55977	evm.model.Herato1507.163	acidic repeat-containing protein-like
pan	4.74E+08	4.74E+08	-67192	evm.model.Herato1507.215	Regulator of G-protein signaling 7
pan	4.77E+08	4.77E+08	-4291	evm.model.Herato1507.250	synaptogyrin
pan	4.78E+08	4.78E+08	-26188	evm.model.Herato1507.272	E3 ubiquitin-protein ligase RING1
pan	4.88E+08	4.88E+08	-26769	evm.model.Herato1524.18	sodium-independent sulfate anion transporter-like
pan	4.88E+08	4.88E+08	-859	evm.model.Herato1601.8	serine/arginine repetitive matrix protein 1
pan	4.88E+08	4.88E+08	-859	evm.model.Herato1601.8	serine/arginine repetitive matrix protein 1
pan	4.89E+08	4.89E+08	11703	evm.model.Herato1601.32	piggyBac transposable element-derived protein 4-like
pan	4.89E+08	4.89E+08	11703	evm.model.Herato1601.32	piggyBac transposable element-derived protein 4-like
pan	4.9E+08	4.91E+08	-120817	evm.model.Herato1601.49	acyl-CoA:lysophosphatidylglycerol acyltransferase 1-like
pan	4.93E+08	4.93E+08	-19375	evm.model.Herato1601.58	uncharacterized LOC106121622 precursor
pan	4.93E+08	4.93E+08	-19375	evm.model.Herato1601.58	uncharacterized LOC106121622 precursor

pan	4.98E+08	4.98E+08	33467	evm.model.Herato1601.109	fasciculation and elongation protein zeta-2
pan	5.02E+08	5.02E+08	-25421	evm.model.Herato1603.127	---NA---
pan	5.03E+08	5.03E+08	-5212	evm.model.Herato1603.47	CWF19-like protein 2 homolog
pan	5.03E+08	5.03E+08	-32230	evm.model.Herato1603.58	mucin-5AC isoform X1
pan	5.03E+08	5.03E+08	-32230	evm.model.Herato1603.58	mucin-5AC isoform X1
pan	5.05E+08	5.05E+08	5489	evm.model.Herato1603.84	uncharacterized protein LOC113399111
pan	5.05E+08	5.05E+08	5489	evm.model.Herato1603.84	uncharacterized protein LOC113399111
pan	5.06E+08	5.06E+08	-93039	evm.model.Herato1603.89	apoptosis-stimulating of p53 protein 1 isoform X4
pan	5.06E+08	5.06E+08	-93039	evm.model.Herato1603.89	apoptosis-stimulating of p53 protein 1 isoform X4
pan	5.15E+08	5.15E+08	15979	evm.model.Herato1605.121	---NA---
pan	5.15E+08	5.15E+08	15950	evm.model.Herato1605.121	---NA---
pan	5.16E+08	5.16E+08	-14911	evm.model.Herato1605.159	ADP-dependent glucokinase
pan	5.22E+08	5.22E+08	92188	evm.model.Herato1701.63	nephrin-like isoform X1
pan	5.33E+08	5.33E+08	15094	evm.model.Herato1701.306	cytoplasmic dynein 2 heavy chain 1-like
pan	5.33E+08	5.33E+08	15094	evm.model.Herato1701.306	cytoplasmic dynein 2 heavy chain 1-like
pan	5.33E+08	5.33E+08	24357	evm.model.Herato1701.315	SUN domain-containing protein 3-like
pan	5.33E+08	5.33E+08	24357	evm.model.Herato1701.315	SUN domain-containing protein 3-like
pan	5.34E+08	5.34E+08	-930	evm.model.Herato1701.334	G1/S-specific cyclin-D3
pan	5.34E+08	5.34E+08	-930	evm.model.Herato1701.334	G1/S-specific cyclin-D3
pan	5.44E+08	5.44E+08	-29870	evm.model.Herato1705.13	fat-like cadherin-related tumor suppressor homolog
pan	5.44E+08	5.44E+08	-29870	evm.model.Herato1705.13	fat-like cadherin-related tumor suppressor homolog
pan	5.46E+08	5.46E+08	-17038	evm.model.Herato1705.76	translocon-associated protein subunit delta
pan	5.46E+08	5.46E+08	-17038	evm.model.Herato1705.76	translocon-associated protein subunit delta
pan	5.52E+08	5.52E+08	-5605	evm.model.Herato1712.7	unnamed protein product
pan	5.52E+08	5.52E+08	-5605	evm.model.Herato1712.7	unnamed protein product
pan	5.53E+08	5.53E+08	-12378	evm.model.Herato1801.20	RNA-directed DNA polymerase from mobile element jockey
pan	5.53E+08	5.53E+08	39352	evm.model.Herato1801.17	basic juvenile hormone-suppressible protein 1-like

pan	5.57E+08	5.57E+08	-66615	evm.model.Herato1801.97	Protein YIF1B-B
pan	5.57E+08	5.57E+08	39819	evm.model.Herato1801.108	leucine zipper putative tumor suppressor 2 homolog isoform X1
pan	5.57E+08	5.57E+08	-11461	evm.model.Herato1801.111	nucleolin-like
pan	5.58E+08	5.58E+08	19619	novel_exon_214	Retrovirus-related Pol polyprotein from type-2 retrotransposable element R2DM; Endonuclease
pan	5.58E+08	5.58E+08	19619	novel_exon_214	Retrovirus-related Pol polyprotein from type-2 retrotransposable element R2DM; Endonuclease
pan	5.59E+08	5.59E+08	-10258	evm.model.Herato1801.147	hybrid signal transduction histidine kinase D-like
pan	5.59E+08	5.59E+08	-10258	evm.model.Herato1801.147	hybrid signal transduction histidine kinase D-like
pan	5.63E+08	5.63E+08	-19761	evm.model.Herato1805.41	hexokinase type 2 isoform X1
pan	5.64E+08	5.64E+08	48755	evm.model.Herato1805.55	protein couch potato isoform X1
pan	5.66E+08	5.66E+08	-64750	evm.model.Herato1805.81	toll-like receptor Tollo
pan	5.66E+08	5.66E+08	-64750	evm.model.Herato1805.81	toll-like receptor Tollo
pan	5.67E+08	5.67E+08	99589	evm.model.Herato1805.88	fatty acid synthase-like
pan	5.67E+08	5.67E+08	99589	evm.model.Herato1805.88	fatty acid synthase-like
pan	5.68E+08	5.68E+08	-1592	evm.model.Herato1805.95	exportin-4-like
pan	5.73E+08	5.73E+08	-28576	evm.model.Herato1805.160	muscarinic acetylcholine receptor DM1
pan	5.73E+08	5.73E+08	-28616	evm.model.Herato1805.166	uncharacterized protein LOC116769663 isoform X2
pan	5.73E+08	5.73E+08	-28620	evm.model.Herato1805.166	uncharacterized protein LOC116769663 isoform X2
pan	5.73E+08	5.73E+08	-1946	evm.model.Herato1805.169	tubulin-folding cofactor B
pan	5.73E+08	5.73E+08	-1946	evm.model.Herato1805.169	tubulin-folding cofactor B
pan	5.75E+08	5.75E+08	-21479	evm.model.Herato1805.219	organic cation transporter protein-like
pan	5.76E+08	5.76E+08	-37298	evm.model.Herato1805.234	---NA---
pan	5.76E+08	5.76E+08	-37298	evm.model.Herato1805.234	---NA---
pan	5.77E+08	5.77E+08	-13102	evm.model.Herato1805.247	inhibin beta B chain
pan	5.83E+08	5.83E+08	9255	evm.model.Herato1807.37	catenin delta-2 isoform X1
pan	5.83E+08	5.83E+08	9255	evm.model.Herato1807.37	catenin delta-2 isoform X1
pan	5.83E+08	5.83E+08	35525	evm.model.Herato1807.37	catenin delta-2 isoform X1

pan	5.83E+08	5.83E+08	12939	evm.model.Herato1807.38	hypothetical protein evm_001344
pan	5.83E+08	5.84E+08	-7586	evm.model.Herato1807.41	latrophilin Cirl isoform X3
pan	5.85E+08	5.85E+08	-7368	evm.model.Herato1807.68	zinc finger protein rotund-like
pan	5.86E+08	5.86E+08	4417	evm.model.Herato1807.87	transcriptional regulator ATRX-like
pan	5.86E+08	5.86E+08	4417	evm.model.Herato1807.87	transcriptional regulator ATRX-like
pan	5.87E+08	5.87E+08	-2843	evm.model.Herato1807.114	Transcription factor Adf-1
pan	5.87E+08	5.87E+08	-2843	evm.model.Herato1807.114	Transcription factor Adf-1
pan	5.9E+08	5.9E+08	34105	evm.model.Herato1901.117	---NA---
pan	5.93E+08	5.93E+08	-33229	evm.model.Herato1901.66	mycosubtilin synthase subunit C
pan	5.93E+08	5.93E+08	-33229	evm.model.Herato1901.66	mycosubtilin synthase subunit C
pan	5.95E+08	5.95E+08	243	evm.model.Herato1901.115	uncharacterized protein LOC112056053
pan	6.03E+08	6.03E+08	4305	evm.model.Herato1904.140	nucleolar protein 12
pan	6.03E+08	6.03E+08	4305	evm.model.Herato1904.140	nucleolar protein 12
pan	6.03E+08	6.03E+08	-10352	evm.model.Herato1904.141	uncharacterized protein LOC116766284
pan	6.04E+08	6.04E+08	5209	evm.model.Herato1904.163	---NA---
pan	6.04E+08	6.04E+08	5209	evm.model.Herato1904.163	---NA---
pan	6.08E+08	6.08E+08	1112	evm.model.Herato1904.263	Outer dense fiber protein 3-like protein 2
pan	6.09E+08	6.09E+08	-1836	evm.model.Herato1904.269	nose resistant to fluoxetine protein 6-like
pan	6.09E+08	6.09E+08	-1836	evm.model.Herato1904.269	nose resistant to fluoxetine protein 6-like
pan	6.11E+08	6.11E+08	-4155	evm.model.Herato1905.5	4F2 cell-surface antigen heavy chain-like
pan	6.11E+08	6.11E+08	-17654	evm.model.Herato1905.8	ATP-binding cassette sub-family G member 4-like isoform X2
pan	6.11E+08	6.11E+08	-30872	evm.model.Herato1905.8	ATP-binding cassette sub-family G member 4-like isoform X2
pan	6.11E+08	6.11E+08	-30872	evm.model.Herato1905.8	ATP-binding cassette sub-family G member 4-like isoform X2
pan	6.12E+08	6.12E+08	77188	evm.model.Herato1906.21	headcase protein
pan	6.12E+08	6.12E+08	77188	evm.model.Herato1906.21	headcase protein
pan	6.14E+08	6.14E+08	-12546	evm.model.Herato1908.10	phosphoinositide 3-kinase adapter protein 1 isoform X2
pan	6.14E+08	6.14E+08	-12546	evm.model.Herato1908.10	phosphoinositide 3-kinase adapter protein 1 isoform X2

pan	6.18E+08	6.18E+08	-6713	evm.model.Herato1908.94	39S ribosomal protein L48, mitochondrial
pan	6.18E+08	6.18E+08	-6713	evm.model.Herato1908.94	39S ribosomal protein L48, mitochondrial
pan	6.19E+08	6.19E+08	-1200	evm.model.Herato1910.19	RNA polymerase II transcriptional coactivator
pan	6.2E+08	6.2E+08	26423	evm.model.Herato1910.61	protein turtle isoform X1
pan	6.21E+08	6.21E+08	17658	evm.model.Herato1910.102	dnaJ homolog subfamily C member 10-like
pan	6.21E+08	6.21E+08	17658	evm.model.Herato1910.102	dnaJ homolog subfamily C member 10-like
pan	6.22E+08	6.22E+08	-60280	evm.model.Herato1910.108	uncharacterized protein LOC117984558
pan	6.24E+08	6.24E+08	9449	evm.model.Herato1910.152	etoposide-induced protein 2.4 homolog
pan	6.24E+08	6.24E+08	-43715	evm.model.Herato1910.152	etoposide-induced protein 2.4 homolog
pan	6.29E+08	6.29E+08	4962	evm.model.Herato1910.225	protein borderless
pan	6.3E+08	6.3E+08	-214983	evm.model.Herato2001.17	zinc finger protein Xfin-like
pan	6.3E+08	6.3E+08	-214983	evm.model.Herato2001.17	zinc finger protein Xfin-like
pan	6.33E+08	6.33E+08	885	evm.model.Herato2001.72_evm.TU.Herato2001.73	THO complex subunit 6
pan	6.33E+08	6.33E+08	885	evm.model.Herato2001.72_evm.TU.Herato2001.73	THO complex subunit 6
pan	6.42E+08	6.42E+08	-353	evm.model.Herato2001.326	probable cytosolic iron-sulfur protein assembly protein Ciao1 isoform X1
pan	6.42E+08	6.42E+08	-353	evm.model.Herato2001.326	probable cytosolic iron-sulfur protein assembly protein Ciao1 isoform X1
pan	6.43E+08	6.43E+08	173	evm.model.Herato2001.383	RNA-binding protein Musashi-like Rbp6
pan	6.44E+08	6.44E+08	-46274	evm.model.Herato2001.394	uncharacterized protein LOC113400031
pan	6.44E+08	6.44E+08	63017	evm.model.Herato2001.394	uncharacterized protein LOC113400031
pan	6.52E+08	6.52E+08	25421	evm.model.Herato2001.582	putative B-cell lymphoma 3-encoded protein
pan	6.65E+08	6.65E+08	44929	evm.model.Herato2101.108	alpha-1,3-mannosyl-glycoprotein 4-beta-N-acetylglucosaminyltransferase A-like
pan	6.65E+08	6.65E+08	44929	evm.model.Herato2101.108	alpha-1,3-mannosyl-glycoprotein 4-beta-N-acetylglucosaminyltransferase A-like

pan	6.72E+08	6.72E+08	21878	evm.model.Herato2101.260	coiled-coil domain-containing protein AGAP005037 isoform X1
pan	6.72E+08	6.72E+08	21878	evm.model.Herato2101.260	coiled-coil domain-containing protein AGAP005037 isoform X1
pan	6.83E+08	6.83E+08	-4907	evm.model.Herato2101.451	otoancorin like protein

Table S6: unique ATAC-seq peaks and their closest genes

	Fst					sweep			
	notabilis vs etylus	demophoon vs hydara	demophoon vs favorinus	demophoon vs etylus	demophoon vs chestertonii	notabilis	etylus	demophoon	hydara
total	464	720	1286	1315	2725	492	494	746	746
**0-0.1**	410(88,4%)	626(87%)	930(72,4%)	858(65,3%)	324(11,9%)	491(99,8%)	492(99,6%)	742(99,5%)	738(99%)
**0.1-0.2**	28(6,1%)	41(5,7%)	**181(14,1%)****	**213(16,2%)***	737(27,1%)	1(0,3%)	0(0%)	0(0%)	**6(0,9%)****
**0.2-0.3**	8(1,8%)	**26(3,7%)*****	**78(6,1%)*****	**96(7,4%)*****	756(27,8%)	0(0%)	2(0,5%)	3(0,5%)	0(0%)
**0.3-0.4**	6(1,3%)	11(1,6%)	**52(4,1%)*****	**70(5,4%)*****	494(18,2%)	0(0%)	0(0%)	1(0,2%)	2(0,3%)
**0.4-0.5**	**6(1,3%)***	7(1%)	16(1,3%)	**32(2,5%)****	234(8,6%)	0(0%)	0(0%)	0(0%)	0(0%)
**0.5-0.6**	3(0,7%)	3(0,5%)	**19(1,5%)*****	**22(1,7%)*****	93(3,5%)	0(0%)	0(0%)	0(0%)	0(0%)
**0.6-0.7**	2(0,5%)	**5(0,7%)****	3(0,3%)	**13(1%)****	57(2,1%)	0(0%)	0(0%)	0(0%)	0(0%)
**0.7-0.8**	1(0,3%)	1(0,2%)	3(0,3%)	5(0,4%)	24(0,9%)	0(0%)	0(0%)	0(0%)	0(0%)
**0.8-0.9**	0(0%)	0(0%)	3(0,3%)	2(0,2%)	4(0,2%)	0(0%)	0(0%)	0(0%)	0(0%)
**0.9-1**	0(0%)	0(0%)	1(0,1%)	**4(0,4%)****	2(0,1%)	0(0%)	0(0%)	0(0%)	0(0%)

TableS7: Custom binomial test results, the significant one are in bold
